# Physiological effects of intraperitoneal versus subcutaneous insulin infusion in patients with diabetes mellitus type 1: A systematic review and meta-analysis

**DOI:** 10.1371/journal.pone.0249611

**Published:** 2021-04-13

**Authors:** Ilze Dirnena-Fusini, Marte Kierulf Åm, Anders Lyngvi Fougner, Sven Magnus Carlsen, Sverre Christian Christiansen

**Affiliations:** 1 Department of Clinical and Molecular Medicine, Faculty of Medicine and Health Sciences, Norwegian University of Science and Technology, Trondheim, Norway; 2 Department of Endocrinology, St. Olav’s University Hospital, Trondheim, Norway; 3 Department of Engineering Cybernetics, Faculty of Information Technology and Electrical Engineering, Norwegian University of Science and Technology, Trondheim, Norway; Medical University of Vienna, AUSTRIA

## Abstract

The intraperitoneal route of administration accounts for less than 1% of insulin treatment regimes in patients with diabetes mellitus type 1 (DM1). Despite being used for decades, a systematic review of various physiological effects of this route of insulin administration is lacking. Thus, the aim of this systematic review was to identify the physiological effects of continuous intraperitoneal insulin infusion (CIPII) compared to those of continuous subcutaneous insulin infusion (CSII) in patients with DM1. Four databases (EMBASE, PubMed, Scopus and CENTRAL) were searched beginning from the inception date of each database to 10^th^ of July 2020, using search terms related to intraperitoneal and subcutaneous insulin administration. Only studies comparing CIPII treatment (≥ 1 month) with CSII treatment were included. Primary outcomes were long-term glycaemic control (after ≥ 3 months of CIPII inferred from glycated haemoglobin (HbA1c) levels) and short-term (≥ 1 day for each intervention) measurements of insulin dynamics in the systematic circulation. Secondary outcomes included all reported parameters other than the primary outcomes. The search identified a total of 2242 records; 39 reports from 32 studies met the eligibility criteria. This meta-analysis focused on the most relevant clinical end points; the mean difference (MD) in HbA1c levels during CIPII was significantly lower than during CSII (MD = -6.7 mmol/mol, [95% CI: -10.3 –-3.1]; in percentage: MD = -0.61%, [95% CI: -0.94 –- 0.28], p = 0.0002), whereas fasting blood glucose levels were similar (MD = 0.20 mmol/L, [95% CI: -0.34–0.74], p = 0.47; in mg/dL: MD = 3.6 mg/dL, [95% CI: -6.1–13.3], p = 0.47). The frequencies of severe hypo- and hyper-glycaemia were reduced. The fasting insulin levels were significantly lower during CIPII than during CSII (MD = 16.70 pmol/L, [95% CI: -23.62 –-9.77], p < 0.0001). Compared to CSII treatment, CIPII treatment improved overall glucose control and reduced fasting insulin levels in patients with DM1.

## Introduction

Patients with diabetes mellitus type 1 (DM1) lack endogenous insulin and are completely dependent on external insulin delivery. This is usually accomplished by subcutaneous (SC) delivery, either by multiple daily injections (MDI) or via continuous subcutaneous insulin infusion (CSII). Despite considerable efforts, most patients with DM1 experience frequent episodes of hyper- and hypoglycaemia, and they often fail to keep their glucose levels within the desired range. Hence, alternative treatment options to achieve better glucose control are desired. Many research groups have explored whether intraperitoneal (IP) insulin delivery can improve overall glucose control compared with SC insulin administration.

In healthy subjects, insulin is secreted from the pancreas to the liver via the portal vein. In the portal vein, the insulin concentration can be several times higher than in the systemic circulation [1, 2]. Hepatic insulin extraction from the portal vein during the first pass through the liver varies between 20% and 80% [3]. After SC insulin injections, the systemic and portal vein insulin concentrations become more or less equalised, resulting in systemic hyperinsulinemia and hepatic hypoinsulinemia as compared to the normal physiological conditions in healthy subjects [4, 5]. Furthermore, the SC route is hampered by a variable and slow insulin absorption rate and, consequently, a slow onset of its glucose lowering effects [1]. The slow modification of glucose levels after administration of SC boluses of insulin is also a challenge in the development of an artificial pancreas (AP) that relies on SC administration [6].

Animal trials have shown that IP insulin administration appears to be more physiological than SC insulin administration [7], as a substantial percentage of IP administered insulin is primarily absorbed via the portal vein [8] and at a faster rate [9]. Accordingly, IP insulin administration is a promising means of achieving improved glucose control compared to the SC route [10]. Furthermore, IP insulin delivery may also prove to be an advantage for the implementation and use of an AP [6]. However, being an invasive treatment, IP administration of insulin also has some disadvantages, although the overall risk profile as compared to SC insulin treatment remains unknown.

We hypothesised that the CIPII normalises metabolic processes in the patients with DM1 compared to the CSII. The aim of this systematic review was to identify possible differences in the physiological effects related to CIPII versus CSII insulin administration in patients with DM1. More specifically, we aimed to determine the following: (i) whether there were benefits and harms associated with CIPII versus CSII insulin administration in patients with DM1; and (ii) whether there were methodological characteristics that could explain the divergent outcomes of previous studies. There is a lot of available information about IP versus SC insulin administration; however, more explicitly, studies with comparisons with CIPII versus CSII are limited. Meanwhile, these studies present a wide range of metabolic analyses; therefore, in this systematic review, we included clinically most relevant physiological changes during CIPII versus CSII-treatment periods.

## Materials and methods

The protocol followed the PRISMA and Cochrane Handbook guidelines and was registered with the International Prospective Register of Systematic Reviews (PROSPERO) on the 30^th^ of June 2016 (registration number CRD42016040124).

### Search strategy

Systematic searches were performed in PubMed, EMBASE (Medline/Ovid), The Cochrane Library’s CENTRAL database (Wiley Online Library), and Scopus. A librarian assisted in developing the search strategy (S1 Table in S1 File). Searches for trial protocols registered with ClinicalTrials.gov and the International Standard Randomized Controlled Trial Number (ISRCTN) registry were also performed. Furthermore, the International Clinical Trials Registry Platform Search Portal was used to search for ongoing or recently completed trials. Dissertation Abstracts, Electronic Thesis Online Service (EthOS) and Network Digital Library of Theses and Dissertations database were additionally searched. For all relevant material, all references were checked to identify additional material (grey literature). The last search was performed on the 10^th^ of July 2020.

All abstracts and titles of articles from the systematic search were uploaded to Distiller SR software. Two reviewers (IDF and MKÅ) independently screened the reports and abstracts based on predefined inclusion and exclusion criteria. During the data evaluation, we decided to restrict the results to the effects of CSII and CIPII only (see the ‘Changes in the systematic review compared to the Protocol’ section in the S1 File). When any disagreement occurred, two consultants with expertise in endocrinology (SCC and SMC) independently evaluated the material.

### Eligibility criteria

#### Types of studies

All reports and abstracts from studies addressing the physiological effects of CIPII versus CSII in DM1 patients were included, including controlled trials, observational studies, case series (> 1 case), case reports (single case), as well as abstracts from clinical and scientific conference presentations.

*Participants and interventions*. Studies were determined to be eligible if CIPII treatment was compared to CSII treatment in DM1 patients. The CIPII treatment had to exceed one month in duration (including the wound healing period after establishing the abdominal port for insulin delivery). The minimum follow-up for the evaluation of glycated haemoglobin A1c (HbA1c) levels was set to three months, as HbA1c reflects the average glucose levels of the previous 120 days (the average erythrocyte life span) [11]. Consequently, as the follow-up was less than three months in two studies, they were excluded from the HbA1c analyses [12, 13].

*Outcome measures*. Any outcome reported in any of the included studies was included in the systematic review.

The primary outcomes included the following: (1) glycaemic control (HbA1c levels, fasting blood glucose (fasting BG) levels, hypoglycaemia, and hyperglycaemia); and (2) insulin levels (fasting insulin levels, time to reach peak insulin concentrations, maximum insulin levels, and time until insulin levels return to the basal level) and the mean daily insulin dose.

The secondary outcomes included any reported variable other than those listed in the primary outcomes. These included the following: (1) glycaemic control (self-monitoring of blood glucose (SMBG), mean daily BG levels, time spent in normoglycaemia, and glucose variability); (2) intermediate metabolites (triglycerides, cholesterol, free fatty acids, lactate, ketone bodies, and apolipoproteins); (3) counterregulatory hormones and other hormones (glucagon, catecholamines, growth hormone, insulin-like growth hormones, and binding proteins); (4) other metabolic outcomes (levels of anti-insulin antibodies (AIA), sex hormone binding globulin (SHBG), and plasminogen activator inhibitor-1 (PAI– 1)); and (5) any technical and/or physiological complications reported during CIPII treatment.

### Data extraction

IDF and SCC independently extracted the data from each eligible study, including information on trial design and experimental interventions, the type of comparator, insulin dosage, the frequency and duration of treatment, patient characteristics (age, sex, mean duration of diabetes, types of other symptoms, and the mode of insulin delivery), number of included patients, duration of follow-up, and inclusion and exclusion criteria. When we encountered missing information, we contacted the authors for further clarification. Five out of ten authors responded to our request for information, although only two of them provided informative answers.

### Statistical analysis

Web-based tools were used to convert glucose concentration from mg/dL to mmol/L, HbA1c from percentages to mmol/mol [14], insulin levels from mU/L to pmol/L [15], and lipid levels from mg/dL to mmol/L [16].

Data were extracted from text, tables, and figures in the included reports. Data that were extracted from the figures of three studies [17–19] may be inaccurate due to the low-resolution of the figures. Data presentations in which no p-values were reported were assigned to the ‘p-value not calculated’ category when comparing CIPII to CSII periods/treated patients (S2.1–2.5 and S2.9–2.13 Tables in S1 File). Raw data and data for individual participants were extracted from six studies [18–23], and the standard deviations (SDs) for mean HbA1c, SMBG, insulin, cholesterol, or triglyceride levels were calculated using IBM Statistical Package for the Social Sciences (SPSS) Statistics 26.

If a study reported measurements from several time-points during the CIPII and/or CSII periods, the data from the final time-point during the CIPII and/or CSII period was selected for the meta-analysis.

Continuous outcomes were measured and analysed as mean differences and 95% confidence intervals (MD, 95% CI); skewed data and non-quantitative data were presented descriptively [24]. A meta-analysis was performed on the primary outcomes including HbA1c, fasting BG, and fasting insulin levels, and daily insulin dose, and the secondary outcomes including SMBG, cholesterol, and triglyceride levels using STATA software (Stata Corp. 2019. Stata Statistical Software: Release 16. College Station, TX: Stata Corp LLC) (Figs 2–7 and S1 –S7c Figs in S1 File). A meta-analysis could not be performed for the other secondary outcomes (levels of free fatty acids, lactate, ketone bodies, apolipoproteins, glucagon, adrenaline, noradrenaline, growth hormone, insulin-like growth factor, insulin-like growth factor binding proteins, sex hormone binding globulin, anti-insulin antibodies, and plasminogen activator inhibitor 1) due to the diversity in the presentation of the results (e.g., mean values, mean difference, only p-values, or only text descriptions without exact numbers) (S2.1 –S2.14 Tables in S1 File).

When required, the SDs were derived from the available standard errors of the mean (SEM) and the number of participants (S2.9 –S2.14 Tables in S1 File) using the calculator in the Review Manager software (RevMan, version 5.3). When the outcome variables were continuous measurements, the mean difference (MD) was used as the effect size. The heterogeneity was estimated with random effects models and restricted maximum likelihood as the analysis model. The heterogeneity was estimated by the I^2^ statistic and categorised as follows: a) heterogeneity that might not be important (0–40%), b) may represent moderate or substantial heterogeneity (40–75%), or c) considerable heterogeneity (75–100%). However, the importance of the observed value depends on the magnitude and direction of the effects and the strength of the evidence for heterogeneity [25]. Each study was weighted using STATA software for continuous outcome variables, based on the SD and the sample size of the study. This weighting determined how much each individual study contributed to the pooled results estimates [26].

### Assessment of the risk of bias

For randomised comparisons, the Cochrane collaboration tools were used to assess the random sequence generation, allocation concealment, the blinding of participants and personnel, the blinding of the outcome assessment, the presence of incomplete outcome data, and selective reporting and ‘other bias’ [27].

For observational studies, the STrengthening the Reporting of OBservational studies in Epidemiology (STROBE) checklist was used to evaluate items related to the article´s title, abstract, introduction, methods, results and discussion sections and other information such as funding [28]. The Quality Assessment Tool (QAT) was used to assess the selection bias, study design, confounders, blinding, data collection methods, withdrawals and drop-outs, intervention integrity, and statistical analyses [29].

To validate the quality of case reports and case series, the Institute of Health Economics (IHE) Quality Appraisal Checklist for Case Series Studies (QACCSS) was used [30]. The evaluation was based on the study objective, study design, study population, intervention and co-intervention, outcome measures, statistical analysis, results, conclusions, competing interests, and sources of support.

An evaluation of the risk of bias was performed for all studies by IDF and MKÅ, except for the case reports. All such evaluations are presented in S2.1 –S2.5 Tables in S1 File. Disagreements were resolved first through discussions between IDF and MKÅ, and, if necessary, by consulting the clinicians SCC and SMC.

Subgroup analyses were performed for all studies included in meta-analysis. The categories for the subgroup analyses were: (1) HbA1c levels before starting CIPII treatment (≤ 7% and > 7%), (2) study type (case-control studies and crossover studies), (3) duration of the CIPII-period (≤ 6 months and > 6 months), and (4) whether or not there was an additional controlled CSII follow-up-period with subsequent CIPII-period. As an additional analysis, studies were sorted by the duration of the CIPII-period (months) to provide information about changes in the effect with time. All subgroup analyses are reported in S1 –S7c Figs in S1 File.

Evaluation of heterogeneity between studies was performed for studies reporting HbA1c levels by meta-regression and bubble plot analysis with 95% CI and linear prediction of HbA1c levels with CIPII treatment, using CSII controls for comparison.

Heterogeneity of effects was also explored with funnel plots [31] when ten or more studies were included in the meta-analysis. Assessments for publication bias across studies were performed using graphical (funnel plot) and statistical (Egger΄s test: random-effect model, t-distribution) analyses. For quantitative testing of skewness in the funnel plot, the Egger΄s test was chosen to analyse the MD of continuous outcomes.

A cumulative sequential meta-analysis of the studies was performed according to the duration of the CIPII-period.

## Results

### Literature selection

**Fig 1:**
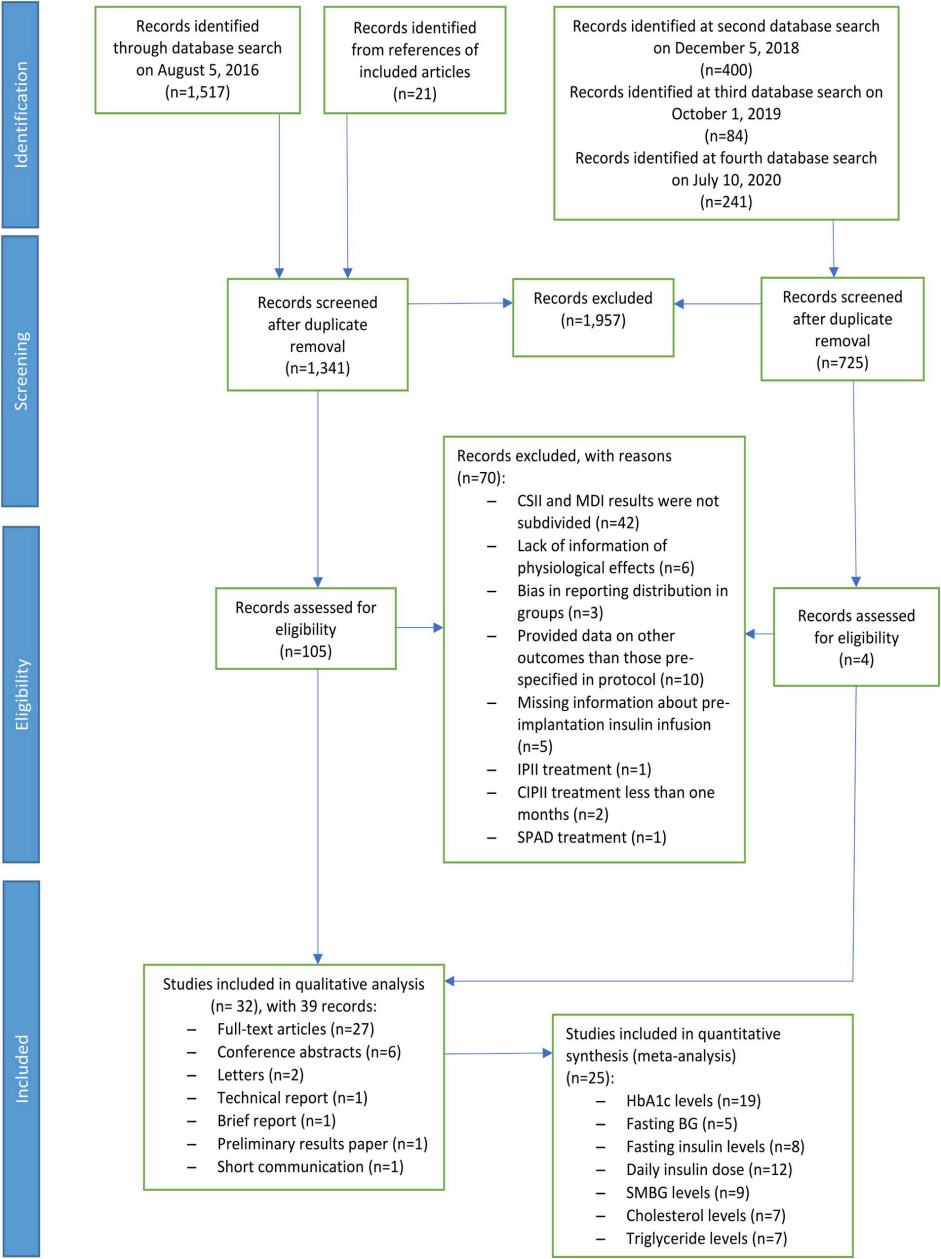
Flow chart of the screening and selection of included studies.

CSII, continuous subcutaneous insulin infusion; MDI, multiple daily injections; IPII, intraperitoneal insulin infusion; CIPII, continuous intraperitoneal insulin infusion; SPAD, subcutaneous peritoneal access device; BG, blood glucose; SMBG, self-monitoring blood glucose.

On the 10^th^ of July 2020, our literature searches identified 2,263 reports. After the abstract screening, 109 potentially eligible reports remained (Fig 1). After applying the additional exclusion criteria, 70 of the 109 reports were excluded. In total, 32 studies describing a total of 39 reports were included in the systematic review, including one full-text article in Italian [32] and one full-text article in German [33].

### Systematic review

Twenty-four [12, 13, 17, 18, 20–22, 32, 34–54] out of the 32 studies were cross-over studies with patients receiving at least three months of CSII treatment prior to 1.5–34 months of CIPII treatment. Only two of the studies were randomised studies [39, 55]. In the 30 studies that reported the sex of the participants, more men (n = 167; 55%) than women (n = 136; 45%) were included in the CIPII period. In these 30 studies, the participants’ ages ranged from 19 to 82 years (Table 1). In the ten studies [12, 13, 19, 22, 32, 33, 35, 38, 53, 56] that did report age separately for women and men, the mean age (range) was 35.1 (18–61) years in women and 38.9 (19–62) years in men. Ten out of the 32 studies were published in the 2000s; these included 122 participants during the CIPII period and 170 participants during the CSII period [23, 34–37, 45–49, 55–61].

**Table 1:** Characteristics of studies included in the systematic review.

Study	Study design	Number of Participants	Sex (Male or Female)	Age (mean±SD or range) (years)	HbA1c at inclusion (% or range)	CSII minimum period (month)	CIPII minimum period (month)
**Giacca et al. 1993 (France) [39]**	RCs	5	1/4	31–50	7.4	96 hours	3
**Liebl et al. 2009 (Multinational) [55]**	RFUs	CIPII: 15	CIPII: M:11/4	CIPII: 50.5	CIPII: 8.2	6	12
		CSII: 21	CSII: M: 9/12	CSII: 45.3	CSII:8.3		
**Micossi et al. 1986 (Italy) [13]**	NRCs	6	3/3	22–50	7.25	12	1 ½
**Beylot et al. 1987 (France) [12]**	NRCs	4	3/1	36–51	7.6 (5.0–9.2)	2	2
**Wredling, Adamson et al. 1991 (technical report) (Sweden) [53]**	NRCs	6	4/2	31–49	8.7 (7.0–9.5)	12	15
**Wredling, Liu et al. 1991 (Sweden) [54]**	NRCs	6	4/2	31–49	7.7–10.2	24	6.9
**Georgopoulos et al. 1992 (USA) [38]**	NRCs	7	5/2	19–40	9.83 (7.4–12.0)	ND	12
**Pitt et al. 1992 (USA) [18]**	NRCs	10	8/2	19–56	9.1	3	34^a^
**Renard et al. 1993 (France) [22]**	NRCs	8	6/2	31–53	ND	2.4	12
**Georgopoulos et al 1994 (USA) [17]**	NRCs	8	5/3	37±7	9.4	ND	6
**Lassmann-Vague et al. 1994 (short communication) (France) [44]**	NRCs	11	5/6	21–48	7.0	6	3
**Raccah et al. 1994 (letter) (France) [51]**	NRCs	11	6/5	21–48	6.9	3	10
**Schnell et al. 1994 (Germany) [52]**	NRCs	5	1/4	25–62	9.8	39	12
**Lassmann-Vague et al. 1995/1998 (article/letter) (France) [20, 21]**	NRCs	15	8/9	ND	ND	1	24
**Guerci et al. 1996 (France) [40]**	NRCs	14	9/5	40±6.2	6.1	14.2	4
**Hanaire-Broutin et al. 1996 (France) [41]**	NRCs	18	11/7	25–65	7.6	3	12
**Lassmann-Vague et al. 1996 (France) [43]**	NRCs	11	6/5	36.9±9	7.7	ND	2
**Pacifico et al. 1997 (Italy) [32]**	NRCs	8	5/4	18–50	6.5	3	12
**Oskarsson et al. 1999 (Sweden) [50]**	NRCs	7	5/2	36–50	8.5	6	11
**Oskarsson et al. 2000 (Sweden) [49]**	NRCs	7	5/2	36–50	8.6	12	11
**Duvillard et al. 2005/2007 (brief report/article) (France) [36, 37]**	NRCs	7	6/1	48±6.5	7.34	ND	3
**Liebl et al. 2013/2014 (c.p) (Germany) [45–48]**	NRCs	12	2/10	28–82	9.0	ND	12
**Dassau et al. 2017 (France) [35]**	NRCs	10	7/3	18–65	7.7	102	1
**Jeandidier et al. 1992 (preliminary results) (France) [42]**	Retro.Cs	8	ND	33.5±2.9	6.64	ND	10
**Catargi et al. 2002 (France) [34]**	Retro.Cs	14	5/9	50.6±12.8	7.8	1.5	3
**Jeandidier et al. 2002 (France) [57]**	NRFUs	CIPII: 13	CIPII: 6/7	CIPII: 36.8±1.7	CIPII: ND	6	6
		CSII: 11	CSII: 6/5		CSII: ND		
				CSII: 43.1±3.4			
**Van Dijk et al. 2016**	NRFUs	CIPII: 39	CIPII: 14/25	CIPII: 18–70	CIPII: 8.3	48	48
**Van Dijk et al. 2020 (The Netherlands) [23, 62]**		CSII: 74	CSII: 30/44	CSII: 48±12	CSII: 7.9		
**Colette et al. 1989 (France) [63]**	C-Cs	CIPII: 13	CIPII: ND	CIPII: 30±3	CIPII: 8.0	7	10
		CSII: 11	CSII: ND	CSII: 32±3	CSII: 8.9		
**Selam et al. 1989 (UK) [19]**	C-Cs	CIPII: 6	CIPII: 4/2	CIPII: 25–43	CIPII: 8.3	12	6
		CSII: 8	CSII: 5/3	CSII: 26–67	CSII: 8.7		
**Walter et al. 1989 (Germany) [33]**	C-Cs	CIPII: 6	CIPII: 6/0	CIPII:21–39	CIPII:8.0	6	3
		CSII: 6	CSII: 6/0	CSII:23–31	CSII:7.9		
**Hedman et al. 2009/2014; Arnqvist et al. 2010 (c.p/article; c.p) (Sweden) [58–60]**	C-Cs	CIPII: 10	CIPII: 5/5	CIPII: 53.1±9.1	CIPII: 8.6	6	6
		CSII:20	CSII:10/10		CSII:7.9		
				CSII:52.8±9.0			
**Catargi et al 2000 (case report) (France) [56]**	CR	1	1/0	32	ND	6	1.5

RCs, randomised crossover study; RFUs, randomised follow-up study; NRCs, non-randomised crossover study; Retro.Cs, retrospective crossover study; C-Cs, case-control study; NRFUs, non-randomised follow-up study; CR, case report; CIPII, continuous intraperitoneal insulin infusion; CSII, continuous subcutaneous insulin infusion; ND, no data available; c.p, conference poster; ^a^, available glycaemic control data for the first 18 months.

Twenty-eight studies originated from single European countries, three from the USA [17, 18, 38], and one study was a multinational study [55] (Table 1). All overviews and procedures are summarised in the S2.1 –S2.14 Tables in S1 File.

HbA1c values were reported in 19 studies that included a total of 178 participants in the CIPII-period versus 188 participants in the CSII-period.

### Glycaemic control

#### Meta-analysis: HbA1c

**Fig 2:**
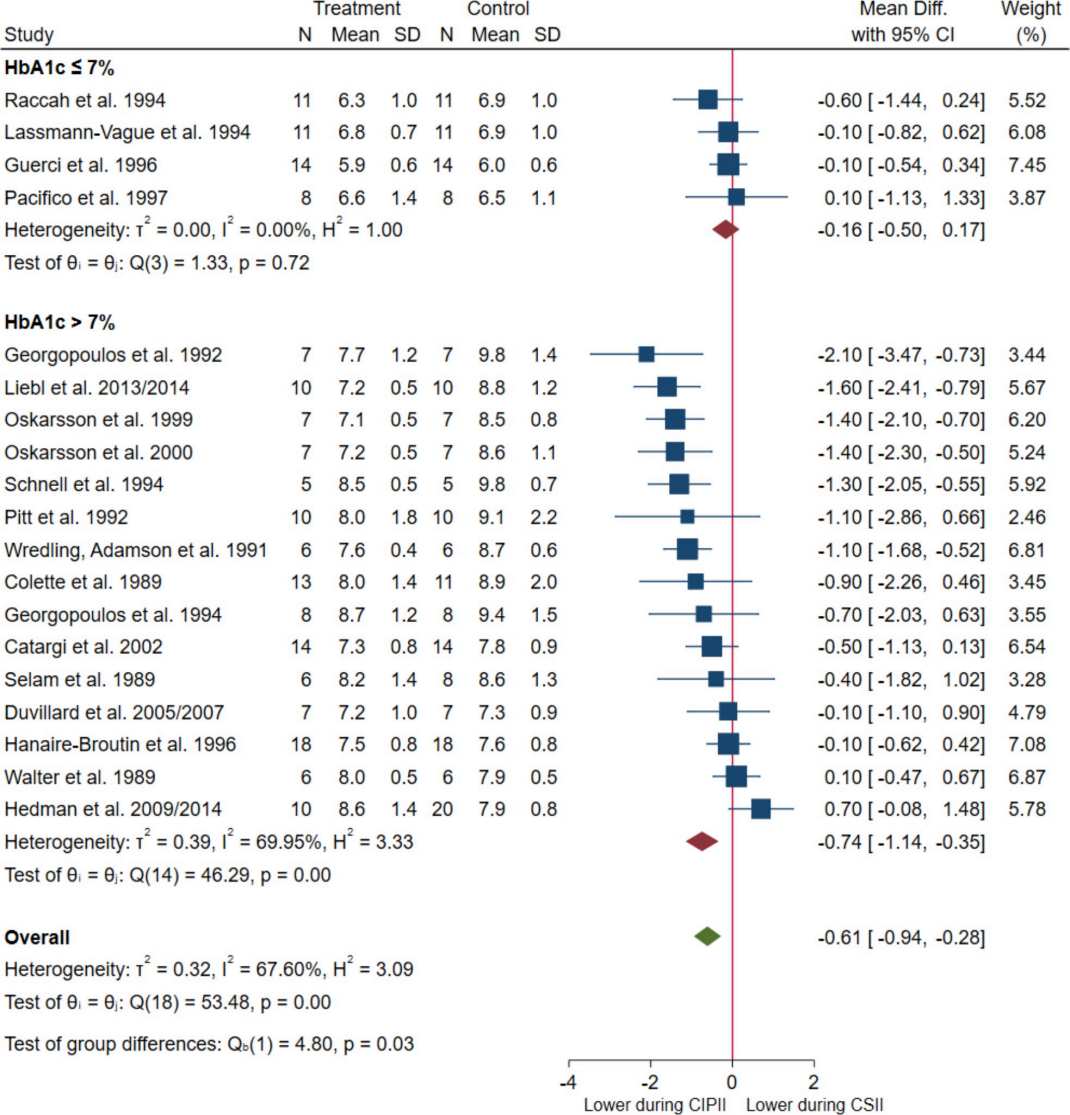
Meta-analysis of HbA1C (%) in patients during CIPII treatment compared to that during control treatment (CSII). Treatment, continuous intraperitoneal insulin infusion (CIPII); Control, continuous subcutaneous insulin infusion (CSII). Studies ordered by effect size (mean difference) and divided into subgroups: HBA1c levels ≤ 53.0 mmol/mol (≤ 7%) and HbA1c levels > 53.0 mmol/mol (> 7%) during control treatment (CSII).

**Fig 3:**
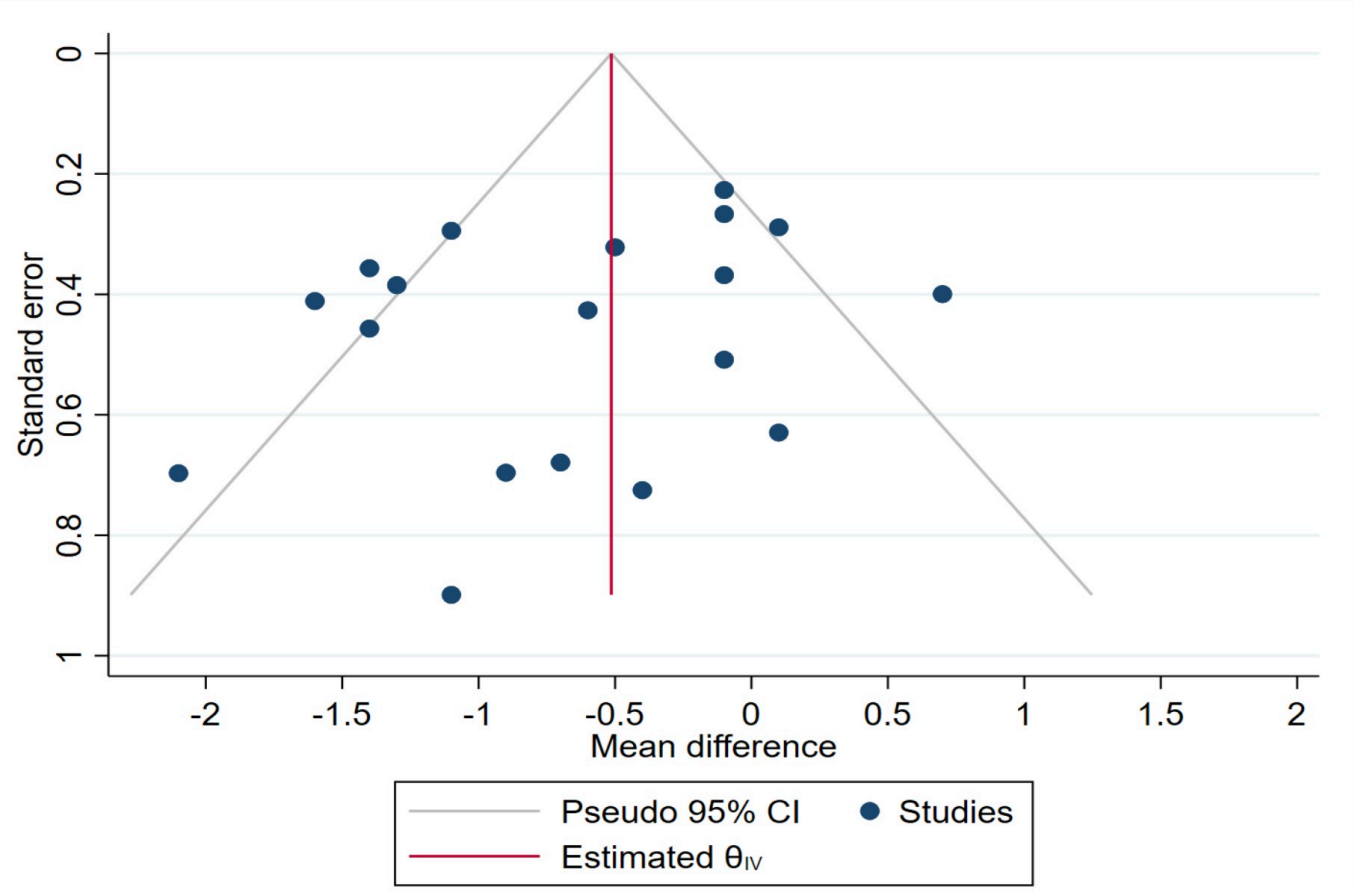
Funnel plot of HbA1c (%) during CIPII treatment compared to that during control treatment (CSII). The funnel plot includes diagonal lines representing expected distribution of studies in the absence of heterogeneity (95% of the studies should lie within these diagonal lines). The lines are not strict 95% confidence interval, therefore, referred as ‘pseudo 95% CI’.

When including all 19 studies (CIPII, n = 178; CSII, n = 188) [17–19, 32–34, 36–38, 40, 41, 44–53, 58–60, 63] in the random-effect meta-analysis, the HbA1c levels were significantly lower during CIPII treatment than during CSII treatment (MD = -6.7 mmol/mol, [95% CI: -10.3 –-3.1]; in percentage: MD = -0.61%, [95% CI: -0.94 –-0.28], p = 0.0002; Fig 2). While substantial heterogeneity was present (I^2^: 67.6%, p > 0.0001) and also evident in the funnel plot (Fig 3), the relative symmetry of the funnel plot was supported by a non-significant Egger΄s test result (p = 0.293).

*Subgroup analysis*. In subgroup analysis according to HbA1c levels before starting CIPII treatment, significantly lower HbA1c levels were observed during CIPII treatment than during CSII treatment in the subgroup with HbA1c levels > 53.0 mmol/mol (> 7%) and remained unchanged in the subgroup with HbA1c levels ≤ 53.0 mmol/mol (≤ 7%) (MD = -8.1 mmol/mol, [95% CI: -12.5 –-3.8], p < 0.01 and MD = -1.8 mmol/mol, [95% CI: -5.5–1.9], p = 0.33, respectively; in percentage: MD = -0.74%, [95% CI: -1.14 –-0.35], p < 0.01 and MD = -0.16%, [95% CI: -0.50–0.17], p = 0.33, respectively; S1c. A and S1d Fig in S1 File). The difference between the two subgroups was significant (p = 0.03). There was substantial heterogeneity between the studies with HbA1c levels > 53.0 mmol/mol (> 7%) (I^2^: 70%, p < 0.01) and no heterogeneity in the studies with HbA1c levels ≤ 53.0 mmol/mol (≤ 7%) (I^2^: 0%, p = 0.72).

In subgroup analysis according to study types, significantly lower HbA1c levels were observed during CIPII treatment than during CSII treatment in the crossover studies, while HbA1c levels remained unchanged in the case-control studies (MD -8.2 mmol/mol, [95% CI: -11.9 –-4.6], p < 0.01 and MD = 0.8 mmol/mol, [95% CI: -5.5–7.1], p = 0.8, respectively; in percentage: MD = -0.75%, [95% CI: -1.09 –-0.42], p < 0.01 and MD = 0.07%, [95% CI: -0.50–0.65], p = 0.8, respectively; S1c. B and S1d Fig in S1 File). The difference between the two subgroups was significant (p = 0.01). In both study type subgroups there was a substantial amount of heterogeneity (I^2^: 62%, p < 0.01 and I^2^: 34%, p = 0.19, respectively).

In subgroup analysis according to duration of the CIPII-period, significantly lower HbA1c levels were observed in the subgroup with a longer CIPII-period (> 6 months) while HbA1c levels remained unchanged in the subgroup with a shorter CIPII-period (≤ 6 months) (MD = -10.7 mmol/mol, [95% CI: -15.2 –-6.1], p < 0.01 and MD = -2.3 mmol/mol, [95% CI: -6.2–1.6], p = 0.25, respectively; in percentage: MD = -0.98%, [95% CI: -1.39 –-0.56], p < 0.01 and MD = -0.21%, [95% CI: -0.57–0.15], p = 0.25, respectively; S1c. C and S1d Fig S1 File). The difference between the two subgroups was significant (p = 0.01). In both subgroups of CIPII treatment duration there was substantial heterogeneity (I^2^: 56%, p = 0.01 and I^2^: 49%, p = 0.06, respectively).

In subgroup analysis according to whether or not there was an additional controlled CSII follow-up-period, significantly lower HbA1c levels were observed in both subgroups (MD = -6.7 mmol/mol, [95% CI: -11 –-2.3], p = 0.003 and MD = -7.0 mmol/mol, [95% CI: -12.7 –- 1.3], p = 0.015, respectively; in percentage: MD = -0.61%, [95% CI: -1.01 –-0.21], p = 0.003 and MD = -0.64%, [95% CI: -1.16 –-0.12], p = 0.015, respectively; S1c. D and S1d Fig in S1 File), with no significant difference between the subgroups (p = 0.93). There was substantial heterogeneity in both subgroups (I^2^: 72%, p < 0.01 and I^2^: 45%, p = 0.17, respectively).

*Meta-regression*. The regression coefficient for duration of the CIPII treatment was - 0.068 (p > 0.002). Thus, with every month of the CIPII treatment HbA1c decreased 0.7 mmol/mol (in percentage: 0.068%). The proportion of between-study variance explained by the *duration of CIPII-period* (R^2^) was 52%. The residual variation was due to substantial heterogeneity (I^2^: 49%, p = 0.014). In addition, a bubble plot of the observed effect size against the duration of CIPII-period overlaid with the predicted regression and confidence-interval lines, shows a similar pattern (S1e Fig in S1 File). This pattern was also observed in the subgroup meta-analysis with *duration of CIPII-period in months* (S1d Fig S1 File).

*Cumulative meta-analysis*. To evaluate the change in HbA1c levels with time during CIPII treatment compared to that during CSII treatment, a cumulative meta-analysis was performed (S1f Fig in S1 File). The results indicated that the HbA1c levels were progressively lower during CIPII treatment than during CSII treatment. The total difference became statistically significant (p < 0.05) after the inclusion of the study by Georgopoulos et al. [38] (MD = -5.3 mmol/mol, [95% CI: -9.7 –-0.8], p = 0.023; in percentage: MD = -0.48%, [95% CI: -0.89 –-0.07], p = 0.023), and the tendency of decrease in the MD during CIPII treatment, compared to that during CSII treatment, remained significant throughout the analysis (-5.1 to -5.7 mmol/mol, in percentage: -0.47 to -0.61%).

Neither of the two randomised studies [39, 55] was included in the meta-analysis to assess the effect of treatment on HbA1c levels, as the mean and SD or SEM values were not reported.

Detailed information about all studies and reported results pertaining to glycaemic control is available in S2.1 Table in S1 File.

#### Meta-analysis: Fasting blood glucose

**Fig 4:**
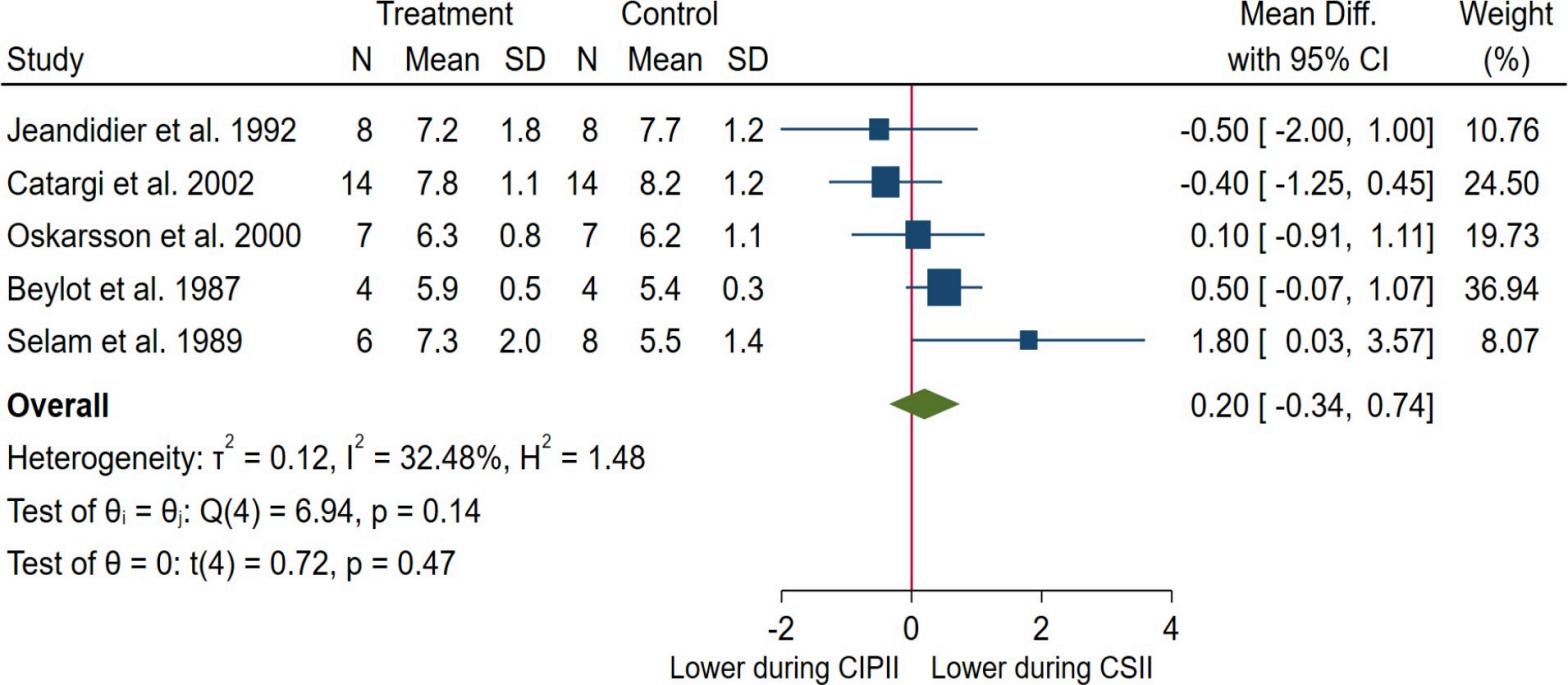
Meta-analysis of fasting blood glucose (mmol/L) in patients during CIPII treatment compared to that during control treatment (CSII). Treatment, continuous intraperitoneal insulin infusion (CIPII); Control, continuous subcutaneous insulin infusion (CSII). Studies are ordered by effect size (mean difference).

When including all five studies that reported fasting BG (CIPII, n = 39; CSII, n = 41) [12, 19, 34, 42, 49], the fasting BG levels remained unchanged during CIPII treatment compared to those during CSII treatment (MD = 0.20 mmol/L, [95% CI: -0.34–0.74], p = 0.47; in mg/dL: MD = 3.6 mg/dL, [95% CI: -6.1–13.3], p = 0.47; Fig 4). The heterogeneity between the studies was low (I^2^: 32%, p = 0.14).

*Subgroup analysis*. The mean difference in blood glucose levels was not different in any of the subgroups analysed (S2b Fig in S1 File).

The group difference in fasting BG levels remained unchanged during CIPII treatment compared to that during CSII treatment whether the HbA1c levels before starting CIPII were ≤ 53.0 mmol/mol (≤ 7%) or > 53.0 mmol/mol (> 7%) (p = 0.34); according to study type (case-control studies vs crossover studies) (p = 0.07); and whether or not there was an additional controlled CSII follow-up-period with subsequent CIPII-period (p = 0.74) (S2a and S2b Fig in S1 File). Subgroup analysis according to the duration of the CIPII-period could not be performed because all the included studies had a short CIPII-period (≤ 6 months).

#### Other primary outcomes: Hypoglycaemia

In total, ten studies reported outcomes related to hypoglycaemia. Out of these studies, seven reported on mild hypoglycaemia [13, 18, 35, 42, 49, 50, 55] and five reported on severe hypoglycaemia [18, 22, 32, 45–48, 55], most of which defined severe hypoglycaemia as cases requiring assistance (requiring hospitalisation or IV glucose administration, or events accompanied by unconsciousness or seizure). One randomised study observed a significantly reduced frequency of severe hypoglycaemia during the CIPII-period compared to that during the CSII-period (0.35 vs 0.86 events per patient-year, p = 0.013) [55]. The frequency of severe hypoglycaemic events was unchanged for the first three months of CIPII treatment, whereas it was reduced in the subsequent nine months (0.72 vs 0.15 events per patient-year, respectively, p-value not calculated) [55].

Three studies reported, respectively, zero [22], 0.43 [18], and 1.5 [45–48] severe hypoglycaemic events per patient-year during the CIPII-period versus 0.54 [22] and 12 [45–48] events per patient-year during the CSII-period. One study did not provide data for the CSII-period [18]. Among the two studies that reported on hypoglycaemic coma, no such events occurred during the CIPII-period [18, 22] compared to 0.54 events per patient-year during the CSII-period [22]. No other studies reported on hypoglycaemic coma during periods of CIPII or CSII. One study reported no difference in the occurrence of severe hypoglycaemia [32].

One prospective study that evaluated SMBG reported a reduced time spent in hypoglycaemia during the CIPII-period (SMBG < 3.9 mmol/L, p < 0.05), whereas the time spent in more pronounced hypoglycaemia (SMBG < 2.8 mmol/L) was similar between the two treatment periods [13]. However, four other studies observed no differences in the occurrence of hypoglycaemic events (SMBG < 3.0 mmol/L) in the last four weeks of the treatment periods [49, 50], in the frequencies of events per patient-year during those periods [55], or in the occurrence of BG levels < 3.8 mmol/L during a 24-hour period (based on a continuous glucose monitoring (CGM) profile) [35] (S2.1 and S2.8 Tables in S1 File).

#### Other primary outcomes: Hyperglycaemia

One prospective study that collected CGM data reported less time spent in hyperglycaemia (BG > 10.0 mmol/L, p < 0.05) during a 24-hour CIPII treatment compared to that during the CSII treatment [35], whereas another study that assessed SMBG observed no difference in hyperglycaemia (BG > 10.0 mmol/L) during a six-week period [13]. However, both studies reported a reduced amount of time spent in severe hyperglycaemia (BG > 14.0 mmol/L, p < 0.05) during the CIPII treatment compared to that during the CSII treatment (S2.1, S2.8 Tables in S1 File) [13, 35].

### Insulin levels

#### Meta-analysis: Fasting insulin levels

**Fig 5:**
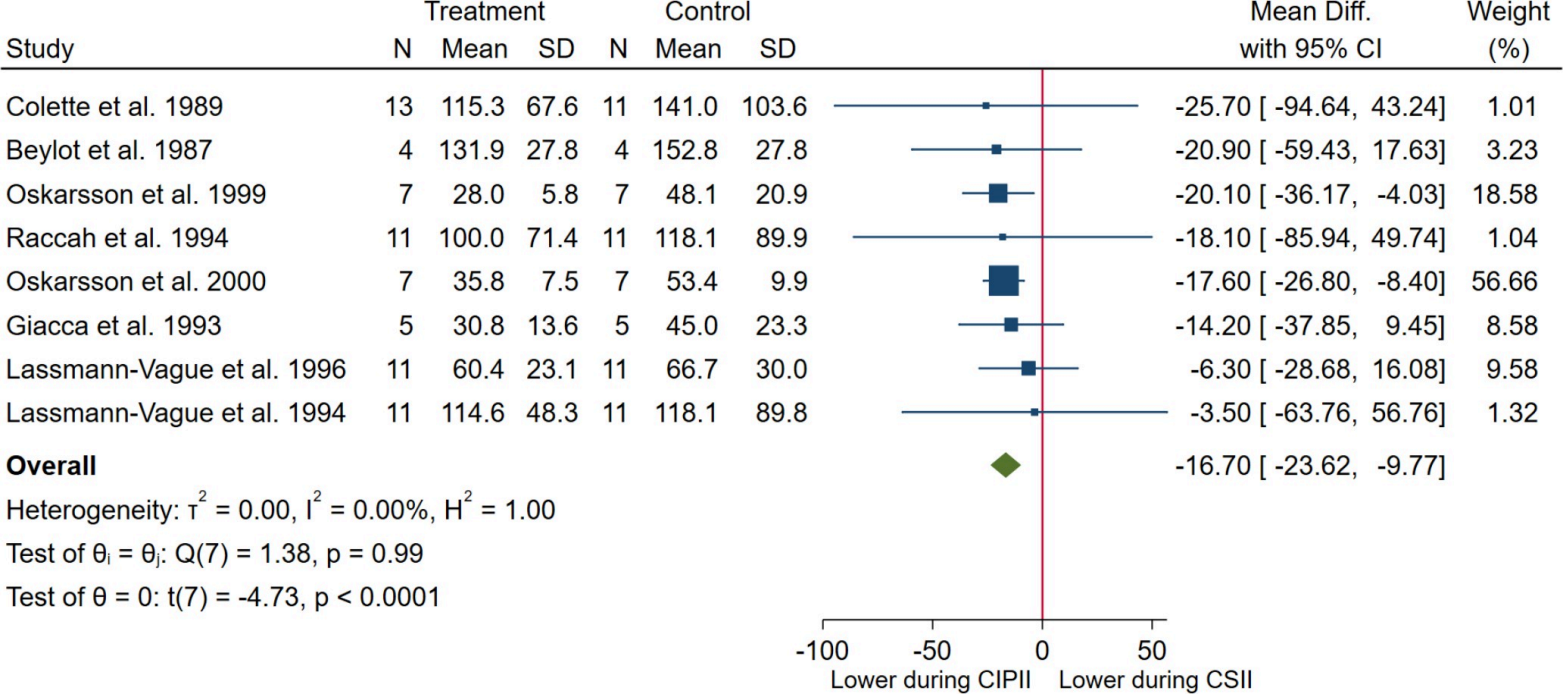
Meta-analysis of fasting insulin (pmol/L) in patients during CIPII treatment compared to that during control treatment (CSII). Treatment, continuous intraperitoneal insulin infusion (CIPII); Control, continuous subcutaneous insulin infusion (CSII). Studies ordered by effect size (mean difference).

When including all eight studies that reported fasting insulin levels (CIPII, n = 69; CSII, n = 67) [12, 39, 43, 44, 49–51, 63], the fasting insulin levels were significantly lower during CIPII treatment than during CSII treatment (MD = -16.70 pmol/L, [95% CI: -23.62 –-9.77], p < 0.0001; Fig 5). There was no heterogeneity between the studies (I^2^: 0%, p = 0.99).

*Subgroup analysis*. In subgroup analysis according to HbA1c levels before starting CIPII treatment, significantly lower fasting insulin levels were observed during CIPII treatment than during CSII treatment in the subgroup with HbA1c levels > 53.0 mmol/mol (> 7%), while fasting insulin levels remained unchanged in the subgroup with HbA1c levels ≤ 53.0 mmol/mol (≤ 7%) (MD = -16.86 pmol/L, [95% CI: -23.87 –-9.85], p < 0.001 and MD = -9.94 pmol/L, [95% CI: -54.99–35.11], p = 0.66, respectively; S3a. A and S3b Fig in S1 File). However, there was no difference between the subgroups (p = 0.77) and there was no heterogeneity in the subgroups (I^2^: 0%, p = 0.95 and I^2^: 0%, p = 0.75, respectively).

In subgroup analysis according to study types, significantly lower fasting insulin levels were observed during CIPII treatment than during CSII treatment in the crossover studies while levels remained unchanged in the case-control studies (MD = -16.61 pmol/L, [95% CI: -23.57 –-9.64], p < 0.001 and MD = -25.70 pmol/L, [95% CI: -94.64–43.24], p = 0.465, respectively; S3a. B and S3b Fig in S1 File). However, there was no statistical difference between the two groups (p = 0.80). There was no heterogeneity in both subgroups (I^2^: 0%, p = 0.97 and I^2^: 0%, p = not possible to calculate (n = 1), respectively).

In subgroup analysis according to duration of the CIPII-period, significantly lower fasting insulin levels were observed during CIPII treatment than during CSII treatment in both subgroups (MD = -15.20 pmol/L, [95% CI: -25.98 –-4.43], p = 0.006 and MD = -17.75 pmol/L, [95% CI: -26.79 –-8.71], p < 0.001, for CIPII-period ≤ 6 months and CIPII-period > 6 months, respectively; S3a. C and S3b Fig in S1 File) with no difference between the subgroups (p = 0.72). There was no heterogeneity in the subgroups (I^2^: 0%, p = 0.88 and I^2^: 0%, p = 0.97, respectively).

In subgroup analysis according to whether or not there was an additional controlled CSII follow-up-period, significantly lower fasting insulin levels were observed during CIPII treatment than during CSII treatment in the subgroup without controlled CSII follow-up-period while levels remained unchanged in the subgroup with controlled CSII follow-up-period (MD = -16.99 pmol/L, [95% CI: -24.42 –-9.56], p < 0.001 and MD = -14.77 pmol/L, [95% CI: -33.89–4.34], p = 0.13, respectively; S3a. D and S3b Fig in S1 File), with no difference between the subgroups (p = 0.83). There was no heterogeneity in the subgroups (I^2^: 0%, p = 0.89 and I^2^: 0%, p = 0.89, respectively).

#### Meta-analysis: Daily insulin dose

**Fig 6:**
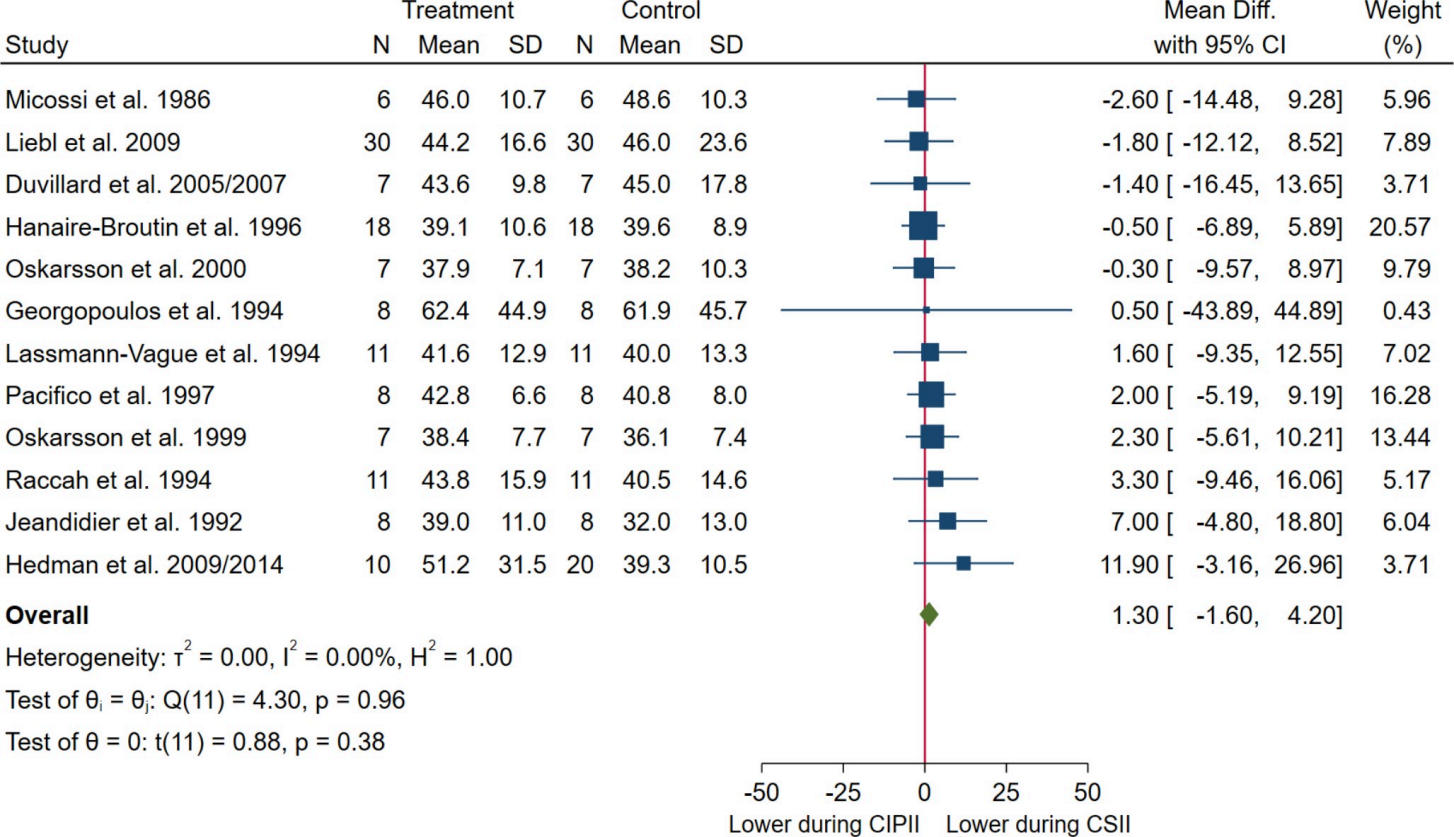
Meta-analysis of mean daily insulin (U/24 hours) in patients during CIPII treatment compared to that during control treatment (CSII). Treatment, continuous intraperitoneal insulin infusion (CIPII); Control, continuous subcutaneous insulin infusion (CSII). Studies ordered by effect size (mean difference).

**Fig 7:**
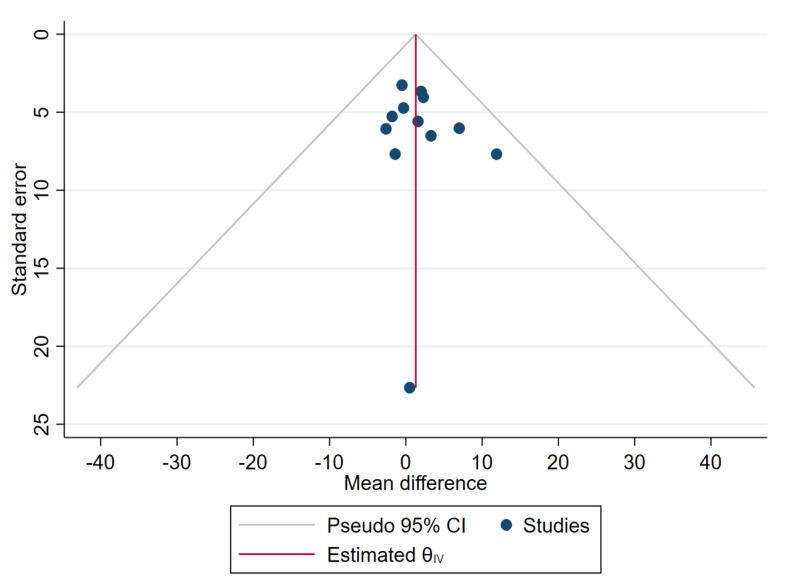
Funnel plot of daily insulin dose (U/24 hours) during CIPII treatment compared to that during control treatment (CSII). The funnel plot includes diagonal lines representing expected distribution of studies in the absence of heterogeneity (95% of the studies should lie within these diagonal lines). The lines are not strict 95% confidence interval, therefore, referred as ‘pseudo 95% CI’.

When including all 12 studies that reported daily insulin dose (CIPII, n = 131; CSII, n = 141) [13, 17, 32, 36, 37, 41, 42, 44, 49–51, 55, 58–60], the daily insulin dose remained unchanged during CIPII treatment compared to that during CSII treatment (MD = 1.30 U/24 hours, [95% CI: -1.60–4.20], p = 0.38; Fig 6), with no heterogeneity between the studies (I^2^: 0%, p = 0.96). Homogeneity was also evident in the funnel plot (Fig 7). The relative symmetry of the funnel plot is supported by the non-significant Egger’s test result (p = 0.621).

*Subgroup analysis*. The MD in daily insulin dose was not different in any of the subgroups analysed (S4b Fig in S1 File).

The group difference in daily insulin dose remained unchanged during CIPII treatment compared to that during CSII treatment, irrespective of whether the HbA1c levels before starting CIPII treatment were ≤ 53.0 mmol/mol (≤ 7%) or > 53.0 mmol/mol (> 7%) (p = 0.41); according to study type (case-control studies vs crossover studies) (p = 0.69); according to the duration of the CIPII-period (p = 0.71); and irrespective of whether or not there was an additional controlled CSII follow-up-period with a subsequent CIPII-period (p = 0.96) (S4a and S4b Fig in S1 File).

#### Other primary outcomes: Time to reach peak insulin concentrations

Three studies, all using regular human insulin, reported post-bolus systemic insulin levels at specific time-points (after 30 and 60 minutes for IP administration). All three studies observed earlier maximum insulin levels during the CIPII treatment compared to the CSII treatment (60 vs 133.6 minutes (p < 0.006) [54]; 60 vs 180 minutes (p < 0.05) [43]; and 30 minutes vs 60 minutes (p-value not reported) [19]).

#### Other primary outcomes: Maximum insulin levels

Two studies reported higher maximum insulin levels during the CIPII treatment than during the CSII treatment (179.18 vs 125.01 pmol/L, respectively (p < 0.05) [43] and 263.91 vs 145.84 pmol/L, respectively (30 minutes after bolus administration, p < 0.05) [19]. Another study reported no difference between the treatments 30 minutes after administering an insulin bolus [49].

#### Other primary outcomes: Time until insulin levels returned to basal levels

After the administration of a pre-breakfast insulin bolus, two studies observed that the insulin levels returned to baseline values after three hours during the CIPII treatment [19, 43] whereas during the CSII treatment, insulin levels either returned to baseline values after four hours [19] or remained elevated after five-and-a-half hours [43].

### Secondary outcomes

#### Meta-analysis: SMBG

When including all nine studies that reported SMBG levels (CIPII, n = 85; CSII, n = 85) [12, 13, 17, 18, 34, 38, 40, 44, 51], the SMBG levels were significantly lower during CIPII treatment than during CSII treatment (MD = -0.62 mmol/L, [95% CI: -1.01 –-0.23], p = 0.002; in mg/dL: MD = -11.2 mg/dL, [95% CI: -18.2 –-4.1], p = 0.002; S5a Fig in S1 File). However, there was moderate heterogeneity (I^2^: 42%, p = 0.05).

*Subgroup analysis*. In subgroup analysis according to HbA1c levels before starting CIPII treatment, significantly lower SMBG levels were observed during CIPII treatment than during CSII treatment in the subgroup with HbA1c levels > 53.0 mmol/mol (> 7%) while SMBG levels remained unchanged in the subgroup with HbA1c levels ≤ 53.0 mmol/mol (≤ 7%) (MD = -0.88 mmol/L, [95% CI: -1.34 –-0.42], p < 0.001 and MD = -0.19 mmol/L, [95% CI: -0.59–0.21], p = 0.345, respectively; in mg/dL: MD = -15.8 mg/dL, [95% CI: -24.1 –-7.6], p < 0.001 and MD = -3.4 mg/dL [95% CI: -10.6–3.8], p = 0.345, respectively; S5b. A and S5c Fig in S1 File). There was a significant difference between the subgroups (p = 0.03). There was a low heterogeneity between the studies with HbA1c levels > 53.0 mmol/mol (> 7%) (I^2^: 29%, p = 0.14) and no heterogeneity in the studies with HbA1c levels ≤ 53.0 mmol/mol (≤ 7%) (0%, p = 0.94).

A subgroup analysis according to study types was not possible to calculate as all the studies were crossover studies (S5b. B and S5c Fig in S1 File).

In subgroup analysis according to duration of the CIPII-period, significantly lower SMBG levels were observed during CIPII treatment than during CSII treatment in the subgroup with longer CIPII-period (> 6 months) while levels remained unchanged in the subgroup with shorter CIPII-period (≤ 6 months) (MD = -1.24 mmol/L, [95% CI: -2.40 –-0.07], p = 0.037 and MD = -0.30 mmol/L, [95% CI: -0.63–0.03], p = 0.074, respectively; in mg/dL: MD = -22.3 mg/dL, [95% CI: -43.2 –-1.3], p = 0.037 and MD = -5.4 mg/dL, [95% CI: -11.3–0.5], p = 0.074, respectively; S5b. C and S5c Fig in S1 File). The difference between the subgroups was not significant (p = 0.13). There was a substantial heterogeneity in both subgroups (I^2^: 56%, p = 0.01 and 49%, p = 0.06, respectively).

In subgroup analysis according to whether or not there was an additional controlled CSII follow-up-period, significantly lower SMBG levels were observed during CIPII treatment than during CSII treatment in the subgroup with controlled CSII follow-up-period, while levels remained unchanged in the subgroup without controlled CSII follow-up-period (MD = -0.72 mmol/L, [95% CI: -1.20 –-0.23], p = 0.004 and MD = -0.70 mmol/L, [95% CI: -1.62–0.22], p = 0.138, respectively; in mg/dL: MD = -13.0 mg/dL, [95% CI: -21.6 –-4.1], p = 0.004 and MD = -22.6 mg/dL, [95% CI: -29.2–4.0], p = 0.138, respectively; S5b. D and S5c Fig in S1 File). There was no significant difference between the subgroups (p = 0.97). There was non-essential heterogeneity in the subgroup with controlled CSII follow-up-period (I^2^: 29%, p = 0.34) and a substantial heterogeneity in the subgroup without controlled CSII follow-up-period (I^2^: 70%, p = 0.04).

#### Meta-analysis: Cholesterol

When including all seven studies that reported cholesterol levels (CIPII, n = 61; CSII, n = 61) [13, 17, 32, 36–38, 40, 51], the cholesterol levels remained unchanged during CIPII treatment compared to those during CSII treatment (MD = -0.06 mmol/L, [95% CI: -0.35–0.22], p = 0.67; S6a Fig in S1 File). There was no heterogeneity between the studies (I^2^: 0%, p = 0.81).

*Subgroup analysis*. The MD in cholesterol levels was not different in any of the subgroups analysed (S6c Fig in S1 File).

The group difference in cholesterol levels remained unchanged during CIPII treatment compared to that during CSII treatment whether the HbA1c levels before starting the CIPII treatment were ≤ 53.0 mmol/mol (≤ 7%) or > 53.0 mmol/mol (> 7%) (p = 0.52); according to length of the CIPII-period (p = 0.89); and whether or not there was an additional controlled CSII follow-up-period with subsequent CIPII-period (p = 0.20) (S6b and S6c Fig in S1 File). Subgroup analysis according to study type could not be performed because all the included studies were crossover studies.

#### Meta-analysis: Triglycerides

When including all seven studies that reported triglyceride levels (CIPII, n = 61; CSII, n = 61) [13, 17, 32, 36–38, 40, 51], the triglyceride levels remained unchanged during CIPII treatment compared to those during CSII treatment (MD = 0.09 mmol/L, [95% CI: -0.03–0.22], p = 0.15; S7a Fig in S1 File). There was non-essential heterogeneity between the studies (I^2^: 17%, p = 0.17).

*Subgroup analysis*. The MD in triglyceride levels was significantly different only in the subgroups according to whether or not there was an additional controlled CSII follow-up-period. Significantly higher triglyceride levels were observed in the subgroup with controlled CSII follow-up-period while levels remained unchanged in the subgroup without controlled CSII follow-up-period (MD = 0.60 mmol/L, [95% CI: 0.20–1.00], p = 0.003 and MD = 0.04 mmol/L, [95% CI: -0.07–0.16], p = 0.455, respectively; S7b. D and S7c Fig in S1 File). There was a significant difference between the subgroups (p = 0.01). There was no heterogeneity in the subgroups without a controlled CSII follow-up-period (I^2^: 0%, p = 0.80), while in the other subgroup, heterogeneity could not be calculated as only one study was included.

The group difference in triglyceride levels remained unchanged during CIPII treatment compared to that during CSII treatment, irrespective of whether the HbA1c levels before starting the CIPII treatment were ≤ 53.0 mmol/mol (≤ 7%) or > 53.0 mmol/mol (> 7%) (p = 0.44; S7b. A and S7c Fig in S1 File). There was a substantial heterogeneity in the subgroup with HbA1c levels > 53.0 mmol/mol (> 7%) (I^2^: 66%, p = 0.04) and no heterogeneity in the subgroup with HbA1c levels ≤ 53.0 mmol/mol (≤ 7%) (I^2^: 0%, p = 0.86; S7b. A Fig in S1 File).

Subgroup analysis according to the study type could not be performed since all the included studies were crossover studies (S7b. B and S7c Fig in S1 File).

The group difference in triglyceride levels remained unchanged during CIPII treatment compared to that during CSII treatment in the subgroup according to duration of the CIPII-period (p = 0.27). There was a substantial heterogeneity between the studies in the subgroup with CIPII-period ≤ 6 months (I^2^: 58%, p = 0.07), and no heterogeneity between the studies in the subgroup with CIPII-period > 6 months (I^2^: 0%, p = 0.70; S7b. C Fig in S1 File).

#### Other secondary outcomes

Analyses for the other secondary outcomes (levels of free fatty acids, lactate, ketone bodies, apolipoproteins, glucagon, adrenaline, noradrenaline, growth hormone, insulin-like growth factor, insulin-like growth factor binding proteins, sex hormone binding globulin, anti-insulin antibodies, and plasminogen activator inhibitor 1) are available in S2.1 –S2.14 Tables in S1 File.

#### Other secondary outcomes: Technical and medical complications

Technical and medical complications during the CIPII treatment (including inflammation, severe abdominal pain, severe insulin underdelivery, erythema, pump re-implantation, change of catheter, and insulin pump technical problems) are summarised in S2.6 Table in S1 File. Due to missing comparisons with the CSII treatment, these data were not evaluated in the main article.

## Discussion

This qualitative analysis and meta-analysis strongly indicated that improved glucose control can be achieved by switching from CSII treatment to CIPII treatment in patients with DM1. This included improved overall glucose control, as evaluated by HbA1c levels, as well as a reduced frequency of severe hyperglycaemia and severe hypoglycaemia. However, despite highly significant differences, the effect of the CIPII treatment was not overwhelmingly large, as it resulted in a reduction of HbA1c levels by only 6.7 mmol/mol (0.61%). Meta-regression analysis showed that the linear prediction of HbA1c levels decreased over time during CIPII treatment. This trend, which was also observed in the cumulative meta-analysis, increases the evidence that CIPII treatment lowers HbA1c levels during the treatment period and is not an ʻinclusion effectʼ or ʻstudy effectʼ mentioned previously [13, 18]. Subgroup analysis did not find a source of substantial heterogeneity. Furthermore, the funnel plot symmetry and non-significant Eggerʼs test (Fig 3) supported the conclusion that publication bias did not influence the results.

In the current systematic review and meta-analysis, subgroup analyses according to HbA1c levels before starting CIPII treatment, study type, duration of the CIPII-period, and whether or not there was a controlled CSII follow-up-period with a subsequent CIPII-period, were also performed.

These subgroup analyses revealed that a larger decrease in HbA1c levels was observed in the subgroup with HbA1c levels > 53.0 mmol/mol (> 7%) before starting CIPII treatment and in the crossover studies with CIPII treatment longer than six months compared to that in the meta-analysis for HbA1c levels with all studies. Additionally, in subgroups according to whether or not there was a controlled CSII follow-up period, the MD of the HbA1c levels was unchanged. Heterogeneity between studies was higher in the first three aforementioned subgroups (according to HbA1c levels before starting CIPII treatment, study type, duration of the CIPII period) with a higher MD.

The effect of decreased fasting insulin levels appeared to be related to the HbA1c levels before CIPII treatment and study type, as a significant difference appeared in the subgroup with HbA1c levels ≤ 53 mmol/mol (≤ 7%) in the crossover studies. However, fasting insulin levels decreased in the first six months and continued to decrease throughout CIPII treatment.

A similar pattern was observed in the SMBG levels in the subgroup with HbA1c levels ≤ 53 mmol/mol (≤ 7%) in the crossover studies. However, compared to that in the previous subgroup, significant difference appeared only in the subgroup with a CIPII treatment duration > 6 months.

Other metabolic variables included in the meta-analysis did not change by switching from CSII treatment to CIPII treatment.

### Implications for clinical practice

Although the reduction in HbA1c levels by CIPII treatment is limited, the fact that such an improvement can be achieved simply by switching the site of insulin delivery is noteworthy, especially as the total daily insulin dose remains unchanged, combined with a reduction in the frequency of severe hypoglycaemia.

The latter observation seems even more robust, as when the frequency of severe hypoglycaemia decreased during optimized treatment with CSII, a further decrease was observed during treatment with CIPII [55]. However, in four studies that reported outcome related to severe hypoglycaemia [18, 22, 32, 45–48], the raw data were presented without any statistical comparisons. Therefore, it remains uncertain whether CIPII improves the rate of overall hypoglycaemia, as only one study reported an overall decrease [13].

The fasting insulin levels were reduced by 16.70 pmol/L during CIPII treatment. More importantly, the post-bolus circulating insulin levels peaked earlier and returned to baseline levels faster during CIPII treatment than during CSII treatment. The reduced frequency of severe hyper- or hypoglycaemias is probably due to insulin concentrations peaking faster and returning to baseline levels more quickly after the administration of insulin boluses during CIPII treatment. Concurrently, glucose control inferred from HbA1c levels improved during CIPII treatment [13, 40, 42, 49, 50]. This probably reflects the fact that CIPII mimics the physiological, endogenous insulin profile more closely than does CSII treatment.

The difference we observed in the study’s main outcome measures could translate into long-term health benefits in those treated with CIPII compared to those receiving CSII treatment, even in the absence of clinically and significantly improved glucose control, and with the same overall insulin requirements. For instance, it should be noted that the incidence of myocardial infarction is increased 10-fold in young and middle-aged patients with DM1 compared to that in those in the general population without DM1 [64], and that circulating insulin levels have been linked to the pathogenesis of cardiovascular diseases [65]. Thus, reducing systemic hyperinsulinemia by switching to IP insulin delivery may, in the long-term, translate into a reduced prevalence of cardiovascular diseases in patients with DM1.

### Development of an artificial pancreas

Another possible benefit of IP insulin delivery could be the potential to improve the performance of an AP. At present, only SC insulin-hybrid APs are available; with these set-ups, patients have to inform the system of the amount of carbohydrates ingested, from which the system calculates the SC bolus of insulin to be administered. The development of a fully closed-loop AP requiring no regular daily intervention by the patient and at the same time maintaining glucose levels in the normal or close-to-normal range remains a distant dream; however, a switch from SC to IP insulin delivery could help such a dream to come true [6].

### Comparison with previous systematic reviews

As our focus was on the potential metabolic effects of IP versus. SC insulin delivery *per se*, we limited our systematic review only to studies of patients with DM1 that compared CIPII to CSII. Thus, we excluded MDI, as it implies the use of medium- and long-acting insulin formulations, which could influence the metabolic effects to a different extent. By focusing only on a comparison of CIPII and CSII, we limited the investigation exclusively to the use of continuously infused short-acting insulins.

In the past, two systematic reviews have been published comparing IP and SC insulin administration [66, 67]. In one of these, Almalki et al. compared IP to SC insulin delivery in patients with peritoneal dialysis, whereas Spaan et al. included a mixed group of patients with diabetes mellitus type 2 and DM1, in addition to trying to compare the effects of CIPII to MDI or CSII. Thus, neither of these existing reviews could provide useful information about the effects of IP versus SC insulin delivery *per se* in patients with DM1, specifically. Interestingly, and probably as a consequence of the study objectives, none of the studies included in our own systematic review were included in those two previous systematic reviews [66, 67]. Although nine out of the 13 studies that were included by Spaan et al. were identified in our first screening, they were subsequently excluded after applying the additional exclusion criteria.

In the current meta-analysis, additional subgroup analyses were performed, including those according to fasting BG levels, fasting insulin levels, daily insulin dose, SMBG levels, cholesterol levels, and triglyceride levels. However, in the main article we present data that show significant differences between CIPII treatment and CSII treatment. All meta-analyses and extracted qualitative data and analysis are available in the online supporting material.

### Strengths and limitations

As we included only studies that applied continuous insulin infusion, we minimised the potential confounding effects of other factors, increasing the likelihood that the difference in the site of insulin delivery was the main reason for the different effects we observed. This strict approach adds to the quality of systematic reviews and meta-analyses.

It should be noted that the majority of the articles included were published during the 1990s, and only ten studies were published after the year 2000. This limitation results in a relative lack of reports of patients treated with current pump technologies, CGM, and newer and faster-acting insulin formulations. Another limitation is that the lengths of the treatment periods differed between studies (ranging from 2–48 months for the CIPII periods), probably contributing to the high heterogeneity observed between studies. In addition, the majority of the reports were not purely prospective, but were rather small cohorts assembled during consecutive periods of clinical use of CIPII, with the data from the control period (CSII) often being less well-described. Sadly, this, combined with the limited number of studies reporting on many of the outcomes, limits the scientific robustness of many of our observations. This is particularly true for the secondary outcomes reported in the S1 File.

In general, the poor descriptions of the CSII treatment periods and the open design of all the studies increases the possibility of substantial ʻinclusion benefitʼ or ʻstudy effectʼ [18]. In all of the studies that reported HbA1c levels, only one avoided this effect by maintaining the same follow-up procedure during both treatment periods [13]. However, no improvement in glycaemic control was observed during one year of intense medical surveillance in the CSII-period. Unfortunately, the follow-up period in this study was only six weeks for each of the treatments. Thus, the HbA1c results in this study are likely to be less trustworthy than those of the other outcome measures.

## Conclusions

This meta-analysis suggests that CIPII treatment is superior to CSII treatment in improving glucose control in patients with DM1 who have poor glycaemic control. The effect is observed as a reduction in HbA1c levels and in reduced frequencies of severe hyperglycaemic and severe hypoglycaemic events. CIPII decreases circulating insulin levels and results in a more physiological insulin profile post bolus administration.

Thus, we hold that CIPII could be beneficial in terms of improved glucose control and more dynamic insulin effect on glucose levels. In the future, further work is needed to strengthen the evidence of the benefits of the use of CIPII.

## Supporting information

S1 Checklist (DOC)

S1 File (PDF)
