# Supplementary material for: Physiological effects of intraperitoneal versus subcutaneous insulin infusion in patients with diabetes mellitus type 1: A systematic review and meta-analysis
Source: PLoS One. 2021 Apr 13;16(4):e0249611. doi: 10.1371/journal.pone.0249611 (PMC8043377; doi:10.1371/journal.pone.0249611)
Supplement: S1 File — (PDF) [file pone.0249611.s002.pdf]

Supporting information

Physiologic effects of intraperitoneal versus subcutaneous insulin delivery in patients with diabetes mellitus type 1:

A systematic review

Ilze Dirnena-Fusini et al. 2020

Contents

Literature search strategy ... 4
Changes in the systematic review compared to the Protocol ... 5
Primary outcomes ... 5
Secondary outcomes ... 6
Extended information not described in the results ... 6
Excluded articles and reasons for exclusion ... 6
Risk of biases ... 7
Results of the search ... 9
Data extraction and quality assessment ... 9
Qualitative data analysis ... 9
Primary outcome: Glycaemic control ... 9
Primary outcome: Hypo-/ hyperglycaemia ... 11
Primary outcome: Insulin levels ... 12
Secondary outcomes: Intermediate metabolites ... 14
Secondary outcomes: counterregulatory hormones ... 15
Secondary outcome: Other metabolic outcomes ... 15
Secondary outcome: Complications ... 16
How to read the tables ... 16
Table S2.1. Intervention studies: Participant characteristics, description, outcomes: glycaemic control ... 18
Table S2.2. Intervention studies, Participant characteristics, description, outcomes: Insulin levels ... 25
Table S2.3. Intervention studies, Participant characteristics, description, outcomes: Intermediate metabolites ... 32
Table S2.4. Intervention studies, Participant characteristics, description, outcomes: Counterregulatory hormones ... 36
Table S2.5. Intervention studies, Participant characteristics, description, outcomes: Other outcomes ... 38
Table S2.6. Technical and physiological complications with intraperitoneal insulin pump and its attached system ... 41
Table S2.7. Methodological aspects of the included studies. ... 42
Table S2.8. Glycaemic control during the CIPII-period: Hypoglycaemia, normoglycaemia and hyperglycaemia events and/or time spent in ... 46
Table S2.9. Data modification for STATA: HbA1c. ... 47
Table S2.10. Data modification for STATA: SMBG. ... 48
Table S2.11. Data modification for STATA: Insulin levels. ... 49
Table S2.12. Data modification for STATA: cholesterol levels. ... 49
Table S2.13. Data modification for STATA: triglyceride levels. ... 50
Table S2.14. Data modification for STATA: insulin requirement ... 51

Figure S1a. Meta-analysis of HbA1c (%) in patients during CIPII treatment compared to that during control treatment (CSII). ... 52
Figure S1b. Subgroup meta-analysis of HbA1c (%) according to duration in patients during CIPII treatment compared to that during control treatment (CSII). ... 53
Figure S1c. Subgroup meta-analysis of HbA1c (%) in patients during CIPII treatment compared to that during control treatment (CSII). ... 54
Figure S1d. Overall subgroup meta-analysis of HbA1c (%) in patients during CIPII treatment compared to that during control treatment (CSII). ... 55
Figure S1e. Meta-regression analysis bubble-plot of HbA1c (%) in patients during CIPII treatment compared to that during control treatment (CSII). ... 56
Figure S1f. Cumulative meta-analysis of HbA1c (%) in patients during CIPII treatment compared to that during control treatment (CSII) according to duration of CIPII treatment. ... 57
Figure S2a. Subgroup meta-analysis of fasting blood glucose (mmol/L) in patients during CIPII treatment compared to that during control treatment (CSII). ... 58
Figure S2b. Summarised subgroup meta-analysis of fasting blood glucose (mmol/L) in patients during CIPII treatment compared to that during control treatment (CSII). ... 59
Figure S3a. Subgroup meta-analysis of fasting insulin (pmol/L in patients during CIPII treatment compared to that during control treatment (CSII). ... 60
Figure S3b. Summarised subgroup meta-analysis of fasting insulin (pmol/L) in patients during CIPII treatment compared to that during control treatment (CSII). ... 61
Figure S4a. Subgroup meta-analysis of daily insulin dose (U/24 hours) in patients during CIPII treatment compared to that during control treatment (CSII). ... 62
Figure S4b. Summarised subgroup meta-analysis of daily insulin dose (U/24 hours) in patients during CIPII treatment compared to that during control treatment (CSII). ... 63
Figure S5a. Meta-analysis of SMBG (mmol/L) in patients during CIPII treatment compared to that during control treatment (CSII). ... 64
Figure S5b. Subgroup meta-analysis of SMBG (mmol/L) in patients during CIPII treatment compared to that during control treatment (CSII). ... 65
Figure S5c. Summarised subgroup meta-analysis of SMBG (mmol/L) in patients during CIPII treatment compared to that during control treatment (CSII). ... 66
Figure S6a. Meta-analysis of cholesterol (mmol/L) in patients during CIPII treatment compared to that during control treatment (CSII). ... 67
Figure S6b. Subgroup meta-analysis of cholesterol (mmol/L) in patients during CIPII treatment compared to that during control treatment (CSII). ... 68
Figure S6c. Summarised subgroup meta-analysis of cholesterol (mmol/L) in patients during CIPII treatment compared to that during control treatment (CSII). ... 69
Figure S7a. Meta-analysis of triglycerides (mmol/L) in patients during CIPII treatment compared to that during control treatment (CSII). ... 70
Figure S7b. Subgroup meta-analysis of triglycerides (mmol/L) in patients during CIPII treatment compared to that during control treatment (CSII). ... 71

Figure S7c. Summarised subgroup meta-analysis of triglycerides (mmol/L) in patients during CIPII treatment compared to that during control treatment (CSII). 72

Data for Egger`s test from STATA 73

References 74

Literature search strategy

Table S1. Literature search strategy.

**Embase**		**PubMed**		**Scopus**		**Central**	
1	exp diabetes mellitus/	1	Diabetes mellitus[mh]	1	TITLE-ABS-KEY (diabet*)	1	Diabet*:ti,ab,kw
2	diabet*.ti,ab,kw.	2	diabet*[tiab] OR diabet*[ot]	2	TITLE-ABS-KEY (insulin resistan*)	2	insulin resistan*:ti,ab,kw
3	insulin resistan*.ti,ab,kw.	3	insulin resistan*[tiab] OR insulin resistan*[ot]	3	TITLE-ABS-KEY (impaired glucose tolerance)	3	impaired glucose tolerance:ti,ab,kw
4	impaired glucose tolerance.ti,ab,kw.	4	impaired glucose tolerance [tiab] OR impaired glucose tolerance [ot]	4	TITLE-ABS-KEY (Wolfram syndrome)	4	Wolfram syndrome:ti,ab,kw
5	Wolfram syndrome.ti,ab,kw.	5	Wolfram syndrome [tiab] OR Wolfram syndrome [ot]	5	#1 OR #2 OR #3 OR #4	5	#1 or #2 or #3 or #4
6	1 or 2 or 3 or 4 or 5	6	#1 OR #2 OR #3 OR #4 OR #5	6	TITLE-ABS-KEY (peritoneum)	6	intraperitone*:ti,ab,kw
7	exp peritoneum/	7	Peritoneum [mh]	7	TITLE-ABS-KEY (intraperitoneal)	7	peritone*:ti,ab,kw
8	exp intraperitoneal drug administration/	8	peritoneum[tiab] OR peritoneum[ot]	8	TITLE-ABS-KEY (peritoneal cavity)	8	#6 or #7
9	exp peritoneal cavity/	9	intraperitoneal [tiab] OR intraperitoneal [ot]	9	#6 OR #7 OR #8	9	subcutaneous*:ti,ab,kw
10	(peritone* or intraperitone*).ti,ab,kw.	10	#7 OR #8 OR #9	10	TITLE-ABS-KEY (subcutaneous*)	10	insulin:ti,ab,kw
11	7 or 8 or 9 or 10	11	Subcutaneous*[tw]	11	TITLE-ABS-KEY (insulin)	11	inject*:ti,ab,kw
12	exp subcutaneous drug administration/	12	Insulin [mh]	12	TITLE-ABS-KEY (inject*)	12	infus*:ti,ab,kw
13	subcutaneous.ti,ab,kw.	13	Insulin [tiab] OR Insulin [ot]	13	TITLE-ABS-KEY (infus*)	13	admin*:ti,ab,kw
14	12 or 13	14	#12 OR #13	14	TITLE-ABS-KEY (admin*)	14	absorption:ti,ab,kw
15	exp insulin derivative/	15	Drug administration routes[mh]	15	TITLE-ABS-KEY (absorption*)	15	therap*:ti,ab,kw
16	insulin.ti,ab,kw.	16	injection[tiab] OR injection[ot]	16	TITLE-ABS-KEY (therap*)	16	treatment:ti,ab,kw
17	15 or 16	17	infusion[tiab] OR infusion[ot]	17	TITLE-ABS-KEY (insulin treatment)	17	insulin infusion system*:ti,ab,kw
18	exp injection/	18	administration[tiab] OR administration[ot]	18	TITLE-ABS-KEY (pump)	18	pump:ti,ab,kw
19	infus*.ti,ab,kw.	19	absorption[tiab] OR absorption[ot]	19	#12 OR #13 OR #14 OR #15 OR #16 OR #17 OR #18	19	#11 or #12 or #13 or #14 or #15 or #16 or #17 or #18
20	admin*.ti,ab,kw.	20	therap*[tiab] OR therap*[ot]	20	#5 AND #9 AND #10 AND #11 AND #19	20	#5 and #8 and #9 and #10 and #19
21	absorption.ti,ab,kw.	21	treatment[tiab] OR treatment[ot]				
22	inject*.ti,ab,kw.	22	Infusion pump[mh]				
23	exp therapy/	23	pump[tiab] OR pump [ot]				
24	therap*.ti,ab,kw.	24	#15 OR #16 OR #17 OR #18 OR #19 OR #20 OR #21 OR #22 OR #23				
25	exp insulin treatment/	25	#6 AND #10 AND #11 AND #14 AND #24				
26	exp pump/						
27	insulin pump.ti,ab,kw.						
28	18 or 19 or 20 or 21 or 22 or 23 or 24 or 25 or 26 or 27						
29	6 and 11 and 14 and 17 and 28						

Changes in the systematic review compared to the Protocol

During the data evaluation, we decided to restrict the results to a comparison of the effects of continuous subcutaneous insulin infusion (CSII) and continuous intraperitoneal insulin infusion (CIPII) only, as the pharmacokinetics (and possibly the pharmacodynamics) of multiple daily injections (MDI) differ between the two routes of administration. In general, we observed improved glycaemic control when continuous insulin delivery systems (either intravenous, subcutaneous, or intraperitoneal) were compared to MDI of insulin [1-4] and we concluded that reporting a comparison between CIPII and MDI or mixed MDI/CSII treatment would introduce unnecessary bias. The inability to compare MDI and CSII is also reflected by the differences in pharmacokinetics of the various insulin regimes used with MDI (short-, medium-, or long-lasting) versus the exclusive use of continuous short-lasting insulin infusions during CSII. Therefore, bias could be introduced based on differences in the daily profile of insulin delivery or the type of insulin used, and not just the route of administration *per se*. Furthermore, studies with missing or insufficient information pertaining to the methods of insulin delivery were also excluded.

In the Protocol, one of the outcomes was identified as 'Different locations of IP and SC delivered insulin'. After the data extraction, however, we observed that in some included studies [5, 6], patients had been given the choice about where the intraperitoneal (IP) catheter was inserted; in addition, the location could also be changed during the study (e.g., after the replacement of an implanted pump). For instance, in one study, the pumps were placed on the left side of the abdomen in the IP space because all the participants were right-handed [6]. Therefore, the main outcome described as 'Insulin absorption and parameters that can affect it: Different location of IP and subcutaneous (SC) delivered insulin; Different types of insulin used in the same location' could not be evaluated.

Regarding the case-control studies, we revised the inclusion criteria, from "we need at least one before CIPII-period and one after CIPII-period measurement point", to 'the study is included if measurements from CSII and CIPII patients/periods are reported separately'.

During the data collection, we demoted some of the primary outcomes (Stated in the Protocol) to secondary outcomes. Consequently, we made a decision based on the clinical relevance of the results. The original primary and secondary outcomes were described as follows:

Primary outcomes

The main outcomes in the included studies were: (1) Glycaemic control (glycated haemoglobin A1c (HbA1c) levels, self-monitoring of blood glucose (SMBG), fasting blood glucose (BG) and mean BG levels, hypoglycaemic and hyperglycaemic events, time spent in normoglycaemia, and glucose variability), (2) Insulin

levels (fasting insulin level, time until maximum insulin level, maximum insulin level, and elevation of insulin level after administration of a pre-meal insulin bolus), (3) Mean daily insulin requirement.

Secondary outcomes

Secondary outcomes were physiological variables other than the primary outcomes, including the following: (1) Intermediate metabolites (levels of triglycerides, cholesterol, free fatty acids, lactate, ketone bodies, and apolipoproteins), (2) Counterregulatory hormones (levels of glucagon, catecholamines, growth hormone, insulin-like growth hormones, and binding proteins), (3) Other metabolic outcomes (levels of anti-insulin antibodies (AIA), sex hormone binding globulin (SHBG), and plasminogen activator inhibitor-1 (PAI–1)), (4) Any technical and/or physiological complications reported during the CIPII treatment.

Extended information not described in the results

Excluded articles and reasons for exclusion

The search strategy identified 1,517 records. After the removal of duplicates and irrelevant articles, 108 potentially eligible articles remained for consideration (Fig 1).

After full-text and manual reference screening of potential articles and the evaluation of the quality of evidence, 105 articles were included. After additional searches, four more articles were considered for inclusion. After the introduction of additional exclusion criteria (See section above titled: 'Changes in the Systematic review compared to the Protocol'), 70 of the 109 articles were excluded for the following reasons:

- Forty-one articles did not report CSII and MDI patients/periods separately [7-47];
- two articles reported on only MDI and CIPII, but not CSII [48, 49];
- four technical reports lacked information on physiological effects [50-54];
- two reports were review articles [55, 56];
- three articles compared intravenous (IV) versus IP insulin administration [57-59];
- two articles exhibited biased reporting of the distribution of patients per group [60, 61];
- one article did not provide information about the distribution of patients per groups [62];
- five articles were missing information about pre-implantation SC insulin infusion/injection [63-67];
- one article was an epidemiological study [68];
- two articles assessed patients with a mixture of diabetes mellitus type 1 (DM1) and diabetes mellitus type 2 (DM2) [69, 70];
- two articles did not provide any relevant information [71, 72];

- one article assessed patients treated with IP insulin injections (IPII) delivered as separate boluses, not as a continuous infusion as was used for CIPII [73];
- two articles assessed a CIPII treatment period lasting less than one month [74, 75];
- one article investigated an SC peritoneal access device (SPAD). SPAD allows for absorption of insulin at the tissue close to the peritoneal lining, not from the inside of the peritoneal cavity [76];
- one article did not mention the length of the CSII and CIPII-periods [77].

In the second literature search (follow-up), which screened for studies published in 2016 to 2018, 209 additional records were identified. After the exclusion of irrelevant articles, only one additional article was included in the systematic review [78]. In the third literature search (follow-up) in which we screened studies from the year 2019, 84 additional records were identified. After the removal of all irrelevant articles, no additional articles were included in the systematic review. In the fourth literature search (follow-up) in which we screened for the studies published from 2017 to 2020, 241 records were identified. After the exclusion of irrelevant articles, four records were considered for inclusion; ultimately, only one was included in the systematic review.

In total, 32 studies from 39 articles were included in the systematic review.

Risk of biases

Some studies [79-81] included participants who received MDI therapy, however, the data were also separately available for the CSII and CIPII treatment groups.

One study that provided data for the CSII-period vs. the CIPII-period used a programmable implantable medication system (PIMS). Afterwards, the PIMS was changed to the MiniMed Implantable Pump (MIP). Because two different CIPII pumps were used, the data from the period in which patients were treated with a PIMS insulin pump were compared with the data from the CSII-period. Data pertaining to the complications experienced during the CIPII-period were extracted from both the PIMS and MIP periods [6]. One study included two different experiments with overlapping patient groups; however, data from the study’s second experiment fulfilled our inclusion criteria, and the data for the CIPII and CSII treated patients were extracted [82].

One study did not report essential unit information regarding the daily insulin expenditure [83]. However, we assumed that the insulin expenditure in Table 2 was reported as U/24 hours.

One study did not provide unit information for the mean amplitude of glycaemic excursion (MAGE) [84]. To try to obtain the missing information, we used the reference for the MAGE from the article provided by the authors [85], where, the reported unit was listed as ‘mg/100 mL’.

One study did not state whether the error of the reported data was listed as the SD or the standard error (SE) [86]. Another study did not describe the statistical analysis method [87]. A third study did not state the mean values of the patients' HbA1c levels [5]. Consequently, these studies were excluded from the HbA1c meta-analyses.

In one study, the units for BG were defined differently in Table 2 (mg/mL) and in the main text (mg/dL); we assumed the correct units to be mg/dL, and those values were used in the analysis. The percentage of blood glucose levels that were high, low or in the normal range were not available due to missing information about the definition of the normal range in that study [88].

Two independent studies provided very similar base line data, with similar methodological description and with identical study periods. However, the authors did not state whether the data in these reports were derived from the same study, from two separate studies, or whether they contained partially overlapping patient populations [89, 90]. E-mails, sent to the authors by IDF to verify the uniqueness of these two studies were not answered.

Another two studies provided similar base line data, with the same year of publication [91, 92]. Those two studies had identical male: female sex ratios, and age ranges (Table 1); however, they differed in the lengths of the follow-up periods, and the baseline HbA1c levels. Therefore, we assumed that the follow-up periods in these two reports were from different time periods, although we cannot discount the possibility of an overlap in the follow-up for these two studies. One of these articles [91] reported HbA1c levels (Fig 2) in the addition to the insulin expenditure, the anti-insulin antibody levels, and complications that occurred during the CIPII-period (Table S2.6). From the other article [92] the data were derived from a figure showing changes in insulin levels, and it was not possible to determine the SD. Therefore, these data were not included in the meta-analysis.

In one study, the data reported in the text were given as the geometric mean values, whereas we used the estimated mean value (Table 2) [93].

One study was a multinational, open, randomised, controlled, crossover study [5]. Due to a high dropout rate (15 out of 30 patients in the CIPII group and 9 out of 30 in the CSII group), the results were analysed as a randomised follow-up study between two parallel treatment groups (i.e., before the crossover).

One study did not provide a definition of severe hypoglycaemia. During the extended periods of the study's reporting (including conference posters presentations for data at 3, 6, 12, 24 months), the number of severe hypoglycaemic events reportedly increased during the CSII-period [94-97].

Results of the search

The primary search strategy identified 1,517 reports, and 21 more were added after screening of the reference lists. After abstract screening, 105 potentially eligible reports remained (Fig 1). After additional searches, four more articles were considered for inclusion in the analysis.

When applying the additional exclusion criteria (which are described above in the “Changes in the Systematic review compared to the Protocol), 70 of the 109 reports were excluded; these are described in the ‘Excluded reports and reasons for exclusion’ section above.

In total, 38 reports from 32 studies, including one report in Italian [98] and one in German [99], were included (Fig 1).

Data extraction and quality assessment

There was considerable heterogeneity among the studies (Tables S2.1 – S2.6), although most were crossover studies (23 of 32 studies), with at least three months of CSII treatment, followed by 1.5 to 14 months of CIPII treatment. More men (n = 167; 55 %) than women (n = 136; 45 %) were included in the CIPII-period. Thirty out of 32 studies reported the sex of participants, and the ages ranged from 19 to 82 years (Table 1). In the nine studies that reported age separately for each sex, the mean age range (min – max) was 37.1 years (19 – 67) in men and 32.6 years (18 – 50) in women.

Twenty-four studies originated from single European countries (Table 1), four originated from a French multicentre study (EVADIAC: EVAluation dans le Diabète des Implants ACtifs Group) [86, 88, 100, 101], three studies were from the USA [6, 83, 102], and one was a multinational study [5] (Table 1).

All results of these studies are summarised in Tables S2.1 – S2.13.

Qualitative data analysis

Primary outcome: Glycaemic control

In addition to including patients who were already being treated with CSII, one randomised [5] and six nonrandomised studies [6, 84, 88, 91, 103, 104] provided participants with an additional CSII follow-up before transitioning them to the CIPII treatment. In three of these studies, the HbA1c levels decreased during this additional CSII follow-up period [5, 103, 104].

Randomised follow-up studies

One prospective, randomised, follow-up study (for details see the section titled, ‘Risk of biases’) observed equivalent reduction in HbA1c levels in the two treatment groups (CIPII: - 0.5 %; CSII: - 0.6 %, p = 0.374) and no difference in SMBG values during the twelve months of CIPII treatment and the six months of CSII treatment [5].

Non-randomised and retrospective crossover studies

Glycated haemoglobin A1c

Significantly lower ($p < 0.05$) mean HbA1c levels were reported during the CIPII treatment period in eight prospective studies and one retrospective study. HbA1c level decreased from 83.6 – 56.3 mmol/mol (9.8 – 7.3 %) to 60.7 – 44.3 mmol/mol (7.7 – 6.2 %) (Fig 2) [6, 83, 87-90, 94-97, 105].

No differences in mean HbA1c levels were reported in five studies [98, 101, 102, 106-108]. In one study the HbA1c levels decreased after three months of CIPII treatment (54.1 mmol/mol (7.1 %)), whereas no statistical difference was observed after 12 months of CIPII treatment compared to the previous CSII treatment (58.5 vs. 59.6 mmol/mol (7.5 % vs. 7.6 %)) [101]. Five studies did not report statistical analyses comparing the two treatments (Table S2.1) [86, 91, 103, 104, 109]. The lack of SD/SE data resulted in the exclusion of three of these studies from the meta-analysis (Fig 2) [5, 86, 87].

Self-monitored blood glucose

Three studies that reported on SMBG concentrations showed a decrease in BG levels from 7.8 – 10.5 mmol/L to 7.4 – 8.0 mmol/L ($p < 0.05$) [83, 88, 96, 102], whereas four studies reported no difference in SMBG levels (Fig S1, Table S2.1) [6, 84, 86, 108]. However, in one of these studies, SMBG levels decreased during the first 16 months of CIPII treatment, but was equal to those following CSII after 18 months [6]. Three studies did not conduct statistical testing to compare the two treatments [103, 104, 109].

Glucose variability

One study reported a lower MAGE value during the CIPII treatment period compared to the CSII treatment period (6.9 vs. 9.5 mmol/L, $p < 0.005$) [84]. Another five studies reported a decrease in SD of BG levels during CIPII-period compared to the CSII-period (3.0 – 3.8 mmol/L vs. 3.4 – 5.1 mmol/L, $p < 0.04$) (Table S2.1) [86, 88-90, 108].

Continuous glucose monitoring

One study reported decreased mean BG levels (measured by continuous glucose monitoring (CGM)) (8.3 vs. 10.5 mmol/L, $p = 0.004$), increased time spent in normoglycaemia (3.9 –10.0 mmol/L, $p = 0.001$), and a narrower BG range (4.4 – 7.8 mmol/L, $p = 0.03$) in the CIPII-period than in the CSII-period [78]. Another study with CGM reported an increase in the time spent in normoglycaemia (3.9 – 10.0 mmol/L, $p = 0.027$) during the CIPII-period [94-97].

One study reported decreased pre-prandial BG levels ($p < 0.05$) [88], whereas another observed decreased post-prandial BG levels ($p < 0.01$) [87]. Two studies reported no difference in pre-prandial BG levels [86, 88]

and two studies reported no difference in post-prandial BG levels during the CIPII-period [86, 88]. One study did not conduct statistical comparison of the two treatments [103].

Case-control studies

Among the four included case-control studies that reported HbA1c levels, no difference was observed between the treatment groups (Fig 2) [82, 88, 99, 110-112]. One of these studies also reported no difference in pre-prandial and post-prandial BG levels [82].

Case studies

Only one case study was included, which reported no difference in glycaemic control between the CIPII and CSII treatments (Table S2.1) [113]. Due to large SD values, these results could not be included in the meta-analysis.

Primary outcome: Hypo-/ hyperglycaemia

Randomised follow-up studies

In one study, the frequency of severe hypoglycaemia (requiring hospitalization or IV glucose administration, or events accompanied by unconsciousness or seizure) was significantly reduced during the CIPII compared to the CSII follow-up periods (0.35 vs. 0.86 events/patient-years, $p = 0.013$). During the first three months after the initiation of CIPII treatment, the frequency of severe hypoglycaemic events was unchanged, whereas it was reduced in the subsequent nine months (0.72 vs. 0.15 events/patient-years). During CSII treatment the frequency of severe hypoglycaemia was 1.6 events per one patient-year at baseline which was reduced to 0.86 events per one patient-years during the CSII follow-up period [5]. No difference in the frequency of hypoglycaemic episodes (SMBG level < 3 mmol/L) was observed during the CIPII treatment period. Furthermore, no difference was observed between the first three months and the subsequent nine months of CIPII treatment (Tables S2.1 and S2.8) [5]. Statistical analyses were only reported for comparison between the CIPII and CSII treatment groups; no within-group analyses were performed.

Non-randomised crossover studies

Severe hypoglycaemia and hypoglycaemic coma

Four studies recorded severe hypoglycaemia, but none conducted any statistical analyses [6, 81, 94-98]. One study reported no difference in the frequency of hypoglycaemic coma events (CIPII: 0 vs. CSII: 0.54 events/patient-year) [81]. Another study reported that the frequency of severe hypoglycaemia (requiring assistance) was 0.43 events per one patient-year during the CIPII-period while no episodes of hypoglycaemic coma were observed [6].

One study reported 1.5 severe hypoglycaemic (requiring assistance) events per one patient-year during the CIPII compared to the 12 events per one patient-year during CSII-period [94-97]. Another study reported no severe hypoglycaemic (requiring assistance) events during the CIPII-period [81], and one study reported no difference in the occurrence of severe hypoglycaemia [98].

Hypoglycaemia

One study reported a reduction in the time spent in hypoglycaemia during CIPII-period (SMBG level < 3.9 mmol/L, $p < 0.05$), whereas the duration of time spent with SMBG levels < 2.8 mmol/L was similar between the treatment periods [84]. On the contrary, one 24-hour BG profile study reported no difference in the time spent in hypoglycaemia (BG < 3.8 mmol/L, measured by CGM) [78]. Similarly, two other studies reported no difference in hypoglycaemic events (SMBG level < 3.0 mmol/L) [89, 90].

One study reported at least one hypoglycaemic event (SMBG level < 3.3 mmol/L) per patient during CIPII-period [6].

Hyperglycaemia

One study using CGM [78] reported less time spent in hyperglycaemia (BG > 10 mmol/L, $p < 0.05$), whereas another study using SMBG reported no difference [84]. However, both reported a reduction in the time spent in severe hyperglycaemia (BG > 14 mmol/L, $p < 0.05$, measured by SMBG and CGM) during CIPII-period. (Tables S2.1 and S2.8) [78, 84].

Primary outcome: Insulin levels

Randomised crossover and follow-up studies

In one study, five patients being treated during the CIPII-period were crossed over to receive 96-hour CSII treatment temporarily. Insulin was infused for 12 hours at a fixed basal rate. Fasting serum free insulin levels were decreased during the CIPII-period compared to the CSII-period (30.8 vs. 45.0 pmol/L, $p < 0.001$) [100]. Subsequently, insulin was infused a rate of 15 nmol/h for 150 minutes, then 42 nmol/h for the following 150 minutes. During these two short-term periods with increased infusion rates, the rate of appearance (Ra) of insulin in the systemic circulation was greater during CIPII treatment ($p < 0.05$ and $p < 0.01$, respectively) [100].

No difference in the mean daily insulin requirement was observed in a prospective study with 36 patients, although no statistical analyses were performed [5].

Non-randomised crossover studies and follow-up studies

Two studies reported lower fasting insulin levels ($p < 0.05$ and $p < 0.01$) [89, 90], despite a higher basal insulin infusion rate during CIPII ($p = 0.02$) [89]. Two studies reported no difference in fasting insulin levels between

the two periods [87, 109]. Another two studies did not perform statistical comparisons between treatments [103, 104]. Two studies (with 20-hour and 16-hour insulin profiles) reported decreased night-time insulin levels during CIPII (127.8 vs. 163.2 pmol/L, $p < 0.05$; and 70.1 vs. 128.5 pmol/L, $p < 0.01$, respectively) [87, 103].

Two studies reported earlier post-bolus maximum insulin levels, peripherally, during the CIPII-period (60 vs. 133.6 minutes, $p < 0.006$ [92]; and 60 vs. 180 minutes, $p < 0.05$ [87]). The latter study reported increased maximum insulin levels during the CIPII-period (179.18 vs. 125.01 pmol/L, $p < 0.05$) [87].

Furthermore, during the CIPII-period, insulin levels returned to baseline values three hours after administration of a pre-breakfast bolus, whereas during the CSII-period, the post-bolus insulin level remained elevated five-and-half hours later [87].

One study that performed insulin clamp testing reported no difference in the maximum insulin levels between the periods; however, the first measurement was recorded 30 minutes after the administration of insulin boluses [89]. One study reported increased insulin levels ($p < 0.05$) during exercise in those receiving CSII, although, insulin levels did not change during exercise in the CIPII group [90].

One study reported a lower total area under curve (AUC) (16 hours) (72 vs. 100 mU/L/h, $p < 0.01$) and a lower night-time AUC (12 vs 36 mU/L/h, $p < 0.01$) during the CIPII period. The AUC following administration of an insulin bolus did not differ between the periods; however, the duration of the period for which the AUC was calculated was not specified [87].

In two studies, day-time mean insulin requirements were increased ($p < 0.05$) during CIPII-period [86, 108]. However, in one of these studies, the insulin requirement was increased only during the first two months of CIPII treatment before decreasing to levels that were similar to those in the previous CSII-period [108].

Other studies reported no change in insulin requirements between the periods, 12 of which performed statistical analyses [83, 84, 89, 90, 94-98, 101, 102, 105-109] (Table S2.2.).

On the contrary, one 24-hour closed-loop artificial pancreas study reported increased insulin delivery during closed-loop CIPII than during closed-loop CSII (43.7 U vs. 32.3 U, $p < 0.001$) [78].

Case-control studies

One study reported decreased mean night-time insulin levels in the CIPII-treated patients (65.56 vs. 86.53 pmol/L, $p < 0.005$) [99], whereas two studies reported no difference in fasting insulin levels between the two groups [82, 114].

One study reported earlier peaking of post-bolus (0.15 U/kg) insulin levels in CIPII-treated patients (30 minutes vs. 60 minutes, p-value not reported), increased maximum insulin levels (263.91 vs. 145.84 pmol/L

(significance between groups starting 30 minutes after bolus administration, $p < 0.05$)), and a decreased duration of elevated insulin levels (180 minutes vs. 240 minutes, p-value not reported) [82].

No differences in the mean daily insulin requirement were reported in three studies that performed statistical analyses [99, 110-112, 114] (Table S2.2).

Case reports

One case report showed no difference in daily insulin requirements [113].

Secondary outcomes: Intermediate metabolites

All reports that analysed intermediate metabolites are summarised in Table S2.3.

Non-randomised crossover studies

One study reported decreased total cholesterol levels after six months of the CIPII-period compared to those in the CSII-period (4.56 mmol/L vs. 4.85 mmol/L, $p = 0.044$) [102]. In the remaining six studies, no differences in total cholesterol levels were observed after six weeks to one year of CIPII treatment (Fig S2) [83, 84, 98, 106-109].

In one study, high-density lipoprotein (HDL)-cholesterol levels were lower during CIPII-periods compared to the CSII-periods (1.2 mmol/L vs. 1.4 mmol/L, $p < 0.05$) [84]. In five studies, no difference in HDL-cholesterol levels was observed between the periods [83, 98, 102, 106-108]. No difference in low-density lipoprotein (LDL)-cholesterol levels was observed in four studies [98, 102, 106-108].

One study reported an increase in fasting serum triglyceride levels after the CIPII-period (1.5 mmol/L vs. 0.9 mmol/L, $p < 0.005$) [84]. In six studies, no difference in triglyceride levels was observed between the two periods (Fig S3) [83, 98, 102, 106-109].

The chylomicron remnant levels, the ratio of retinyl ester: apoB lipoproteins, and the HDL compositions reported in the studies are provided in Table S2.3.

Case-control studies

One study reported decreased fasting free fatty acid (FFA) levels during the CIPII-period compared to the CSII-period ($p = 0.05$), whereas during the 60 minutes after the administration of a pre-meal insulin bolus, no changes in FFA levels were observed within the groups. However, decreased FFA levels were observed in the CIPII-period after administration of a pre-meal insulin bolus ($p = 0.05$) [82].

The measurements of lactate, vitamin D metabolites, creatinine, calcium, magnesium, phosphorus, parathyroid hormone, osteocalcin, and alanine reported in the studies are summarised in Table S2.3.

Secondary outcomes: counterregulatory hormones

All reported counterregulatory hormone analyses are summarised in Table S2.4.

Non-randomised crossover studies and follow-up studies

During a hypoglycaemic clamp, one study reported a significant incremental glucagon response during CIPII (p = 0.003), whereas the glucagon response was non-significant during CSII. Consequently, the maximal glucagon response was higher during CIPII (17.0 pg/mL vs.7.5 pg/mL, p = 0.048) [89]. One study reported increased glucagon levels post-exercise during CIPII-periods (p = 0.01); however, no difference in glucagon levels was observed between the CIPII and CSII-periods [90]. Significantly larger AUC was observed for the incremental glucagon response in the CIPII-period during hypoglycaemic insulin clamp testing and after intense exercise compared to pre-clamp testing and pre-exercise testing (44.4 pg/mL/h vs. 5.1 pg/mL/h, p = 0.027; and 23.4 pg/mL/h vs. 10.3 pg/mL/h, p = 0.04, respectively) [89, 90]. A significantly larger incremental post-exercise AUC compared to post-exercise (23.4 pg/mL/h vs. 10.3 pg/mL/h, p = 0.04) was also observed [90].

Two studies reported no change in epinephrine and norepinephrine incremental responses between the two periods during respective hypoglycaemic insulin clamp testing [89] or intensive exercise [90].

The results of measured changes in growth hormone (GH), insulin like growth factor 1 (IGF-1) and 2 (IGF-2), growth hormone binding protein (GHBP), insulin-like growth factor binding protein 2 (IGFBP-2) and 3 (IGFBP-3), and cortisol are summarised in Table S2.4.

Case-control studies

One study reported no difference in fasting and postprandial glucagon levels between the treatment groups [82].

Secondary outcome: Other metabolic outcomes

All other reported analyses are summarised in Table S2.5.

Non-randomised crossover and follow-up studies

Increased levels of anti-insulin antibodies (AIA) measured by enzyme-linked immunosorbent assay (ELISA), were observed after three and twelve months of the CIPII-period (39.3 % and 42.5 % vs. 23.7 %, respectively, p < 0.01), but not after 24 months [79, 80], and at three months of the CIPII-period in another study (11.0 % vs. 3.6 %, p < 0.05) [86]. No difference was observed in one study [91], and another reported no changes in the AIA levels (p-value not reported) [78].

One follow-up study observed increased AIA levels after six months of the CIPII-period vs. six months of the CSII-period (41.8 % vs. 24.9 %, p = 0.009), as measured by radioimmunoassay (RIA), although they observed no difference when AIA levels were measured by ELISA [115].

Studies reporting sex hormone binding globulin (SHBG) levels are summarised in Table S2.5.

Secondary outcome: Complications

All reported technical and physical complications are summarised in Table S2.6.

How to read the tables

The source column lists the main author and the year of publication. In cases where the authors and year of publication are the same for two studies, some additional information is provided in differentiation. Alternatively, when there is no information given in other columns, information is provided that could explain the missing data. For example, if there is no information provided under the 'Reported study objectives' and/or 'methodological quality' columns, it could be because information was extracted from a letter to the editor.

The 'Participant characteristics' column supplies information about the number of participants and some characteristics we believe are important for describing the actual patients. More detailed information can be found in the original publications.

In the 'Length of' column, we provide information about the duration of the CIPII and/or CSII-periods, and, if available, some information about patient follow-up. Most data are given as the means.

In the 'Reported study objectives' column we present the precise information as stated in the articles.

We extracted data from text, tables, and graphics, all of which is included it in the 'Outcomes' column. In cases, where information was missing, possible biases are indicated in the systematic review's Results section.

Some articles included figures showing measurements of continuous variables (for example, 16-hour measurements). From such figures, we extracted data from fasting periods and noted data that was significantly different between the two periods. If data for continuous variables measurements were not significantly different, it was mentioned in the Results without providing any additional data.

Units of the measurement are indicated after the CSII data (for example, HbA1C measurements, CIPII: 8.7; CSII: 8.8 %).

Definition of words used:

Increases means that in the CIPII-period, levels were statistically significantly higher ($p < 0.05$) than those in the CSII-period.

Decreases means that in the CIPII-period, levels are statistically significantly lower ($p < 0.05$) than those in the CSII-period.

Decreases/increases in both means that the values followed the same pattern when compared at different time-points.

No change means a statistically non-significant difference (p > 0.05) or the p-value not provided (ND). If possible, data are shown in parentheses.

M3, M6, and M12, for example, should be read as 'three months', 'six months', and 'twelve months'.

The 'Methodological quality' column contains quality assessment tools that are appropriate for that particular study.

Table S2.1. Intervention studies: Participant characteristics, description, outcomes: glycaemic control

	Source	Participant characteristics (Number, age (mean years), diabetes duration (mean years), sex (Male/Female), HbA1c (%), C-peptide, reasons to participate	Length of: CSII use, CSII follow-up, CIPII follow-up (weeks)	Reported study objectives	Outcomes (mean, p-value)	Methodological quality
Glycaemic control	**Randomised follow-up studies**					**Cochrane risk of bias tool (CRB):**
	Liebl et al. 2009 [5]	N = 60[a] (CIPII: 30 /CSII: 30)<br>Age: 50.5/45.3<br>Diabetes duration: 26.3/25.1<br>Sex: (male) 73 %/43 %<br>HbA1c: 8.2/8.3<br>C-peptide: ND<br>Reasons: Poor metabolic control	CSII use: ND<br>CSII f-u: 26<br>CIPII f-u: 52	Comparison of frequency of hypoglycaemia, severe hypoglycaemia, metabolic control, diabetic QoL and safety between CSII and CIPII in type 1 diabetic patients.	**HbA1c:** Decreases in both groups (CIPII: - 0.5; CSII: - 0.6 %, p=0.374)<br>**SMBG:** No change (CIPII: + 0.1; CSII: ± 0.0 mmol/L, p=NS)<br>**BG < 3 mmol/L:** No change (All CIPII-period: 118.2; M1-3: 138.1; M4-12: 108.9; CSII: 115.8 events/patient-years, p=NS)<br>**Severe hypoglycaemia:** Decreases (Before CIPII: 0.7; All CIPII-period: 0.35, M1-3: 0.72; M4-12: 0.15, p=ND; Before CSII: 1.6; CSII-period: 0.86 events/patient-years, p=ND; CIPII vs CSII-period: p=0.013)	CRB:<br>Unclear risk of bias: Random sequence generation, allocation concealment, blinding<br>Low risk of bias: Incomplete outcome data, selective reporting, treatment procedure
	**Non-randomised crossover studies**				**Strengthening the Reporting of Observational Studies in Epidemiology (STROBE) and Thomas quality assessment toll (QAT):**	
	Micossi et al. 1986 [84]	N = 6<br>Age: 38.8<br>Diabetes duration: 12.6<br>Sex: 3/3<br>HbA1c: 7.25<br>C-peptide: ≤ 0.02 pmol/mL<br>Reasons: Pmc	CSII use: 12<br>CSII f-u: 6<br>CIPII f-u: 6	To investigate the hormonal and metabolic patterns produced by CIPII in group of severely unstable DM1 who has previously responded poorly to CSII. To compare clinical and metabolic effects of CSII and CIPII.	**HbA1c:** Decreases (CIPII: 6.2; CSII: 7.25 % (CIPII: 44; CSII: 56 mmol/mol), p<0.05)<br>**SMBG:** No change (CIPII: 8.8; CSII: 9.7 mmol/l, p=NS)<br>**BG > 14 mmol/l**: Decreases (CIPII: 8.9; CSII: 16.1 %, p<0.05)<br>**BG > 10mmol/l:** No change (CIPII: 31.8; CSII: 44.7 %, p=NS)<br>**BG < 3.9 mmol/l:** Decreases (CIPII: 4.5; CSII: 6.2 %, p<0.05)<br>**BG < 2.8 mmol/l:** No change (CIPII: 1.2; CSII: 1.6 %, p=NS)<br>**MAGE:** Decreases (CIPII: 6.9; CSII: 9.5 mmol/L, p<0.005)	STROBE: 15/22<br>QAT:<br>Strong: Data collection methods, withdrawals and drop-outs<br>Moderate: Selection bias, study design<br>Weak: Confounders
	Beylot et al. 1987 [103]	N = 4<br>Age: 42<br>Diabetes duration: 21.5<br>Sex: 3/1<br>HbA1c: 7.6 (9.2 – 5)<br>C-peptide: ND<br>Reasons: Volunteers	CSII use: ND<br>CSII f-u: 8<br>CIPII f-u: 8<br>Washout: 1 day	To determine if IP insulin administration could, in addition to decreasing peripheral insulin levels, improve the insulin resistance of DM1.	**HbA1c [DT]:** No change (CIPII: 6.2; CSII: 6.5 % (CIPII: 44; CSII:48 mmol/mol), p=ND))<br>**SMBG [DT]:** No change (CIPII: 8.20; CSII: 8.77 mmol/l, p=ND)<br>**Pre-prandial BG**: No change (CIPII: 5.9; CSII: 5.4 mmol/L, p=ND)<br>**Endogenous glucose production in basal period:** No change (CIPII: 2.92; CSII: 2.93mg/kg/min, p=ND)<br>**Glucose utilization in basal period:** No change (CIPII: 3.30; CSII: 3.62 mg/kg/min, p=ND)	STROBE: 15/22<br>QAT:<br>Strong: Data collection methods, withdrawals and drop-outs<br>Moderate: Selection bias, study design, confounders
	Wredling, Adamson et al. 1991 (technical report) [91]	N = 6<br>Age: 41.3<br>Diabetes duration: 23.2<br>Sex: 4/2<br>HbA1c: 8.7<br>C-peptide: Neg<br>Reasons: Pmc	CSII use: 52+<br>CSII f-u: 8 (n=3)<br>CIPII f-u: median 72	To determine the efficacy of a new percutaneous device.	**HbA1c*:** No change (CIPII: 7.6; CSII: 8.7 % (CIPII: 60; CSII: 72 mmol/mol), p=ND)	STROBE:15/22<br>QAT:<br>Moderate: Selection bias, study design, data collection method<br>Weak: Withdrawals and drop-outs<br>Unclear: Confounders

Legends: CSII, continuous subcutaneous insulin infusion; CIPII, continuous intraperitoneal insulin infusion; ND, no data available; Pmc, Poor metabolic control; NS, Not significant; BG, blood glucose; MPG, mean plasma glucose; SMBG, self-monitored BG; MAGE, mean amplitude of glycaemic excursion; [a] dropouts in this study (at the end of the periods N= 36 (CIPII: 15 /CSII: 21); *, HbA1c calculated as mean of all determinations (every 4 weeks); [DT]: data calculated from table.

Table S2.1. (Continued)

	Source	Participant characteristics (Number, age (mean years), diabetes duration (mean years), sex (Male/Female), HbA1c (%), C-peptide, reasons to participate	Length of: CSII use, CSII follow-up, IPII follow-up (weeks)	Reported study objectives	Outcomes (mean, p-value)	Methodological quality
**Glycaemic control**	**Non-randomised crossover studies**				**Strengthening the Reporting of Observational Studies in Epidemiology (STROBE) and Thomas quality assessment toll (QAT):**	
	Georgopoulos et al. 1992 [83]	N = 7<br>Age: 27<br>Diabetes duration: 12<br>Sex: 5/2<br>HbA1c: 9.8<br>C-peptide: ND<br>Reasons: ND	CSII use: ND<br>CIPII f-u: 52-60	To investigate whether long-term improved glycaemic control by intraperitoneal insulin infusion normalizes the compositional abnormalities of triglyceride (TG)-rich lipoproteins in DM1.	**HbA1c:** Decreases (CIPII: 7.7; CSII: 9.8 % (CIPII: 61; CSII: 84 mmol/mol), p<0.001)<br>**SMBG:** Decreases (CIPII: 7.7; CSII: 10.5 mmol/L, p<0.02)	STROBE: 11/22<br>QAT:<br>Strong: Data collection methods, withdrawals and drop-puts<br>Moderate: Selection bias, study design, confounders
	Pitt et al. 1992 [6]	N = 10<br>Age: 33.2<br>Diabetes duration: 23.2<br>Sex: 8/2<br>HbA1c: 9.1<br>C-peptide: Neg<br>Reasons: Volunteers	CSII use: 12+<br>CSII f-u: 8<br>CIPII f-u: 240	Document nearly 70 patient-years of experience with IP insulin delivery, with longest over 5 years, in 21 patients with type I diabetes.	**HbA1c [FF]:** Decreases (CIPII: M18: 8.0, p<0.05; M16: 8.6, p=NS; M12: 8.0, p<0.05; M6: 7.5, p<0.05; CSII: 9.1 % (CIPII: M18: 64; M16: 70; M12: 64; M6: 58; CSII: 76 mmol/mol))<br>**SMBG [FF]:** No change (CIPII: M18: 7.8, p=NS; M16: 7.7, p<0.05; M12: 7.8, p<0.05; M6: CIPII: 7.2, p<0.05; CSII: 8.9 mmol/L, p<0.05)<br>**BG < 3.3 mmol/L:** No change (ND)<br>**Severe hypoglycaemia**: 3 episodes during 7 years in CIPII-period<br>**Hypoglycaemic coma:** No events occurred during CIPII-period	STROBE: 18/22<br>QAT:<br>Strong: Confounders, withdrawals and dropouts<br>Moderate: Selection bias, study design, data collection methods
	Renard et al. 1993 [81]	N = 8<br>Age: 41.6<br>Diabetes duration: 14.0<br>Sex: 6/2<br>HbA1c: ND<br>C-peptide: Neg<br>Reasons: Volunteers	CSII use: 52<br>CIPII f-u: 52	To gain experience in assessing the feasibility of therapeutical mode in DM1 patients, who had previous long-term experience of ambulatory SC insulin delivery portable devices.	**SMBG:** Based on mixed results (MDI and CSII) data is not included in the review<br>**Severe hypoglycaemia**: Decreases (CIPII: 0; CSII: 0.54 events/patient-year, p=ND)<br>**Hypoglycaemic coma:** Decreases (CIPII: 0; CSII: 0.54 events/patient-years, p=ND)<br>**Ketoacidosis:** Decreases (CIPII: 0; CSII: 0.14 events/patient-years, p=ND)	STROBE: 19/22<br>QAT:<br>Strong: Confounders, data collection methods<br>Moderate: Selection bias, study design<br>Weak: Withdrawals and drop-outs
	Georgopoulos et al. 1994 [102]	N = 8<br>Age: 37<br>Diabetes duration: 21.6<br>Sex: 5/3<br>HbA1c: 9.4<br>C-peptide: ND<br>Reasons: ND	CSII use: ND<br>CIPII f-u: 26	Test hypothesis that CIPII will decrease the level of circulating chylomicron remnants in patients with DM1.	**HbA1c:** No change (CIPII: 8.7; CSII: 9.4 %, p=NS)<br>**SMBG:** Decreases (CIPII: 7.4; CSII: 7.82 mmol/l, p=0.027)	STROBE: 14/22<br>QAT:<br>Strong: Data collection method, withdrawals and dropouts<br>Moderate: Study design, confounders<br>Unclear: Selection bias
	Lassmann-Vague et al. 1994 (short communication) [104]	N = 11<br>Age: 34.4<br>Diabetes duration: 22.4<br>Sex: 5/6<br>HbA1c: 7.0<br>C-peptide: Neg<br>Reasons: ND	CSII use: 26+<br>CSII f-u: 4<br>CIPII f-u: 12	ND	**SMBG**: No change (CIPII: M1: 7.9; M3: 8.3; CSII: 8.3 mmol/L, p=ND)<br>**HbA1c**: No change (CIPII: 6.8; CSII: 6.9 %, p=ND)	NP

Legends: CSII, continuous subcutaneous insulin infusion; CIPII, continuous intraperitoneal insulin infusion; ND, no data available; NS, Not significant; BG, blood glucose; SMBG, self-monitored BG; Severe hypoglycaemia, requiring assistance; Ketoacidosis, vomiting and/or nausea in the presence of hyperglycaemia (BG>13 mmol/L), more detains in the main article; [FF], data extracted from figure.

Table S2.1. (Continued)

	Source	Participant characteristics (Number, age (mean years), diabetes duration (mean years), sex (Male/Female), HbA1c (%), C-peptide, reasons to participate	Length of: CSII use, CSII follow-up, IPII follow-up (weeks)	Reported study objectives	Outcomes (mean, p-value)	Methodological quality
Glycaemic control	**Non-randomised crossover studies**				**Strengthening the Reporting of Observational Studies in Epidemiology (STROBE) and Thomas quality assessment toll (QAT):**	
	Raccah et al. 1994 (letter) [109]	N = 11<br>Age: 34.4<br>Diabetes duration: 22.3<br>Sex: 6/5<br>HbA1c: 6.9<br>C-peptide: ND<br>Reasons: ND	CSII use: 12<br>CIPII f-u: 40	ND	**HbA1c:** No change (CIPII: M10: 6.3; M3: 6.8; CSII: 6.9 %, p=ND)<br>**SMBG:** No change (CIPII: M3: 8.3; M10: 8; CSII: 8.3 mmol/L, p=ND)	NP
	Schnell et al. 1994 [105]	N = 5<br>Age: 35.8<br>Diabetes duration: 20.2<br>Sex: 1/4<br>HbA1c: 9.8<br>C-peptide: ND<br>Reasons: ND	CSII use: 156-364<br>CIPII f-u: 52	To compare insulin demands during 24 h in IPII and CSII patients.<br>To compare HbA1c levels in CIPII and CSII patients.	**HbA1c:** Decreases (CIPII: M12: 8.5, p<0.05; M3: 8.6, p<0.05; CSII: 9.8 %)	STROBE: 17/22<br>QAT:<br>Strong: Withdrawals and drop-outs<br>Moderate: Selection bias, study design, confounders, data collection method
	Guerci et al. 1996 [108]	N = 14<br>Age: 40.0<br>Diabetes duration: 16.4<br>Sex: 9/5<br>HbA1c: 6.1<br>C-peptide: Neg<br>Reasons: Volunteers	CSII use: 52+<br>CIPII f-u: 16	To determine the effects of IPII on qualitative lipoprotein abnormality.	**HbA1c:** No change (CIPII: 5.9; CSII: 6.1 %, p=NS)<br>**SMBG:** No change (CIPII: 7.55; CSII: 7.78 mmol/L, p=NS)<br>**SD of BG:** Decreases (CIPII: 3.0; CSII: 3.4 mmol/L, p<0.01)	STROBE: 16/22<br>QAT:<br>Strong: Selection bias, confounders, data collection method, withdrawals and drop-outs<br>Moderate: Study design
	Hanaire-Broutin et al 1996 [101]	N = 18<br>Age: 43.0<br>Diabetes duration: 20.0<br>Sex: 11/7<br>HbA1c: 7.6<br>C-peptide: Neg<br>Reasons: Volunteers	CSII use:128<br>CIPII f-u: 52	To evaluate the impact of IP insulin therapy, which results in preferential insulin absorption by the portal system, on the hepatic growth hormone-resistant state of DM1.	**HbA1c:** No change (M12: 7.5, p=NS; M3: 7.1, p<0.02; CSII: 7.6 %)	STROBE:16/22<br>QAT:<br>Strong: Study design, data collection methods<br>Moderate: Selection bias, confounders, withdrawals and drop-outs
	Lassmann-Vague et al. 1996 [87]	N = 11<br>Age: 36.3<br>Diabetes duration: 17.8<br>Sex: 6/5<br>HbA1c: ND<br>C-peptide: ND<br>Reasons: ND	CSII use: ND<br>CSII f-u: ND<br>CIPII f-u: 8	To compare plasma free insulin levels achieved in patients with DM1 chronically treated with CSII and CIPII.	**HbA1c:** Decreases (CIPII: 6.9; CSII: 7.7 %, p<0.001)<br>**16-hour blood glucose profile:**<br>**BG during night (12:00 am):** No change (CIPII: 9.1; CSII: 9.3 mmol/L, p=ND)<br>**4:00 am:** No change (CIPII: 7.7; CSII: 7.9 mol/L, p=ND)<br>**Post-prandial BG (9:30 am):** Decreases (CIPII: 7.8; CSII: 12.7 mmol/L, p<0.01)<br>**3:00 pm:** Decreases (CIPII: 7.5; CSII: 12.8 mmol/L, p<0.01)	STROBE: 14/22<br>QAT:<br>Strong: Data collection method, withdrawals and drop-outs<br>Moderate: Selection bias, study design<br>Weak: Confounders

Legends: CSII, continuous subcutaneous insulin infusion; CIPII, continuous intraperitoneal insulin infusion; ND, no data available; NS, Not significant; BG, blood glucose; SMBG, self-monitored BG; SD of BG, standard deviation of BG.

Table S2.1. (Continued)

	Source	Participant characteristics (Number, age (mean years), diabetes duration (mean years), sex (Male/Female), HbA1c (%), C-peptide, reasons to participate	Length of: CSII use, CSII follow-up, IPII follow-up (weeks)	Reported study objectives	Outcomes (mean, p-value)	Methodological quality
Glycaemic control	**Non-randomised crossover studies**					**Strengthening the Reporting of Observational Studies in Epidemiology (STROBE) and Thomas quality assessment toll (QAT):**
	Pacifico et al. 1997 [98]	N = 8<br>Age: 35.1<br>Diabetes duration: 19<br>Sex: 5/4<br>HbA1c: 6.5<br>C-peptide: Neg<br>Reasons: Volunteers	CSII use: 12+<br>CIPII f-u: 52+	To evaluate the safety, the efficacy and the results after 3 years of CIPII	**HbA1c:** No change (M12: 6.6 CSII: 6.5 %, p=NS)<br>**Severe hypoglycaemia:** No change (CIPII: 0.11 events/patients/year CSII: ND)	STROBE:19/22<br>QAT:<br>Strong: Study design, data collection methods, selection bias<br>Moderate: Confounders, withdrawals and drop-outs
	Oskarsson et al. 1999 [90]	N = 7<br>Age: 42<br>Diabetes duration: 15<br>Sex: 5/2<br>HbA1c: 8.5<br>C-peptide: < 0.2 nM<br>Reasons: Pmc	CSII use: 26+<br>CIPII f-u: 47-82	To assess the clinical relevance of the blood glucose, hypoglycaemia, glucagon secretion during exercise by comparing glycaemic and hormonal responses to a 40-min bicycle exercise test at 60% of $VO_{2\,max}$ during CSII and CIPII in type 1 diabetic patients.	**HbA1c:** Decreases (CIPII: 7.1; CSII: 8.5 %, p<0.01)<br>**SD of BG (stability index):** Decreases (CIPII: 3.5; CSII: 5.1 mmol/L, p=0.02)<br>**BG < 3.0 mmol/L**: No change (CIPII: 0.7; CSII: 3.8 events/months, p=0.07)	STROBE:16/22<br>QAT:<br>Strong: Confounders, data collection methods, withdrawals and drop-outs<br>Moderate: Selection bias, study design
	Oskarsson et al. 2000 [89]	N = 7<br>Age: 42<br>Diabetes duration: 17<br>Sex: 5/2<br>HbA1c: 8.6<br>C-peptide: Neg<br>Reasons: Pmc	CSII use: 52+<br>CIPII f-u: 47-86	To expose the patients to an identical hyperinsulinemic clamp with special emphasis on the glucagon response in the same patients during continuous treatment with CSII and CIPII.	**HbA1c:** Decreases (CIPII: 7.2 CSII: 8.6 %, p<0.01)<br>**SD of BG**: Decreases (CIPII: 3.5; CSII: 5.1 to mmol/L, p=0.02)<br>**Pre-prandial BG**: No change (CIPII: 6.3; CSII: 6.2 mmol/L p=NS)<br>**BG < 3.0 mmol/l**: No change (CIPII: 0.7; CSII: 3.8 event/month, p=0.07)	STROBE: 16/22<br>QAT:<br>Strong: Confounders, data collection methods, withdrawals and drop-outs<br>Moderate: Selection bias, study design
	Duvillard et al. 2005 (Brief report) [106]<br>Duvillard et al 2007 [107]	N = 7<br>Age: 48<br>Diabetes duration: 17<br>Sex: 6/1<br>HbA1c: 7.34<br>C-peptide: ND<br>Reasons: ND	CSII use: ND<br>CIPII f-u: 12	Compare if replacement of SCII with IPII restores the normal physiological gradient between the portal vein and peripheral circulation, which is likely to modify lipoprotein metabolism.<br>To compare HDL apolipoprotein (apo) AI metabolism in patients treated with CSII and CIPII.	**HbA1c:** No change (CIPII: 7.24; CSII: 7.34 %, p=NS)	Strobe: 19/22<br>QAT:<br>Moderate: Data collection methods, study design, withdrawals and drop-outs<br>Poor: Selection bias, confounders

Legends: CSII, continuous subcutaneous insulin infusion; CIPII, continuous intraperitoneal insulin infusion; Pmc, Poor metabolic control; ND, no data available; NS, Not significant; BG, blood glucose; SMBG, self-monitored BG.

Table S2.1. (Continued)

	Source	Participant characteristics (Number, age (mean years), diabetes duration (mean years), sex (Male/Female), HbA1c (%), C-peptide, reasons to participate	Length of: CSII use, CSII follow-up, IPII follow-up (weeks)	Reported study objectives	Outcomes (mean, p-value)	Methodological quality
Glycaemic control	**Non-randomised crossover studies**				**Strengthening the Reporting of Observational Studies in Epidemiology (STROBE) and Thomas quality assessment toll (QAT):**	
	Liebl et al. 2013(conf. Abstracts/ Poster) [94-96]<br>Liebl et al. 2014 (c.poster) [97]	N = 12 (n = 10)*<br>Age: 49<br>Diabetes duration: 30<br>Sex: 2/10<br>HbA1c: 9.0 (8.8)*<br>C-peptide: ND<br>Reasons: Pmc	CSII use: ND<br>CIPII f-u: 104	To investigate the clinical long-term performance and safety of the new Accu-Chek DiaPort system.	**HbA1c:** Decreases (CIPII: M24*: 7.2, p=0.003; M12: 7.6, p=0.002; M6: 7.57, p<0.001; CSII: 9.0 %)<br>**BG (by CGM) > 10.0 mmol/l:** Decreases (CIPII: M6: 38: CSII: 53 %, p=0.036)<br>**BG (by CGM) in range 3.9 - 10.0 mmol/l:** Increases (CIPII: M6: 58; CSII: 45 %, p=0.027)<br>**Severe hypoglycaemia:** No change (CIPII: 3 events/24 months; CSII: 12 events/12 months, p=ND)	NP
	Dassau et al. 2017 [78]	N = 10<br>Age: 49<br>Diabetes duration: 29<br>Sex: 7/3<br>HbA1c: 7.7<br>C-peptide: ND<br>Reasons: Pmc	CSII use: 443<br>CSII f-u: 24h<br>CIPII f-u: 4 to 20<br>Washout: 4 to 20	To compare closed-loop zone MPC using the DiaPort IP insulin delivery system with the traditional SC insulin delivery method during a 24-hour in-clinic protocol.	**BG (by CGM)**: Decreases (CIPII: 8.3; CSII: 10.5 mmol/L, p=0.004)<br>**BG > 14 mmol/L:** Decreases (CIPII: 5.9; CSII: 23.0 %, p=0.0004)<br>**BG > 10mmol/L:** Decreases (CIPII: 32.4; CSII: 53.5 %, p=0.0014)<br>**BG in range 3.9 to 10 mmol/L:** Increases (CIPII: 65.7; CSII: 43.9 %, p=0.001)<br>**BG in range 4.4 to 7.8 mmol/L:** Increases (CIPII: 39.8; CSII: 25.6 %, p=0.03)<br>**BG < 3.8mmol/L:** No change (CIPII: 2.5; CSII: 4.1 %, p=0.42)	STROBE: 20/22<br>QAT:<br>Strong: Data collection methods, withdrawals and drop-outs, study design<br>Moderate: Selection bias, confounders
	**Retrospective crossover studies**					**STROBE and QAT:**
	Jeandidier et al. 1992 (Preliminary results) [86]	N = 8<br>Age: 33.5<br>Diabetes duration: 14.5<br>Sex: ND<br>HbA1c: 6.64<br>C-peptide: Neg<br>Reasons: ND	CSII use: 1<br>CIPII use: 12	To assess the potential benefits of CIPII vs SCII.	**HbA1c:** No change (CIPII: 6.7; CSII: 6.64 %, p=ND)<br>**SD of BG:** Decreases (CIPII: 3.3; CSII: 3.6 mmol/L/24h, p=0.038)<br>**Pre-prandial BG**: No change (CIPII: 7.2; CSII: 7.8 mmol/L, p=0.051)<br>**Post-prandial BG**: No change (CIPII: 8.7; CSII: 10.1 mmol/L, p=0.051)<br>**BG < 3.6 mmol/L**: No change (CIPII: 3.6; CSII: 4.0 events/week, p=ND)	STROBE: 12/22<br>QAT:<br>Weak: Study design<br>Unclear: Selection bias, confounders, data collection methods
	Catargi et al. 2002 [88]	N = 14<br>Age: 50.6<br>Diabetes duration: 28.0<br>Sex: 5/9<br>HbA1c: 7.8<br>C-peptide: Neg<br>Reasons: ND	CSII use: ND<br>CSII f-u: 6.4<br>Healing period: 6.4<br>CIPII f-u: 6.4[a]	To compare the efficacy of IPII and CSII of therapy in terms of glycaemic control, glycaemic stability and hypoglycaemia frequency.	**HbA1c:** Decreases (CIPII: 7.3; CSII: 7.8 %, p<0.05)<br>**Pre-prandial BG:** Decreases (CIPII: 7.8; CSII: 8.1 mmol/L, p<0.05)<br>**SMBG:** Decreases (CIPII: 8.0; CSII: 8.5 mmol/L, p<0.01)<br>**SD of BG:** Decreases (CIPII: 3.8; CSII: 4.4 mmol/L, p<0.01)<br>**Post-prandial BG:** No change (CIPII: 8.2; CSII: 8.5 mmol/L, p=0.07)	STROBE: 15/22<br>QAT:<br>Moderate: Study design, data collection method; withdrawals and drop-outs<br>Unclear: Selection bias, confounders

Legends: CSII, continuous subcutaneous insulin infusion; CIPII, continuous intraperitoneal insulin infusion; Pmc, Poor metabolic control; ND, no data available; NS, Not significant; BG, blood glucose; SMBG, self-monitored BG; CGM, continuous glucose monitoring; SD of BG, standard deviation of BG. Note, *, dropout in the study at 24months; [a], three patients first were treated with CIPII, and then with CSII.

Table S2.1. (Continued)

	Source	Participant characteristics (Number, age (mean years), diabetes duration (mean years), sex (Male/Female), HbA1c (%), C-peptide, reasons to participate	Length of: CSII use, CSII follow-up, IPII follow-up (weeks)	Reported study objectives	Outcomes (mean, p-value)	Methodological quality
Glycaemic control	**Case-control studies**					**Strengthening the Reporting of Observational Studies in Epidemiology (STROBE) and Thomas quality assessment toll (QAT):**
	Colette et al. 1989 [114]	N = 24 (CIPII: 13 /CSII: 11)<br>Age: 30/32<br>Diabetes duration: 17/20<br>Sex: ND<br>HbA1c: 8.0/8.9<br>C-peptide: ND<br>Reasons: ND	CSII use: 40<br>CIPII use: 60	Study the effects of prolonged tight diabetic control and insulin delivery through portal route on vitamin D metabolism in DM1.	**HbA1c:** No change (CIPII: 8.0; CSII: 8.9 %, p=NS)	STROBE: 18/22<br>QAT:<br>Strong: Data collection method<br>Moderate: Selection bias, study design, confounders
	Selam et al. 1989 [82]	N = 14 (CIPII: 6 /CSII: 8)<br>Age: 32/44.3<br>Diabetes duration: 16/23.1<br>Sex: 4/2 / 5/3<br>HbA1c: 8.3/8.7<br>C-peptide: ND<br>Reasons: ND	CSII use: 52+<br>CIPII use: 26	Compare the effects of intensive SC vs. implantable pump IP insulin delivery on intermediary metabolites in DM1 patients.	**HbA1c:** No change (CIPII: 8.2; CSII: 8.6 %, p=NS)<br>**Pre-prandial BG[FF]:** No change (CIPII: 7.3; CSII: 5.5 mmol/L, p=NS)<br>**Post-prandial BG**: No change (p=NS)	STROBE: 14/22<br>QAT:<br>Strong: Data collection methods<br>Moderate: Study design, confounders<br>Weak: Confounders<br>Unclear: Selection bias, blinding
	Walter et al. 1989 [99]	N = 12 (CIPII: 6 /CSII: 6)<br>Age: 28.3/26.6<br>Diabetes duration: 10.8/10.5<br>Sex: 6/0 / 6/0<br>HbA1c: 8.0/7.9<br>C-peptide: ND<br>Reasons: ND	CSII use: 26+<br>CIPII use: 12+	To compare metabolism control at night time in the patients with MDI and continuous insulin administration.	**HbA1c:** No change (CIPII: 8.0; CSII: 7.9 %, p=NS)	STROBE: 15/22<br>QAT:<br>Strong: Data collection methods<br>Moderate: Selection bias, study design, confounders<br>Unclear: Blinding<br>Not applicable: Withdrawals and drop-outs
	Hedman et al. 2009 (c.a) [111]<br>Arnqvist et al. 2010 (c.a.) [116]<br>Hedman et al. 2014 [112]	N = 30 (CIPII: 10 /CSII: 20)<br>Age: 53.1/52.8<br>Diabetes duration: 124.2/30.8<br>Sex: 5/5 / 10/10<br>HbA1c: 8.6/7.9<br>C-peptide: ND<br>Reasons: Pmc	CSII use: 26+<br>CIPII use: 26+	Investigate in cross-sectional study if the different modes of insulin administration, CIPII or CSII were associated with a change in the circulating IGF system.	**HbA1c:** No change (CIPII: 8.6; CSII: 7.9 %, p=NS)	STROBE: 21/22<br>QAT:<br>Strong: Selection bias, confounders, data collection method, withdrawals and drop-outs<br>Moderate: Study design

Legends: CSII, continuous subcutaneous insulin infusion; CIPII, continuous intraperitoneal insulin infusion; Pmc, Poor metabolic control; ND, no data available; NS, Not significant; BG, blood glucose; SMBG, self-monitored BG; SPAD, SC peritoneal access device; c.a., conference abstract; [FF], data extracted from figure.

Table S2.1. (Continued)

	Source	Participant characteristics (Number, age (mean years), diabetes duration (mean years), sex (Male/Female), HbA1c (%), C-peptide, reasons to participate	Length of: CSII use, CSII follow-up, IPII follow-up (weeks)	Reported study objectives	Outcomes (mean, p-value)	Methodological quality
Glycaemic control	**Case report**					**Critical appraisal tool of Center for Evidence-based management:**
	Catargi et al. 2000 [113]	N = 1<br>Age: 32<br>Diabetes duration: 6<br>Sex: 1/0<br>HbA1c: ND<br>C-peptide: Neg<br>Reasons: Pmc	CSII f-u (rapid -acting) (1): 12<br>CSII f-u (Lispro) (2): 3<br>CIPII use: 1.5+	To evaluate a new catheter design.	**HbA1c:** No change (CIPII: 5.9; CSII (1): 6.2; CSII (2): 6.1 %, p=ND)<br>**SMBG:** No change (CIPII: 6.3; CSII (1): 7.8; CSII (2): 7.3 mmol/L, p=ND)<br>**Pre-prandial BG:** No change (CIPII: 5.9; CSII (1): 6.4; CSII (2): 6.6 mmol/L, p=ND)<br>**Post-prandial BG:** No change (CIPII: 6.6; CSII (1): 9.6; CSII (2): 8.8 mmol/L, p=ND)<br>**LBGI^:** No change (CIPII: 4.3; CSII (1): 5.5; CSII (2): 4.0, p=ND)<br>**AUC (mean of 7 times/day SMBG):** No change (CIPII: 43.9; CSII (1): 49.5; CSII (2): 44.3 h.mmol/L, p=ND)	8/10 (2 cannot tell)

Legends: CSII, continuous subcutaneous insulin infusion; CIPII, continuous intraperitoneal insulin infusion; Pmc, Poor metabolic control; ND, no data available; BG, blood glucose; LBGI, low blood glucose index. Note, LBGI^ < 5, low or moderate risk of future severe hypoglycaemia; LBGI > 5, a high-risk; AUC, area under curve.

Table S2.2. Intervention studies, Participant characteristics, description, outcomes: Insulin levels

	Source	Participant characteristics (Number, age (mean years), diabetes duration (mean years), sex (Male/Female), HbA1c (%), C-peptide, reasons to participate	Length of: CSII use, CSII follow-up, IPII follow-up (weeks)	Reported study objectives	Outcomes (mean, p-value)	Methodological quality
Insulin levels	**Randomised crossover studies with wash-out period**					**Cochrane risk of bias tool (CRB):**
	Giacca et al. 1993 [100]	N = 5<br>Age: 31 - 50<br>Diabetes duration: 8 - 39<br>Sex: 1/4<br>HbA1c: 7.4<br>C-peptide: Neg<br>Reasons: Volunteers	CSII use: ND<br>CSII f-u: 96+ hours<br>CIPII f-u: 12+<br>Washout: serum free insulin level measurements after IV insulin bolus	To compare the rate of appearance of insulin in the peripheral circulation during IP and SC insulin administration in T1D, in steady and non-steady state.	**Fasting insulin levels**: Decreases (CIPII: 30.8; CSII: 45.0 pmol/L, p<0.001)<br>**Plasma clearance rate of insulin:** No change (CIPII: 14.7; CSII: 13.1 mL/kg*min, p=ND)<br>**Fasting recovery rate of insulin:** Decreases (CIPII: 27; CSII: 40 %, p<0.001)<br>**Insulin infusion 15 nmol/L for 150 min + 42nmol/L for another 150 min:** Increases recovery rate (with first increase (15nmol/h), p<0.05; with second increase (42nmol/h), p<0.01)<br>**Basal insulin requirement**: No change (CIPII: 5.4; CSII: 5.6 nmol/h, p=ND)	CRB:<br>Unclear risk of bias: Random sequence generation, allocation concealment, blinding<br>Low risk of bias: Incomplete outcome data, selective reporting, treatment procedure
	**Randomised follow-up studies**					**Cochrane risk of bias tool (CRB):**
	Liebl et al. 2009 [5]	N = 60[a] (CIPII: 30 /CSII: 30)<br>Age: 50.5/45.3<br>Diabetes duration: 26.3/25.1<br>Sex: (male) 73 %/43 %<br>HbA1c: 8.2/8.3<br>C-peptide: ND<br>Reasons: Pmc	CSII use: ND<br>CSII f-u: 26<br>CIPII f-u: 52	Comparison of frequency of hypoglycaemia, severe hypoglycaemia, metabolic control, diabetic QoL and safety between CSII and CIPII in type 1 diabetic patients.	**Mean daily insulin requirement**: No change (CIPII: 44.2; CSII: 46.0 U/24h, p=ND)	CRB:<br>Unclear risk of bias: Random sequence generation, allocation concealment, blinding<br>Low risk of bias: Incomplete outcome data, selective reporting, treatment procedure
	**Non-randomised crossover studies**					**Strengthening the Reporting of Observational Studies in Epidemiology (STROBE) and Thomas quality assessment toll (QAT):**
	Micossi et al. 1986 [84]	N = 6<br>Age: 38.8<br>Diabetes duration: 12.6<br>Sex: 3/3<br>HbA1c: 7.25<br>C-peptide: ≤ 0.02 pmol/mL<br>Reasons: Pmc	CSII use: 12<br>CSII f-u: 6<br>CIPII f-u: 6	To investigate the hormonal and metabolic patterns produced by CIPII in group of severely unstable DM1 who has previously responded poorly to CSII. To compare clinical and metabolic effects of CSII and CIPII.	**Mean daily insulin requirement**: No change (CIPII: 46.02; CSII: 48.67 U/24h, p=NS)	STROBE: 15/22<br>QAT:<br>Strong: Data collection methods, withdrawals and drop-outs<br>Moderate: Selection bias, study design<br>Weak: Confounders

Legends: CSII, continuous subcutaneous insulin infusion; CIPII, continuous intraperitoneal insulin infusion; Pmc, Poor metabolic control; ND, no data available; NS, Not significant; [a], dropouts in this study (at the end of the periods N = 36 (CIPII: 15 /CSII: 21).

Table S2.2. (Continued)

	Source	Participant characteristics (Number, age (mean years), diabetes duration (mean years), sex (Male/Female), HbA1c (%), C-peptide, reasons to participate	Length of: CSII use, CSII follow-up, IPII follow-up (weeks)	Reported study objectives	Outcomes (mean, p-value)	Methodological quality
Insulin levels	**Non-randomised crossover studies**					**Strengthening the Reporting of Observational Studies in Epidemiology (STROBE) and Thomas quality assessment toll (QAT):**
	Beylot et al. 1987 [103]	N = 4<br>Age: 42<br>Diabetes duration: 21.5<br>Sex: 3/1<br>HbA1c: 7.6 (9.2 – 5)<br>C-peptide: ND<br>Reasons: Volunteers	CSII use: ND<br>CSII f-u: 8<br>CIPII f-u: 8<br>Washout: 1 day	To determine if IP insulin administration could, in addition to decreasing peripheral insulin levels, improve the insulin resistance of DM1.	**Fasting insulin levels**: No change (CIPII: 131.95; CSII: 152.79 ρmol/L, p=ND)<br>**Plasma free insulin (night-time):** Decreases (CIPII: 127.78; CSII: 163.2 ρmol/L, p<0.05),<br>**Mean daily insulin requirement** [DT]: No change (CIPII: 0.0.57; CSII: 0.0.59 U/kg/day, p=ND)	STROBE: 15/22<br>QAT:<br>Strong: Blinding, data collection methods, withdrawals and drop-outs<br>Moderate: Selection bias, study design, confounders
	Wredling, Lui et al. 1991 [92]	N = 6<br>Age: 42.8<br>Diabetes duration: 24.0<br>Sex: 4/2<br>HbA1c: 7.7 – 10.2<br>C-peptide: Neg<br>Reasons: Pmc	CSII use: ND<br>CSII f-u: 208<br>CIPII f-u: 38	To compare the reproducibility of the plasma-insulin profile of IP and SC administered insulin in a group of C-peptide-negative, diabetic patients.	**Pre-meal insulin bolus (time till max. conc.):** Decreases (CIPII: 60; CSII: 133 minutes, p=0.006)<br>**Total insulin AUC (0-240 minutes)**: No change (CIPII (bolus 0.05 U/kg/BW): 56.1 mU; CSII (bolus 0.1 U/kg/BW): 94.6 mU, p=0.0023)<br>**Insulin AUC 0-60 min**: No change (CIPII: 16.3; CSII: 20.6 mU, p=NS)<br>**Intra-patient CV (AUC 0-60 min):** No change (CIPII: 19.8; CSII: 38.6 %, p=NS)<br>**Intra-patient CV (AUC 0-240 min):** No change (CIPII: 11.5; CSII: 20.2 %, p=NS)<br>**Inter-patient peak time:** No change (CIPII: 22.4; CSII: 28.3 %, p=NS)<br>**Inter-patient CV (AUC 0-60 min):** No change (CIPII: 43.6; CSII: 27.9 %, p=NS)<br>**Inter-patient CV (AUC 0-240 min):** No change (CIPII: 30.9; CSII: 29.7 %, p=NS)<br>**Inter-patient peak time:** No change (CIPII: 44.0; CSII: 28.0 %, p=NS)	STROBE: 15/22<br>QAT:<br>Strong: Data collection method<br>Moderate: Study design<br>Weak: Selection bias<br>Unclear: Confounders<br>Not applicable: Withdrawals and drop-outs
	Wredling, Adamson et al. 1991 (Technical report) [91]	N = 6<br>Age: 41.3<br>Diabetes duration: 23.2<br>Sex: 4/2<br>HbA1c: 8.7<br>C-peptide: Neg<br>Reasons: Pmc	CSII use: 52+<br>CSII f-u: 8 (n=3)<br>CIPII f-u: median 72	To determine the efficacy of a new percutaneous device.	**Mean daily insulin requirement**: No change (CIPII: 44.8 U/24h; CSII: ND)	STROBE:15/22<br>QAT:<br>Moderate: Selection bias, study design, data collection method<br>Weak: Withdrawals and drop-outs<br>Unclear: Confounders

Legends: CSII, continuous subcutaneous insulin infusion; CIPII, continuous intraperitoneal insulin infusion; Pmc, Poor metabolic control; ND, no data available; NS, Not significant; CV, coefficient of variation; AUC, area under curve; [DT], data calculated from table.

Table S2.2. (Continued)

	Source	Participant characteristics (Number, age (mean years), diabetes duration (mean years), sex (Male/Female), HbA1c (%), C-peptide, reasons to participate	Length of: CSII use, CSII follow-up, IPII follow-up (weeks)	Reported study objectives	Outcomes (mean, p-value)	Methodological quality
Insulin levels	**Non-randomised crossover studies**				**Strengthening the Reporting of Observational Studies in Epidemiology (STROBE) and Thomas quality assessment toll (QAT):**	
	Georgopoulos et al. 1992 [83]	N = 7<br>Age: 27<br>Diabetes duration: 12<br>Sex: 5/2<br>HbA1c: 9.8<br>C-peptide: ND<br>Reasons: ND	CSII use: ND<br>CIPII f-u: 52-60	To investigate whether long-term improved glycaemic control by intraperitoneal insulin infusion normalizes the compositional abnormalities of triglyceride (TG)-rich lipoproteins in DM1.	**Mean daily insulin requirement**: No change (CIPII: 57.2; CSII: 52 (units of measurements are not provided, p=NS)	STROBE: 11/22<br>QAT:<br>Strong: Data collection methods, withdrawals and drop-puts<br>Moderate: Selection bias, study design, confounders
	Georgopoulos et al. 1994 [102]	N = 8<br>Age: 37<br>Diabetes duration: 21.6<br>Sex: 5/3<br>HbA1c: 9.4<br>C-peptide: ND<br>Reasons: ND	CSII use: ND<br>CIPII f-u: 26	Test hypothesis that IPII will decrease the level of circulating chylomicron remnants in patients with DM1.	**Mean daily insulin requirement**: No change (CIPII: 62.4; CSII: 61.9 U/24h, p=NS)	STROBE: 14/22<br>QAT:<br>Strong: Data collection method, withdrawals and dropouts<br>Moderate: Study design, confounders<br>Unclear: Selection bias
	Lassmann-Vague et al. 1994 (short communicati on) [104]	N = 11<br>Age: 34.4<br>Diabetes duration: 22.4<br>Sex: 5/6<br>HbA1c: 6.9<br>C-peptide: Neg<br>Reasons: ND	CSII use: 26+<br>CSII f-u: 4<br>CIPII f-u: 12	ND	**Fasting insulin levels:** No change (CIPII: M1: 111.12; M3: 114.59; CSII: 118.06 pmol/L, p=ND)<br>**Mean daily insulin requirement**: No change (CIPII: 41.6; CSII: 40.5 U/24h, p=ND)	NP
	Raccah et al. 1994 (letter) [109]	N = 11<br>Age: 34.4<br>Diabetes duration: 22.3<br>Sex: 6/5<br>HbA1c: 6.9<br>C-peptide: ND<br>Reasons: ND	CSII use: 12<br>CIPII f-u: 40	ND	**Fasting insulin levels:** No change (CIPII: M3: 114.59; M10: 100; CSII: 118.06 pmol/L, p=NS)<br>**Mean daily insulin requirement**: No change (CIPII: 62.4; CSII: 40.5 U/24h, p=NS)	NP
	Schnell et al. 1994 [105]	N = 5<br>Age: 25-62<br>Diabetes duration: 20.2<br>Sex: 1/4<br>HbA1c: 9.8<br>C-peptide: ND<br>Reasons: ND	CSII use: 156-364<br>CIPII f-u: 52	To compare insulin demands during 24 h in CIPII and CSII patients.<br>To compare HbA1c levels in CIPII and CSII patients.	**Mean daily insulin requirement:** No change (CIPII: 46; CSII: 48 U/24h, p=NS)	STROBE: 17/22<br>QAT:<br>Strong: Withdrawals and drop-outs<br>Moderate: Selection bias, study design, confounders, data collection method

Legends: CSII, continuous subcutaneous insulin infusion; CIPII, continuous intraperitoneal insulin infusion; ND, no data available; NS, Not significant; NP, not possible to evaluate.

Table S2.2. (Continued)

	Source	Participant characteristics (Number, age (mean years), diabetes duration (mean years), sex (Male/Female), HbA1c (%), C-peptide, reasons to participate	Length of: CSII use, CSII follow-up, IPII follow-up (weeks)	Reported study objectives	Outcomes (mean, p-value)	Methodological quality
Insulin levels	**Non-randomised crossover studies**				**Strengthening the Reporting of Observational Studies in Epidemiology (STROBE) and Thomas quality assessment toll (QAT):**	
	Guerci et al. 1996 [108]	N = 14<br>Age: 40.0<br>Diabetes duration: 16.4<br>Sex: 9/5<br>HbA1c: 6.1<br>C-peptide: Neg<br>Reasons: Volunteers	CSII use: 52+<br>CIPII f-u: 16	To determine the effects of IPII on qualitative lipoprotein abnormality.	**Mean daily insulin requirement:** No change (CIPII: M2: 0.69, p<0.01; M4: 0.64; CSII: 0.60 U/kg/24h, p=NS)	STROBE: 16/22<br>QAT:<br>Strong: Selection bias, confounders, data collection method, withdrawals and drop-outs<br>Moderate: Study design
	Hanaire-Broutin et al. 1996 [101]	N = 18<br>Age: 43.0<br>Diabetes duration: 20.0<br>Sex: 11/7<br>HbA1c: 7.6<br>C-peptide: Neg<br>Reasons: Volunteers	CSII use: 128<br>CIPII f-u: 52	To evaluate the impact of intraperitoneal insulin therapy, which results in preferential insulin absorption by the portal system, on the hepatic growth hormone-resistant state of DM1.	**Mean daily insulin requirement:** No change (CIPII: 39.4; CSII: 39.1 U/24h, p=NS)	STROBE: 16/22<br>QAT:<br>Strong: Study design, data collection methods, withdrawals and drop-outs<br>Moderate: Selection bias, confounders
	Lassmann-Vague et al. 1996 [101]	N = 11<br>Age: 36.3<br>Diabetes duration: 17.8<br>Sex: 6/5<br>HbA1c: ND<br>C-peptide: ND<br>Reasons: ND	CSII use: ND<br>CSII f-u: ND<br>CIPII f-u: 8	To compare plasma free insulin levels achieved in patients with DM1 chronically treated with CSII and CIPII.	**Fasting insulin levels** (7:00 am): No change (CIPII: 60.42; CSII: 66.67 pmol/L, p=NS)<br>**Plasma free insulin (night-time (12:00 am)):** Decreases (CIPII: 70.15; CSII: 128.48 pmol/L, p<0.01)<br>**Pre-meal insulin bolus (time till max conc.):** Decreases (CIPII: 1 h; CSII: 3 h, p<0.05)<br>**(max. insulin conc.):** Increases (CIPII: 179.18; CSII: 125.01 pmol/L, p<0.05)<br>**elevation (return to basal concentration)**: Decreases (CIPII: 3 h; CSII: did not return till next bolus)<br>**Total insulin AUC**: Decreases (CIPII: 72; CSII: 100 mU/h/L, p<0.01)<br>**Night-time AUC:** Decreases (CIPII: 12; CSII: 36 mU/L/h, p<0.01)<br>**AUC after insulin bolus:** No change (CIPII: 32; CSII: 30 mU/L/h, p=NS)<br>**Mean daily insulin requirement:** No change (1.3 U/h)	STROBE: 14/22<br>QAT:<br>Strong: Data collection method, withdrawals and drop-outs<br>Moderate: Selection bias, study design<br>Weak: Confounders
	Pacifico et al. 1997 [98]	N = 8<br>Age: 35.1<br>Diabetes duration: 19<br>Sex: 5/4<br>HbA1c: 6.5<br>C-peptide: Neg<br>Reasons: Volunteers	CSII use: 12+<br>CIPII f-u: 52+	To evaluate the safety, the efficacy and the results after 3 years of CIPII.	**Mean daily insulin requiremen**t: No change (CIPII: 42.8; CSII: 40.8 U/24h, p=NS)	STROBE:19/22<br>QAT:<br>Strong: Study design, data collection methods, Selection bias<br>Moderate: Confounders, withdrawals and drop-outs

Legends: CSII, continuous subcutaneous insulin infusion; CIPII, continuous intraperitoneal insulin infusion; ND, no data available; NS, Not significant; NP, not possible to evaluate; AUC, area under curve.

Table S2.2. (Continued)

	Source	Participant characteristics (Number, age (mean years), diabetes duration (mean years), sex (Male/Female), HbA1c (%), C-peptide, reasons to participate	Length of: CSII use, CSII follow-up, IPII follow-up (weeks)	Reported study objectives	Outcomes (mean, p-value)	Methodological quality
Insulin levels	**Non-randomised crossover studies**				**Strengthening the Reporting of Observational Studies in Epidemiology (STROBE) and Thomas quality assessment toll (QAT):**	
	Oskarsson et al. 1999 [90]	N = 7<br>Age: 42<br>Diabetes duration: 15<br>Sex: 5/2<br>HbA1c: 8.5<br>C-peptide: < 0.2nM<br>Reasons: Pmc	CSII use: 26+<br>CIPII f-u: 47-82	To assess the clinical relevance of the BG, hypoglycaemia, glucagon secretion during exercise by comparing glycaemic and hormonal responses to a 40-min bicycle exercise test at 60 % of $VO_{2\ max}$ during CSII and CIPII in type 1 diabetic patients.	**Fasting insulin levels:** decreases (CIPII: 28.0; CSII: 48.1 pmol/L, p=0.043)<br>**Change in insulin levels during the time of exercises[FF]:** No change (in the groups); increases (between groups, through the study, p<0.05)<br>**Mean daily insulin requirement:** No change (CIPII: 38.4; CSII: 36.1 U/24h, p=0.06)	STROBE:16/22<br>QAT:<br>Strong: Confounders, data collection methods, withdrawals and drop-outs<br>Moderate: Selection bias, study design
	Oskarsson et al. 2000 [89]	N = 6<br>Age: 42<br>Diabetes duration: 17<br>Sex: 5/2<br>HbA1c: 8.6<br>C-peptide: Neg<br>Reasons: Unsatisfactory on CSII	CSII use: 52+<br>CIPII f-u: 69	To expose the patients to an identical hyperinsulinemic challenge with special emphasis on the glucagon response in the same patients during continuous treatment with CSII and CIPII.	**Fasting insulin levels:** Decreases (CIPII: 35.8; CSII: 53.4 pmol/L, p<0.01)<br>**Change in plasma hormone levels from basal level to peak level in time of insulin clamp; and change between CIPII and CSII:**<br>**Insulin(+30 min):** Increases in both (CIPII: 66.9, p=0.01; CSII: 42.4 pmol/L, p=0.03); No change (p=0.32)<br>**Basal rate:** Increases (CIPII: 1.34; CSII: 1.14 U/h, p=0.02)<br>**Bolus doses:** Decreases (CIPII: 7.1; CSII: 11.6 U/24h, p=0.04)<br>**Mean daily insulin requirement:** No change (CIPII: 37.9; CSII: 38.2 U/24h, p=0.95)	STROBE: 16/22<br>QAT:<br>Strong: Confounders, data collection methods, withdrawals and drop-outs<br>Moderate: Selection bias, study design
	Duvillard et al. 2005 (Brief report) [106]<br>Duvillard et al 2007 [107]	N = 7<br>Age: 48<br>Diabetes duration: 17<br>Sex: 6/1<br>HbA1c: 7.34<br>C-peptide: ND<br>Reasons: ND	CSII use: ND<br>CIPII f-u: 12	Compare if replacement of SCII with IPII restores the normal physiological gradient between the portal vein and peripheral circulation, which is likely to modify lipoprotein metabolism.<br>To compare HDL apolipoprotein (apo) AI metabolism in patients treated with CSII and IPII.	**Mean daily insulin requirement:** No change (CIPII: 43.6; CSII: 45.0 U/24h, p=0.69)	Strobe: 19/22<br>QAT:<br>Moderate: Data collection methods, study design, withdrawals and drop-outs<br>Poor: Selection bias, confounders
	Liebl et al. 2013 (c.a) [94-96]<br>Liebl et al 2014 (c.a) [97]	N = 12 (n = 10)*<br>Age: 49<br>Diabetes duration: 30<br>Sex: 2/10<br>HbA1c: 9.0 (8.8)*<br>C-peptide: ND<br>Reasons: Pmc	CSII use: ND<br>CIPII f-u: 104	To investigate the clinical long-term performance and safety of the new Accu-Chek DiaPort system.	**Mean daily insulin requirement:** No change (CIPII: M6: 45; CSII: 49 U, p=NS)	NP

Legends: CSII, continuous subcutaneous insulin infusion; CIPII, continuous intraperitoneal insulin infusion; ND, no data available; NS, Not significant; [FF], data extracted from figure; *, dropouts in the study; Pmc, Poor metabolic control.

Table S2.2. (Continued)

	Source	Participant characteristics (Number, age (mean years), diabetes duration (mean years), sex (Male/Female), HbA1c (%), C-peptide, reasons to participate	Length of: CSII use, CSII follow-up, IPII follow-up (weeks)	Reported study objectives	Outcomes (mean, p-value)	Methodological quality
Insulin levels	**Non-randomised crossover studies**					**Strengthening the Reporting of Observational Studies in Epidemiology (STROBE) and Thomas quality assessment toll (QAT):**
	Dassau et al. 2017 [78]	N = 10<br>Age: 49<br>Diabetes duration: 29<br>Sex: 7/3<br>HbA1c: 7.7<br>C-peptide: ND<br>Reasons: Pmc	CSII use: 443<br>CSII f-u: 24h<br>CIPII f-u: 4 to 20	To compare closed-loop zone MPC using the DiaPort IP insulin delivery system with the traditional SC insulin delivery method during a 24-hour in-clinic protocol.	**In in-clinical measurements: 24-hour total insulin delivery:** Increases (CIPII: 43.66; CSII: 32.29 U, p<0.001)<br>**Mean daily insulin requirement**: No change (CIPII: ND; CSII: 43 U/24h)	STROBE: 20/22<br>QAT:<br>Strong: Data collection methods, withdrawals and drop-outs, study design<br>Moderate: Selection bias, confounders
	**Retrospective crossover studies**					**Strengthening the Reporting of Observational Studies in Epidemiology (STROBE) and Thomas quality assessment toll (QAT):**
	Jeandidier et al. 1992 (Preliminary results) [86]	N = 8<br>Age: 33.5<br>Diabetes duration: 14.5<br>Sex: ND<br>HbA1c: 6.64<br>C-peptide: Neg<br>Reasons: ND	CSII use: 1<br>CIPII use: 12	To assess the potential benefits of CIPII vs SCII.	**Mean daily insulin requirement**: Increase (CIPII: 39; CSII: 32 U/24h, p<0.05)	STROBE: 12/22<br>QAT:<br>Weak: Study design<br>Unclear: Selection bias, confounders, data collection methods
	**Non-randomised follow-up studies**					**STROBE and QAT:**
	Van Dijk et al. 2016 [93]	N = 101 (CIPII: 32 /CSII: 69)[b]<br>Age: 50/48<br>Diabetes duration: 29/27<br>Sex: 14/25 / 30/44<br>HbA1c: 8.3/7.9<br>C-peptide: ND<br>Reasons: Pmc	CSII/MDI use: 208+<br>CIPII use: 208+<br>CSII f-u: 27<br>CIPII f-u: 27	To compare the effects of CIPII to SC insulin therapy, on the GH-IGF-1 axis in a large prospective, observational matched case-control study in T1DM patients.	**Mean daily insulin requirement**: No change (CIPII: 0.7; CSII: 0.6 U/24h/kg, p=NS)	STROBE: 16/22<br>QAT:<br>Strong: Selection bias, study design, data collection method<br>Moderate: Study design, withdrawals and drop-outs
	**Case-control studies**					**STROBE and QAT:**
	Colette et al. 1989 [114]	N = 24 (CIPII: 13 /CSII: 11)<br>Age: 30/32<br>Diabetes duration: 17/20<br>Sex: ND<br>HbA1c: 8.0/8.9<br>C-peptide: ND<br>Reasons: ND	CSII use: 40<br>CIPII use: 60	Study the effects of prolonged tight diabetic control and insulin delivery through portal route on vitamin D metabolism in insulin dependent diabetic patients.	**Fasting insulin levels:** No change (CIPII: 115.28; CSII: 140.98 pmol/L, p=NS)	STROBE: 18/22<br>QAT:<br>Strong: Data collection method, withdrawals and drop-outs<br>Moderate: Selection bias, study design, confounders

Legends: CSII, continuous subcutaneous insulin infusion; CIPII, continuous intraperitoneal insulin infusion; ND, no data available; NS, Not significant; Pmc, Poor metabolic control; c.a, conference abstract. Note: [b], for analysis participant nr. changed (dropouts).

Table S2.2. (Continued)

	Source	Participant characteristics (Number, age (mean years), diabetes duration (mean years), sex (Male/Female), HbA1c (%), C-peptide, reasons to participate	Length of: CSII use, CSII follow-up, IPII follow-up (weeks)	Reported study objectives	Outcomes (mean, p-value)	Methodological quality
Insulin levels	**Case-control studies**					**Strengthening the Reporting of Observational Studies in Epidemiology (STROBE) and Thomas quality assessment toll (QAT):**
	Selam et al.1989 [82]	N = 14 (CIPII: 6 /CSII: 8)<br>Age: 32/44.3<br>Diabetes duration: 16/23.1<br>Sex: 4/2 / 5/3<br>HbA1c: 8.3/8.7<br>C-peptide: ND<br>Reasons: ND	CSII use: 52+<br>CIPII use: 26	Compare the effects of intensive SC vs. implantable pump IP insulin delivery on intermediary metabolites in DM1 patients.	**Fasting insulin levels** [FF]: No change (NS)<br>**Pre-meal insulin bolus (bolus + 4 h basal rate = 0.15 U/kg) (time till max conc.):** No change (CIPII: 30 min; CSII: 60 min, p=ND)<br>**(max. insulin conc.):** Increases (CIPII: 263.91; CSII: 145.84 pmol/L) (at +30 min, p<0.05);<br>**elevation (return to basal concentration:** Decreases (CIPII: 180; CSII: 240 minutes, p=ND).	STROBE: 14/22<br>QAT:<br>Strong: Data collection methods<br>Moderate: Study design, confounders<br>Weak: Confounders<br>Unclear: Selection bias, blinding<br>Not applicable: Withdrawals and drop-outs
	Walter et al. 1989 [99]	N = 12 (CIPII: 6 /CSII: 6)<br>Age: 28.3/26.6<br>Diabetes duration: 10.8/10.5<br>Sex: 6/0 / 6/0<br>HbA1c: 8.0/7.9<br>C-peptide: ND<br>Reasons: ND	CSII use: 26+<br>CIPII use: 12+	To compare metabolism control at night time in the patients with ICT and continuous insulin administration.	**Mean night insulin values (At night (23:00–7:00)):** Decreases (CIPII: 65.56; CSII: 86.53 pmol/L, p<0.005).<br>**Mean daily insulin requirement**: No change (CIPII: 0.56; CSII: 0.55 U/kg/24h, p=NS)	STROBE: 15/22<br>QAT:<br>Strong: Data collection methods<br>Moderate: Selection bias, study design, confounders<br>Unclear: Blinding<br>Not applicable: Withdrawals and drop-outs
	Hedman et al. 2009 (poster) [111]<br>Arnqvist et al. 2010 (poster) [116]<br>Hedman et al. 2014 [112]	N = 30 (CIPII: 10 /CSII: 20)<br>Age: 53.1/52.8<br>Diabetes duration: 124.2/30.8<br>Sex: 5/5 / 10/10<br>HbA1c: 8.6/7.9<br>C-peptide: ND<br>Reasons: Pmc	CSII use: 26+<br>CIPII use: 26+	Investigate in cross-sectional study if the different modes of insulin administration, CIPII or CSII were associated with a change in the circulating IGF system.	**Mean daily insulin requirement**: No change (CIPII: 51.2; CSII: 39.3 U/24h, p=0.260)	STROBE: 21/22<br>QAT:<br>Strong: Selection bias, confounders, data collection method, withdrawals and drop-outs<br>Moderate: Study design
	**Case report**					**Critical appraisal tool of Centre for Evidence-based management:**
	Catargi et al. 2000 [113]	N = 1<br>Age: 32<br>Diabetes duration: 6<br>Sex: 1/0<br>HbA1c: ND<br>C-peptide: Neg<br>Reasons: Pmc	CSII f-u: (rapid-acting insulin) (1): 12<br>CSII f-u (Lispro): 12<br>CIPII: 1.5+	To evaluate a new catheter design	**Mean daily insulin requirement**: No change (CIPII: 52; CSII (1): 51.2; CSII (2): 50.9, p=ND)	8/10 (2 cannot tell)

Legends: CSII, continuous subcutaneous insulin infusion; CIPII, continuous intraperitoneal insulin infusion; Pmc, Poor metabolic control; ND, no data available; NS, Not significant; [FF], data extracted from figure.

Table S2.3. Intervention studies, Participant characteristics, description, outcomes: Intermediate metabolites

	Source	Participant characteristics (Number, age (mean years), diabetes duration (mean years), sex (Male/Female), HbA1c (%), C-peptide, reasons to participate	Length of: CSII use, CSII follow-up, IPII follow-up (weeks)	Reported study objectives	Outcomes (mean, p-value)	Methodological quality
Secondary outcomes: Intermediate metabolites	**Non-randomised crossover studies**				**Strengthening the Reporting of Observational Studies in Epidemiology (STROBE) and Thomas quality assessment toll (QAT):**	
	Micossi et al. 1986 [84]	N = 6<br>Age: 38.8<br>Diabetes duration: 12.6<br>Sex: 3/3<br>HbA1c: 7.25<br>C-peptide: ≤ 0.02 pmol/mL<br>Reasons: Poor glucose control	CSII use: 12<br>CSII f-u: 6<br>CIPII f-u: 6	To investigate the hormonal and metabolic patterns produced by CIPII in group of severely unstable DM1 who has previously responded poorly to CSII. To compare clinical and metabolic effects of CSII and CIPII.	**Total cholesterol**: No change (CIPII: 5.1; CSII: 4.4 mmol/L, p=NS)<br>**HDL-cholesterol:** Decreases (CIPII: 1.2; CSII: 1.4 mmol/L, p<0.05)<br>**HDL_2 cholesterol**: Decreases (CIPII: 0.3; CSII: 0.6 mmol/L, p<0.01)<br>**HDL_3 cholesterol**: No change (CIPII: 0.95; CSII: 0.9 mmol/L, p=NS)<br>**Fasting serum triglycerides**: Increases (CIPII: 1.5; CSII: 0.9 mmol/L, p<0.005)<br>**Mean daily glycerol**: No change (CIPII: 61.7; CSII: 35.4 µmol/L, p=NS)	STROBE: 15/22<br>QAT:<br>Strong: Data collection methods, withdrawals and drop-outs<br>Moderate: Selection bias, study design<br>Weak: Confounders
	Georgopoulos et al. 1992 [83]	N = 7<br>Age: 27<br>Diabetes duration: 12<br>Sex: 5/2<br>HbA1c: 9.8<br>C-peptide: ND<br>Reasons: ND	CSII use: ND<br>CIPII f-u: 52-60	To investigate whether long-term improved glycaemic control by intraperitoneal insulin infusion normalizes the compositional abnormalities of triglyceride (TG)-rich lipoproteins in DM1.	**Total cholesterol:** No change (CIPII: 4.6; CSII: 4.9 mmol/L, p=NS)<br>**HDL cholesterol:** No change (CIPII: 1.30; CSII: 1.33 mmol/L, p=NS)<br>**Fasting plasma triglyceride:** No change (CIPII: 1.23; CSII: 1.35 mmol/L, p=NS)<br>**Differences after fat ingestion:** Plasma TG increased in both groups (no statistically significant changes in any time point).<br>**Mean ratios of constituents in fasting lipoprotein mass:**<br>**Total cholesterol-triglyceride:** **Sf 100-400:** CIPII: 0.20; CSII: 0.29, p<0.008; **Sf 20-100:** CIPII: 0.375; CSII: 0.483, p<0.01<br>**Total cholesterol-phospholipid:** Sf 100-400: CIPII: 0.594; CSII: 0.975, p<0.001; Sf 20-100: CIPII: 0.73; CSII: 1.295, p<0.004<br>**Lipid-protein:** Sf 100-400: CIPII: 14.07; CSII: 13.93, p=NS; Sf 20-100: CIPII: 10.16; CSII: 10.92, p=NS	STROBE: 11/22<br>QAT:<br>Strong: Data collection methods, withdrawals and drop-puts<br>Moderate: Selection bias, study design, confounders
	Raccah et al. 1994 (letter) [109]	N = 11<br>Age: 34.4<br>Diabetes duration: 22.3<br>Sex: 6/5<br>HbA1c: 6.9<br>C-peptide: ND<br>Reasons: ND	CSII use: 12<br>CIPII f-u: 40	ND	**Total cholesterol:** No change (CIPII: M3: 4.74; M10: 4.92; CSII: 5.03 mmol/L, p=NS)<br>**Fasting plasma triglycerides:** No change (CIPII: M3: 0.88; M10: 0.83; CSII: 0.83 mmol/L, p=NS)	NP

Legends: CSII, continuous subcutaneous insulin infusion; CIPII, continuous intraperitoneal insulin infusion; ND, no data available; NS, Not significant; NP, not possible to evaluate; TG, triglycerides; FFA, free fatty acids; HDL, high density lipoprotein; LDL, low density lipoprotein.

Table S2.3. (Continued)

	Source	Participant characteristics (Number, age (mean years), diabetes duration (mean years), sex (Male/Female), HbA1c (%), C-peptide, reasons to participate	Length of: CSII use, CSII follow-up, IPII follow-up (weeks)	Reported study objectives	Outcomes (mean, p-value)	Methodological quality
**Secondary outcomes: Intermediate metabolites**	**Non-randomised crossover studies**				**Strengthening the Reporting of Observational Studies in Epidemiology (STROBE) and Thomas quality assessment toll (QAT):**	
	Georgopoulos et al. 1994 [102]	N = 8<br>Age: 37<br>Diabetes duration: 21.6<br>Sex: 5/3<br>HbA1c: 9.4<br>C-peptide: ND<br>Reasons: ND	CSII use: ND<br>CIPII f-u: 26	Test hypothesis that IPII will decrease the level of circulating chylomicron remnants in patients with DM1.	**Fasting:**<br>**Total cholesterol:** Decreases (CIPII: 4.56; CSII: 4.85 mmol/L, p=0.044)<br>**HDL cholesterol:** No change (CIPII: 1.26; CSII: 1.30 mmol/L, p=NS)<br>**LDL cholesterol:** No change (CIPII: 2.87; CSII: 3.10 mmol/L, p=NS)<br>**Plasma triglycerides:** No change (CIPII: 0.93; CSII: 0.93 mmol/L, p=NS)<br>**Differences after fat ingestion [FF]:**<br>**Max. conc. TG: Sf. > 100:** No change (follows similar pattern) (CIPII: 0.6; CSII: 0.7 mmol/L, p=NS)<br>**Time till TG Sf > 100 max conc.:** No change (follows similar pattern) (CIPII: 4; CSII: 4 hours, p=NS)<br>**Plasma TG Sf. 20-100:** No change (follows similar pattern) (p=NS)<br>**ApoB: Sf. > 100:** No change (follows similar pattern) (p=NS)<br>**ApoB Sf. 20-100:** No change (p=NS)<br>**Retinyl esters Sf > 100:** Decreases (+4 hours: CIPII: 2500; CSII: 6000 µg/L, p=0.05)<br>**Retinyl esters Sf 20-100:** No change (follows similar pattern) decreases (+ 8 hours; CIPII: 450; CSII: 700 µg/L, p=0.075)<br>**Retinyl ester: apoB ratio: (S_f > 100)**: Decreases (p=0.0002)<br>**S_f 60-100:** No change (p=0.06)	STROBE: 14/22<br>QAT:<br>Strong: Data collection method, withdrawals and dropouts<br>Moderate: Study design, confounders<br>Unclear: Selection bias
	Guerci et al. 1996 [108]	N = 14<br>Age: 40.0<br>Diabetes duration: 16.4<br>Sex: 9/5<br>HbA1c: 6.1<br>C-peptide: Neg<br>Reasons: Volunteers	CSII use: 52+<br>CIPII f-u: 16	To determine the effects of IPII on qualitative lipoprotein abnormality.	**Fasting:**<br>**Total cholesterol**: No change (CIPII: 5.01; CSII: 4.97 mmol/L, p=NS)<br>**HDL cholesterol:** No change (CIPII: 1.49; CSII: 1.57 mmol/L, p=NS)<br>**LDL cholesterol:** No change (CIPII: 1.49; CSII: 1.57 mmol/L, p=NS)<br>**Plasma triglyceride:** No change (CIPII: 1.13; CSII: 1.1 mmol/L, p=NS)<br>**Total plasma lipids:** No change (CIPII: 3.02; CSII: 2.95 mmol/L, p=NS)<br>**Apo A-I:** No change (CIPII: 3.96; CSII: 4.06 mmol/L, p=NS)<br>**Apo B:** No change (CIPII: 2.56; CSII: 2.46 mmol/L, p=NS)<br>**Lp B-PL:** Increases (CIPII: 1.36; CSII: 1.09 mmol/L, p<0.01)<br>**Lp B-PL/apo B:** Increases (CIPII: 1.39; CSII: 1.17 mmol/L, p<0.05)<br>**Lp B-TC:** No change (CIPII: 3.51; CSII: 3.35 mmol/L, p=NS)<br>**Lp no B-PL:** No change (CIPII: 1.75; CSII: 1.88 mmol/L, p=NS)<br>**Lp no B-TC:** No change (CIPII: 1.50; CSII: 1.62 mmol/L, p=NS)	STROBE: 16/22<br>QAT:<br>Strong: Selection bias, confounders, data collection method, withdrawals and drop-outs<br>Moderate: Study design

Legends: CSII, continuous subcutaneous insulin infusion; CIPII, continuous intraperitoneal insulin infusion; ND, no data available; NS, not significant; HDL, high density lipoprotein; LDL, low density lipoprotein; LpB, Apo B-containing lipoprotein particles; LP no B, no-apo-B containing particles; Sf, lipoprotein size; TC, total cholesterol; PL, plasma lipids; VLDL, very-low-density lipoproteins; [FF], data extracted from figure. Note: Retinyl esters – a marker of intestinal lipoproteins.

Table S2.3. (Continued)

	Source	Participant characteristics (Number, age (mean years), diabetes duration (mean years), sex (Male/Female), HbA1c (%), C-peptide, reasons to participate	Length of: CSII use, CSII follow-up, IPII follow-up (weeks)	Reported study objectives	Outcomes (mean, p-value)	Methodological quality
**Secondary outcomes: Intermediate metabolites**	**Non-randomised crossover studies**				**Strengthening the Reporting of Observational Studies in Epidemiology (STROBE) and Thomas quality assessment toll (QAT):**	
	Pacifico et al. 1997 [98]	N = 8<br>Age: 35.1<br>Diabetes duration: 19<br>Sex: 5/4<br>HbA1c: 6.5<br>C-peptide: Neg<br>Reasons: Volunteers	CSII use: 12+<br>CIPII f-u: 52+	To evaluate the safety, the efficacy and the results after 3 years of CIPII.	**Total cholesterol:** No change (CIPII: 4.81; CSII: 4.72 mmol/L, p=NS)<br>**HDL cholesterol:** No change (CIPII: 1.14; CSII: 1.17 mmol/L, p=NS)<br>**LDL (chol.):** No change (CIPII: 3.05; CSII: 2.96 mmol/L, p=NS)<br>**LDL (trigl.):** No change (CIPII: 0.36; CSII: 0.35 mmol/L, p=NS)<br>**VLDL (chol.):** No change (CIPII: 0.29; CSII: 0.23 mmol/L, p=NS)<br>**VLDL (trigl.):** No change (CIPII: 0.43; CSII: 0.27 mmol/L, p=NS)<br>**HDL_2(chol.):** No change (CIPII: 0.26; CSII: 0.27 mmol/L, p=NS)<br>**HDL_2(trigl.):** No change (CIPII: 0.07; CSII: 0.07 mmol/L, p=NS)<br>**HDL_3(chol.):** No change (CIPII: 0.89; CSII: 0.84 mmol/L, p=NS)<br>**HDL_3(trigl.):** No change (CIPII: 0.12; CSII: 0.09 mmol/L, p=NS)<br>**Triglyceride:** No change (CIPII: 0.88; CSII: 0.81 mmol/L, p=NS)	STROBE:19/22<br>QAT:<br>Strong: Study design, data collection methods, selection bias<br>Moderate: Confounders, withdrawals and drop-outs
	Duvillard et al. 2005 (Brief report) [106]<br>Duvillard et al. 2007 [107]	N = 7<br>Age: 48<br>Diabetes duration: 17<br>Sex: 6/1<br>HbA1c: 7.34<br>C-peptide: ND<br>Reasons: ND	CSII use: ND<br>CIPII f-u: 12	Compare if replacement of SCII with IPII restores the normal physiological gradient between the portal vein and peripheral circulation, which is likely to modify lipoprotein metabolism.	**Total cholesterol:** No change (CIPII: 5.04; CSII: 5.33 mmol/L, p=0.45)<br>**HDL cholesterol:** No change (CIPII: 1.47; CSII: 1.47 mmol/L, p=0.99)<br>**LDL cholesterol**: No change (CIPII: 3.1; CSII: 3.2 mmol/L, p=0.45)<br>**Fasting plasma triglyceride:** No change (CIPII:1.28; CSII: 1.08 mmol/L, p=0.22)<br>**Apo B100-containing lipoprotein production and fractional catabolic rates**: No change (ND, p=NS)<br>**ApoA1**: No change (CIPII: 1.28; CSII: 1.34 g/L, p=0.45)<br>**HDL composition:**<br>**Esterified cholesterol:** No change (CIPII: 24.0; CSII: 20.1 %, p=0.45)<br>**Free cholesterol:** No change (CIPII: 3.3; CSII: 3.4 %, p=0.99)<br>**Triglycerides:** No change (CIPII: 2.1; CSII: 2.4 %, p=0.99)<br>**Phospholipids:** No change (CIPII: 25.2; CSII: 22.7 %, p=0.99)<br>**Proteins:** No change (CIPII: 45.5; CSII: 51.2 %, p=0.13)	Strobe: 19/22<br>QAT:<br>Moderate: Data collection methods, study design, withdrawals and drop-outs<br>Poor: Selection bias, confounders

Legends: CSII, continuous subcutaneous insulin infusion; CIPII, continuous intraperitoneal insulin infusion; ND, no data available; NS, Not significant; HDL, high density lipoprotein; LDL, low density lipoprotein; Apo, apolipoprotein; trigl., triglycerides; chol., cholesterol.

Table S2.3. (Continued)

	Source	Participant characteristics (Number, age (mean years), diabetes duration (mean years), sex (Male/Female), HbA1c (%), C-peptide, reasons to participate	Length of: CSII use, CSII follow-up, IPII follow-up (weeks)	Reported study objectives	Outcomes (mean, p-value)	Methodological quality
**Secondary outcomes: Intermediate metabolites**	**Case-control studies**				**Strengthening the Reporting of Observational Studies in Epidemiology (STROBE) and Thomas quality assessment toll (QAT):**	
	Colette et al. 1989 [114]	N = 24 (CIPII: 13 / CSII: 11)<br>Age: 30/32<br>Diabetes duration: 17/20<br>Sex: ND<br>HbA1c: 8.0/8.9<br>C-peptide: ND<br>Reasons: ND	CSII use: 40<br>CIPII use: 60	Study the effects of prolonged tight diabetic control and insulin delivery through portal route on vitamin D metabolism in IDDP.	**Plasma creatinine:** No change (CIPII: 1.08; CSII: 1.11 mg/dl, p=NS)<br>**Plasma calcium:** No change (CIPII: 9.3; CSII: 9.1 mg/dl, p=NS)<br>**Plasma magnesium:** No change (CIPII: 1.81; CSII: 1.85 mg/dL, p=NS)<br>**Plasma phosphorus:** No change (CIPII: 3.5; CSII: 3.3 mg/dL, p=NS)<br>**Plasma iPTH:** No change (CIPII: 2.6; CSII: 2.7 mU/mL, p=NS)<br>**Osteocalcin:** No change (CIPII: 5.7; CSII: 6.4 ng/mL, p=NS)<br>**Mean vitamin D intake:** No change (CIPII: 89; CSII: 99 U/day, p=NS)<br>**Vitamin D metabolites:**<br>**25 OH D:** Increases (CIPII: 22.1; CSII: 12.5 ng/mL, p<0.02)<br>**24,25-(OH)$_2$D**: Increases (CIPII: 2.3; CSII: 1.4 ng/mL, p<0.05)<br>**1,25-(OH)$_2$D:** No change (CIPII: 45; CSII: 35 pg/mL, p=NS)	STROBE: 18/22<br>QAT:<br>Strong: Data collection method, withdrawals and drop-outs<br>Moderate: Selection bias, study design, confounders
	Selam et al.1989 [82]	N = 14 (CIPII: 6 / CSII: 8)<br>Age: 32/44.3<br>Diabetes duration: 16/23.1<br>Sex: 4/2 / 5/3<br>HbA1c: 8.3/8.7<br>C-peptide: ND<br>Reasons: ND	CSII use: 52+<br>CIPII use: 26	Compare the effects of intensive SC vs. implantable pump IP insulin delivery on intermediary metabolites in DM1 patients.	**Pre-meal insulin bolus (bolus + 4 h basal rate = 0.15 U/kg): Time point 0: FFA [FF]:** Decreases (CIPII: 0.20; CSII: 0.47 mmol/L, p<0.05)<br>**Postprandial FFA [FF]:** Decreases (at +30min: CIPII: 0.2; CSII: 0.45 mmol/L, p<0.05); decreases (+60 min; CIPII: 0.2; CSII: 0.47 nmol/L, p=0.05)<br>**Time point 0: lactate [FF]:** No change (CIPII: 0.5; CSII: 0.45 mmol/L, p=NS)<br>**Postprandial lactate [FF]:** Increases (at +30 minutes: CIPII: 0.7; CSII: 0.4 mmol/L, p=NS. At +60 min.: CIPII: 1.0; CSII: 0.5 mmol/L, p<0.05)<br>**Alanine [FF]:** No change (p=NS)<br>**3 OH butyrate [FF]:** No change (p=NS)	STROBE: 14/22<br>QAT:<br>Strong: Data collection methods<br>Moderate: Study design, confounders<br>Weak: Confounders<br>Unclear: Selection bias
	Van Dijk et al. 2016 [93]<br>Van Dijk et al. 2020 [117]	N = 181 (CIPII: 39 / CSII: 74<br>Age: 49.6/47.9<br>Diabetes duration: 28.5/24.7<br>Sex: 14/25 30/44<br>HbA1c: 66.9/63.4<br>C-peptide: neg<br>Reasons: Poor glucose control*	CSII use: 208<br>CSII follow-up: 26<br>CIPII use: 208<br>CIPII follow-up: 26	To test the hypothesis that among persons with T1DM treated with IP insulin therapy there is a decreased calcification propensity (expressed as a higher T50) as compared with treatment with SC insulin therapy.	**Calcium:** no change (CIPII: 2.3; CSII: 2.3 mmol/L, p=ND)<br>**T_{50} within groups**: no change (CIPII baseline: 372; CIPII end: 362 minutes, difference within group: (median [with interquartile range (IQR)]) -10[-29,9]<br>no change (CSII baseline: 360; CSII end: 359 minutes, difference within group: (median [with interquartile range (IQR)]) -0.2[-19,9]<br>**T_{50} after follow-up**: no change after (CIPII: 362; CSII: 359 minutes, difference CIPII vs. CSII: (median [with interquartile range (IQR)]) -8 [-22,7]	STROBE: 21/22<br>QAT:<br>Strong: Data collection method, study design<br>Moderate: Confounders<br>Unclear: Selection bias

Legends: CSII, Continuous subcutaneous insulin infusion; CIPII, Continuous intraperitoneal insulin infusion; ND, No data available; Neg, negative; NS, Not significant; FFA, Free fatty acids; iPTH, Immunoreactive parathyroid hormone; 25 OH D, Calcifediol; 24,25-(OH)$_2$D, (inactive) hydroxycalcidiol; 1,25-(OH)$_2$D, active form of vitamin D_3; 3 OH butyrate, beta-hydroxybutyrate (by-product of ketosis); [FF], data extracted from figure; *, HbA1 c⩾ 58 mmol/mol (7.5 %) or at least five incidents of hypoglycaemia (defined as glucose < 4.0 mmol/L).

Table S2.4. Intervention studies, Participant characteristics, description, outcomes: Counterregulatory hormones

	Source	Participant characteristics (Number, age (mean years), diabetes duration (mean years), sex (Male/Female), HbA1c (%), C-peptide, reasons to participate	Length of: CSII use, CSII follow-up, IPII follow-up (weeks)	Reported study objectives	Outcomes (mean, p-value)	Methodological quality
Secondary outcomes: Counterregulatory hormones	**Non-randomised crossover studies**				**Strengthening the Reporting of Observational Studies in Epidemiology (STROBE) and Thomas quality assessment toll (QAT):**	
	Hanaire-Broutin et al. 1996 [101]	N = 18<br>Age: 43.0<br>Diabetes duration: 20.0<br>Sex: 11/7<br>HbA1c: 7.6<br>C-peptide: Neg<br>Reasons: Volunteers	CSII use: 128<br>CIPII f-u: 52	To evaluate the impact of intraperitoneal insulin therapy, which results in preferential insulin absorption by the portal system, on the hepatic growth hormone-resistant state of DM1.	**Fasting growth hormone:** No change (CIPII: M3: 3.46; M12: 1.47; CSII: 2.23 ng/mL)<br>**GHBP activity** [DT]: Increases (CIPII: M3: 14.5; M12: 15.5; CSII: 10.2 %, p<0.0001)	STROBE: 16/22<br>QAT:<br>Strong: Study design, data collection methods, withdrawals and drop-outs<br>Moderate: Selection bias, confounders
	Oskarsson et al. 1999 [90]	N = 7<br>Age: 42<br>Diabetes duration: 15<br>Sex: 5/2<br>HbA1c: 8.5<br>C-peptide: < 0.2nM<br>Reasons: Unsatisfactory on CSII	CSII use: 26+<br>CIPII f-u: 61	To assess the clinical relevance of the blood glucose, hypoglycaemia, glucagon secretion during exercise by comparing glycaemic and hormonal responses to a 40-min bicycle exercise test at 60% of $VO_{2\,max}$ during CSII and CIPII in type 1 diabetic patients.	**Change in hormone levels from pre- to post-exercises; and change between CIPII and CSII:**<br>**Glucagon:** Increases (CIPII: 15.1, p=0.01; CSII: 7.4 pg/mL, p=0.08)**;** no change (CIPII vs CSII: p=0.07)<br>**Epinephrine:** Increases in both groups (CIPII: 0.81, p=0.03; CSII: 0.43 nmol/L, p=0.009); no change (CIPII vs CSII: p=0.49)<br>**Norepinephrine**: Increases in both groups (CIPII: 3.75, p=0.006; CSII: 4.02 nmol/L, p=0.006); no change (CIPII vs CSII: p=0.09)<br>**Growth hormone:** Increases in both groups (CIPII: 9.4, p=0.03; CSII: 11.9 mg/mL, p=0.01); no change (CIPII vs CSII: p=0.34)<br>**Cortisol**: Increases in both groups (CIPII: 135.1, p=0.02; CSII: 92.9 nmol/L, p=0.03); no change (CIPII vs CSII: p=0.47)<br>**C-peptide:** No change (CIPII: -0.02, p=0.19; CSII: -0.01 nmol/L, p=0.59); no change (CIPII vs CSII: p=0.91)	STROBE:16/22<br>QAT:<br>Strong: Confounders, data collection methods, withdrawals and drop-outs<br>Moderate: Selection bias, study design
	Oskarsson et al. 2000 [89]	N = 7<br>Age: 42<br>Diabetes duration: 17<br>Sex: 5/2<br>HbA1c: 8.6<br>C-peptide: Neg<br>Reasons: Unsatisfactory on CSII	CSII use: 52+<br>CIPII f-u: 69	To expose the patients to an identical hyperinsulinemic challenge with special emphasis on the glucagon response in the same patients during continuous treatment with CSII and CIPII.	**Change in plasma hormone levels from basal level to peak level in time of hyperinsulinemia; and change between CIPII and CSII:**<br>**Glucagon**: Increases (CIPII: 17.0, p=0.003; CSII: 7.5 pg/mL, p=0.06)**;** increases (CIPII vs CSII: p=0.048)<br>**Epinephrine:** Increases in both groups (CIPII: 2.05, p=0.004; CSII: 2.92 nmol/L, p=0.04); no change (CIPII vs CSII: p=0.50)<br>**Norepinephrine**: Increases (CIPII: 0.91, p=0.003; CSII: 0.74 nmol/L, p=0.11); no change (CIPII vs CSII: p=0.68)<br>**Growth hormone**: Increases in both groups (CIPII: 13.4, p=0.02; CSII: 19.3 mg/mL, p=0.03); no change (CIPII vs CSII: p=0.34)<br>**Cortisol:** Increases in both groups (CIPII: 286, p=0.0003; CSII: 277 nmol/L, p=0.0003); no change (CIPII vs CSII: p=0.77)<br>**C-peptide:** No change (CIPII: 0.02, p=0.30; CSII: 0.05 nmol/L, p=0.74); no change (CIPII vs CSII: p=0.44)	STROBE: 16/22<br>QAT:<br>Strong: Confounders, data collection methods, withdrawals and drop-outs<br>Moderate: Selection bias, study design

Legends: CSII, Continuous subcutaneous insulin infusion; CIPII, Continuous intraperitoneal insulin infusion; ND, No data available; NS, Not significant; FFA GHBP, Growth hormone binding proteins; [DT], data calculated from table.

Table S2.4. (Continued)

	Source	Participant characteristics (Number, age (mean years), diabetes duration (mean years), sex (Male/Female), HbA1c (%), C-peptide, reasons to participate	Length of: CSII use, CSII follow-up, IPII follow-up (weeks)	Reported study objectives	Outcomes (mean, p-value)	Methodological quality
**Secondary outcomes: Counterregulatory hormones**	**Non-randomised follow-up studies**			**Strengthening the Reporting of Observational Studies in Epidemiology (STROBE) and Thomas quality assessment toll (QAT):**		
	Van Dijk et al. 2016 [93]	N = 113 (CIPII: 39/CSII: 74)<br>Age: 50/48<br>Diabetes duration: 29/27<br>Sex: 14/25 / 30/44<br>HbA1c: 8.3/7.9<br>C-peptide: ND<br>Reasons: Pmc	CSII/MDI use: 208+<br>CIPII use: 208+<br>CSII f-u: 27<br>CIPII f-u: 27	To compare the effects of CIPII to SC insulin therapy, on the GH-IGF-1 axis in a large prospective, observational matched case-control study in T1DM patients.	**Growth hormone:** Decreases (CIPII: 0.63; CSII: 1.39 µg/L, p=0.039)	STROBE: 16/22<br>QAT:<br>Strong: Selection bias, study design, data collection method<br>Moderate: Study design, withdrawals and drop-outs
	**Case-control studies**					**STROBE and QAT:**
	Selam et al. 1989 [82]	N = 14 (CIPII: 6 /CSII: 8)<br>Age: 32/44.3<br>Diabetes duration: 16/23.1<br>Sex: 4/2 / 5/3<br>HbA1c: 8.3/8.7<br>C-peptide: ND<br>Reasons: ND	CSII use: 52+<br>CIPII use: 26	Compare the effects of intensive SC vs. implantable pump IP insulin delivery on intermediary metabolites in DM1 patients.	**Fasting glucagon [FF]:** No change (CIPII: 25; CSII: 25 pg/mL, p=NS)<br>**Postprandial glucagon [FF]** (+30 minutes): No change (CIPII: 30; CSII: 20 pg/mL, p=NS)	STROBE: 14/22<br>QAT:<br>Strong: Data collection methods<br>Moderate: Study design, confounders<br>Weak: Confounders<br>Unclear: Selection bias, blinding<br>Not applicable: Withdrawals and drop-outs

Legends: CSII, Continuous subcutaneous insulin infusion; CIPII, Continuous intraperitoneal insulin infusion; Pmc, Poor metabolic control; c.a., Conference abstract; ND, No data available; NS, Not significant; NP, Not possible to evaluate; [FF], data extracted from figure.

Table S2.5. Intervention studies, Participant characteristics, description, outcomes: Other outcomes

	Source	Participant characteristics (Number, age (mean years), diabetes duration (mean years), sex (Male/Female), HbA1c (%), C-peptide, reasons to participate	Length of: CSII use, CSII follow-up, IPII follow-up (weeks)	Reported study objectives	Outcomes (mean, p-value)	Methodological quality
Other outcomes	**Non-randomised crossover studies**				**Strengthening the Reporting of Observational Studies in Epidemiology (STROBE) and Thomas quality assessment toll (QAT):**	
	Wredling, Adamson et al. 1991 (Technical report) [91]	N = 6<br>Age: 41.3<br>Diabetes duration: 23.2<br>Sex: 4/2<br>HbA1c: 8.7<br>C-peptide: Neg<br>Reasons: Pmc	CSII use: 52+<br>CSII f-u: 8 (n=3)<br>CIPII f-u: median 18 (15 – 24 months)	To determine the efficacy of a new percutaneous device.	**Anti-insulin antibodies**: No change (CIPII: 34.8; CSII: 21.7 %, p=NS)	STROBE:15/22<br>QAT:<br>Moderate: Selection bias, study design, data collection method<br>Weak: Withdrawals and drop-outs<br>Unclear: Confounders
	Lassmann-Vague et al. 1994 (short communication) [104]	N = 11<br>Age: 34.4<br>Diabetes duration: 22.4<br>Sex: 5/6<br>HbA1c: 6.9<br>C-peptide: Neg<br>Reasons: ND	CSII use: 26+<br>CIPII f-u: 12	ND	**SHBG levels in men**: Decreases (CIPII: M1: 31; M3: 33; CSII: 39 nM/L, p<0.05)<br>**SHBG levels in women**: Decreases (CIPII: M1: 67; M3: 63; CSII: 80 nM/L, p<0.01)	NP
	Raccah et al. 1994 (letter) [109]	N = 11<br>Age: 34.4<br>Diabetes duration: 22.3<br>Sex: 6/5<br>HbA1c: 6.9<br>C-peptide: ND<br>Reasons: ND	CSII use: 12<br>CIPII f-u: 40	ND	**Plasminogen activator inhibitor (PAI) 1** levels: No change (CIPII: M3: 4; M10: 6.6; CSII: 5.1 U/mL, p=NS)	NP
	Hanaire-Broutin et al. 1996 [101]	N = 18<br>Age: 43.0<br>Diabetes duration: 20.0<br>Sex: 11/7<br>HbA1c: 7.6<br>C-peptide: Neg<br>Reasons: Volunteers	CSII use: 128<br>CIPII f-u: 52	To evaluate the impact of intraperitoneal insulin therapy, which results in preferential insulin absorption by the portal system, on the hepatic growth hormone-resistant state of DM1.	**Plasma IGF I** [DT]: Increases (CIPII: M3: 114.0; M12: 146.9; CSII: 89.4 ng/mL, p<0.002)<br>**IGFBP-3** [DT]: Increases (CIPII: M3: 2275; M12: 3534; CSII: 1974 ng/mL, p<0.0001)	STROBE: 16/22<br>QAT:<br>Strong: Study design, data collection methods, withdrawals and drop-outs<br>Moderate: Selection bias, confounders
	Lassmann-Vague et al. 1995 [79]<br>Lassmann-Vague et al. 1998 (letter) [80]	N = 15<br>Age: 36<br>Diabetes duration: 20.9<br>Sex: 8/9<br>HbA1c: 7.1<br>C-peptide: Neg<br>Reasons: ND	CSII use: ND<br>CSII f-u: 4<br>CIPII f-u: 104	To assess immunogenicity of intraperitoneal insulin infusion via implanted pumps by two methods. To evaluate the possible influence of an increased antibody level on metabolic and clinical parameters.	**Anti-insulin antibodies**[Ꜫ] (measured by using RIA) [DT]: Increases (CIPII: M3: 39.9, p<0.01; M12: 42.5, p<0.01; M24: 48, p=0.964; CSII: 23.7 %)	STROBE: 12/22<br>QAT:<br>Moderate: Selection bias, study design, data collection method<br>Weak: Withdrawals and dropouts<br>Unclear: Confounders

Legends: CSII, Continuous subcutaneous insulin infusion; CIPII, Continuous intraperitoneal insulin infusion; Pmc, Poor metabolic control; ND, No data available; NS, Not significant; NP, Not possible to evaluate; SHBG, Sex hormone binding globulin; IGF 1, Insulin-like growth factor – 1; BP, Binding proteins; [Ꜫ], 100 % is optical density between 1.5 and 2 U of AI IgG in solution; RIA, radioimmunoassay; [DT], data calculated from table.

Table S2.5. (Continued)

	Source	Participant characteristics (Number, age (mean years), diabetes duration (mean years), sex (Male/Female), HbA1c (%), C-peptide, reasons to participate	Length of: CSII use, CSII follow-up, IPII follow-up (weeks)	Reported study objectives	Outcomes (mean, p-value)	Methodological quality
Other outcomes	**Non-randomised crossover studies**				**Strengthening the Reporting of Observational Studies in Epidemiology (STROBE) and Thomas quality assessment toll (QAT):**	
	Duvillard et al. 2005 (Brief report) [106]<br>Duvillard et al. 2007 [107]	N = 7<br>Age: 48<br>Diabetes duration: 17<br>Sex: 6/1<br>HbA1c: 7.34<br>C-peptide: ND<br>Reasons: ND	CSII use: ND<br>CIPII f-u: 12	Compare if replacement of SCII with IPII restores the normal physiological gradient between the portal vein and peripheral circulation, which is likely to modify lipoprotein metabolism.	**Fructosamine:** No change (CIPII: 352; CSII: 348 µmol/L, p=0.69)	Strobe: 19/22<br>QAT:<br>Moderate: Data collection methods, study design, withdrawals and drop-outs<br>Poor: Selection bias, confounders
	Dassau et al. 2017 [78]	N = 10<br>Age: 49<br>Diabetes duration: 29<br>Sex: 7/3<br>HbA1c: 7.7<br>C-peptide: ND<br>Reasons: Poor metabolic control	CSII use: 443<br>CSII f-u: 24h<br>CIPII f-u: 4 to 20<br>Washout: 4 to 20	To compare closed-loop zone MPC using the DiaPort IP insulin delivery system with the traditional SC insulin delivery method during a 24-hour in-clinic protocol.	**Anti-insulin antibodies:** No change (ND)	STROBE: 20/22<br>QAT:<br>Strong: Data collection methods, withdrawals and drop-outs, study design<br>Moderate: Selection bias, confounders
	**Non-randomised follow-up studies**					**STROBE and QAT:**
	Jeandidier et al. 2002 [115]	N = 24 (CIPII: 13/CSII: 11)<br>Age: 36.8/43.1<br>Diabetes duration: 19.2/24.4<br>Sex: 6/7 / 6/5<br>HbA1c: ND<br>C-peptide: Neg<br>Reasons: ND	CSII/MDI use: ND<br>CSII f-u: 26<br>CIPII f-u: 26	To assess the antigenicity of the insulin Hoechst 21PH using CSII and to compare the antigenicity of this insulin when administered IP or SC.	**Anti-insulin antibodies:** (measured by using RIA): Increases (CIPII: M6: 41.8; CSII: M6: 24.9 %, p=0.009)<br>**ELISA:** No change (CIPII: M6: 10.1; CSII: 4.4 %, p=0.07)	STROBE: 16/22<br>QAT:<br>Strong: Data collection methods, withdrawals and drop-outs<br>Moderate: Selection bias, study design, confounders
	Van Dijk et al. 2016 [93]	N = 113 (CIPII: 39/CSII: 74)<br>Age: 50/48<br>Diabetes duration: 29/27<br>Sex: 14/25 / 30/44<br>HbA1c: 8.3/7.9<br>C-peptide: ND<br>Reasons: Pmc	CSII/MDI use: 208+<br>CIPII use: 208+<br>CSII f-u: 27<br>CIPII f-u: 27	To compare the effects of CIPII to SC insulin therapy, on the GH-IGF-1 axis in a large prospective, observational matched case-control study in T1DM patients.	**IGF-1:** Increases (CIPII: 123; CSII: 107 µg/L, P=NS)<br>**IGFBP-1:** Decreases (CIPII: 40.2; CSII: 85.4 µg/L, p=0.004)<br>**IGFBP-3:** Increases (CIPII: 3.75; CSII: 3.22 mg/L, p=0.015)	STROBE: 16/22<br>QAT:<br>Strong: Selection bias, study design, data collection method<br>Moderate: Study design, withdrawals and drop-outs

Legends: CSII, Continuous subcutaneous insulin infusion; CIPII, Continuous intraperitoneal insulin infusion; ND, No data available; ELISA, enzyme-linked immunosorbent assay; RIA, radioimmunoassay.

Table S2.5. (Continued)

	Source	Participant characteristics (Number, age (mean years), diabetes duration (mean years), sex (Male/Female), HbA1c (%), C-peptide, reasons to participate	Length of: CSII use, CSII follow-up, IPII follow-up (weeks)	Reported study objectives	Outcomes (mean, p-value)	Methodological quality
**Other outcomes**	**Retrospective crossover studies**					**STROBE and QAT:**
	Jeandidier et al. 1992 (Preliminary results) [86]	N = 8<br>Age: 33.5<br>Diabetes duration: 14.5<br>Sex: ND<br>HbA1c: 6.64<br>C-peptide: Neg<br>Reasons: ND	CSII use: 1<br>CIPII use: 12	To assess the potential benefits of CIPII vs SCII.	**Anti-insulin antibodies:** Increases (CIPII: 11.0; CSII: 3.6 %, p<0.05)	STROBE: 12/22<br>QAT:<br>Weak: Study design<br>Unclear: Selection bias, confounders, data collection methods
	**Case-control studies**					**STROBE and QAT:**
	Hedman et al. 2009 (c.a.) [111]<br>Arnqvist et al. 2010 (c.a.) [110]<br>Hedman et al. 2014 [112]	N = 30 (CIPII: 10 /CSII: 20)<br>Age: 53.1/52.8<br>Diabetes duration: 124.2/30.8<br>Sex: 5/5 / 10/10<br>HbA1c: 8.6/7.9<br>C-peptide: ND<br>Reasons: Pmc	CSII use: 26+<br>CIPII use: 26+	Investigate in cross-sectional study if the different modes of insulin administration, CIPII or CSII were associated with a change in the circulating IGF system.	**Fasting levels of bioactive IGF-I:** Increases (CIPII: 1.83; CSII: 1.16 μg/L, p=0.024).<br>**Total IGF-I:** Increases (CIPII: 120; CSII: 81 μg/L, p=0.007)<br>**IGF-II:** Increases (CIPII: 1050; CSII: 879 μg/L, p=0.015)<br>**IGFBP-1**: Decreases (p=0.013)<br>**IGFBP-2:** No change (p=NS)	STROBE: 21/22<br>QAT:<br>Strong: Selection bias, confounders, data collection method, withdrawals and drop-outs<br>Moderate: Study design

Legends: CSII, Continuous subcutaneous insulin infusion; CIPII, Continuous intraperitoneal insulin infusion; Pmc, Poor metabolic control; ND, No data available; NS, Not significant; NP, Not possible to evaluate; IGF 1, Insulin-like growth factor – 1; BP, Binding proteins.

Table S2.6. Technical and physiological complications with intraperitoneal insulin pump and its attached system

**Study ID**	**Study design**	**Nr. of partici-pants**	**Min. CIPII-period (months)**	**Min. CIPII-period (patient-years)™**	**Complications (events/study) during CIPII-period**											
					Local infection/ inflam-mation	Severe abdo-minal pain	Severe insulin under-delivery (catheter obstruction / encapsulation)	Eryt-hema	Pump change/ reimplan-tation	Catheter change	Necrosis in abdominal skin «pocket”	Exhaustion of batteries of pump	Peritoneal abscess	Loss of catheter	Removal of implanted system because of compli-cations	Insulin pumps technical problems
Liebl et al. 2009 [5]	RFUs[a]	CIPII: 30	12	30	20	9	6	-	-	-	-	-	-	-	8	-
Wredling, Adamson et al. 1991 [91]	NRCs	6	15	9.4ª	1	3	4	6	-	-	-	-	-	-	5	-
Pitt et al. 1992 [6]	NRCs	10	34	28.3	-	-	6	?	12	1	-	-	-	-	1	2
Renard et al. 1993 [81]	NRCs	8	12[⊕]	-EP: 12ª<br>-CSII: 9ª	-	-	-EP: 13<br>-CSII: 0	-	0	-	-	-	-	-	0	26
Schnell et al. 1994 [105]	NRCs	5	12	5	-	-	1	-	1	-	-	-	1	1	-	-
Hanaire-Broutin et al. 1996 [101]	NRCs	18	12	18	-	-	-	-	-	-	-	-	-	-	-	0
Pacifico et al. 1997 [98]	NRCs	8	12	8	-	-	6	-	-	-	1	2	-	-	9	1
Liebl et al. 2013/2014 [94-97]	NRCs	12	24	24	5	-	-	-	1	8	-	-	-	-	-	-
Dassau et al. 2017 [78]	NRCs	10	1	0.8	-	-	0	-	-	-	-	-	-	-	-	0
Jeandidier et al. 1992 [86]	Retro. Cs	8	10	6.7	-	-	8	-	-	-	-	-	-	-	8	-
**TOTAL**		**115**	**144**	**130.2[+]**	**26**	**12**	**44**	**6**	**14**	**9**	**1**	**2**	**1**	**1**	**31**	**29**

Legends: CIPII, Continuous intraperitoneal insulin infusion; RCs, Randomised crossover study; RFUs, Randomised follow-up study; NRCs, Non-randomised crossover study; Retro.Cs, Retrospective crossover study; C-Cs, Case-control study; NRFUs, Non-randomised follow-up study; (–), no data available; ª, authors provided data; [a], dropouts in this study (at the end of the periods N = 36 (CIPII: 15 /CSII: 21); [⊕], included patients with previous use of external CIPII (-EP) and with previous CSII (-CSII); [+], Renard et al. study is not included; ™, multiplication of the number of patients and min. CIPII-period

Table S2.7. Methodological aspects of the included studies.

Study ID	Study design	Min. CSII period (month)	Min. CIPII period (month)	CSII-period insulin	CIPII-period insulin	CIPII implantation system	Insulin pump (CSII/CIPII)	CIPII catheter position (quadrant)	SMBG tests (times/day)	SMBG parameter	Nr. of laboratory visits during the study (CSII/CIPII)
Micossi et al. 1986 [84]	NRCs	12	1 ½	-	-	Siemens	Microjet syringe/Promedos E1[E]	4 cm below umbilicus	6: Fasting, before and 2-h after lunch and dinner, at bedtime	-	1/1
Beylot et al. 1987 [103]	NRCs	2	2	Porcine	-	Siemens AG	Betatron IICPJ 9200/Promedos	Umbilical area	3-6	Mean of all BG data from second months of treatment	1/1
Colette et al. 1989 [114]	C-Cs	7	10	Actrapid (regular) or CS21 Hoechst U40	CS21 Hoechst U40 (regular)	-	Microjet Infuser or Promedos/ Promedos[P]	Through umbilicus	-	-	1/1
Selam et al. 1989 [82]	C-Cs	12	6	-	Hoechst U400 (surfactant stabilized)	PIMS (telemetry using a battery-operated programmer)	ND/MiniMed[I]	Lower portion of the IP cavity	-	-	1/1
Walter et al. 1989 [99]	C-Cs	6	3	Semisynthetic human insulin U100	Semisynthetic human insulin U40	-	Betatron II; AS8MP/Promedos E1	-	-	-	1/1
Wredling, Adamson et al. 1991 [91]	NRCs	12	15	-	Velosulin Human (2 mo, n=2), afterwards H-Tronin	Percuseal	-/-[E]	Upper right (n=1), upper left (n=2), lower left (n=3)	-	-	1/ every 4 weeks
Wredling, Liu et al. 1991 [92]	NRCs	24	6.9	Velosulin Human U100	H-Tronin U100	Percuseal	MiniMed 504-S /MiniMed 504-S[E]	-	4: before each meal + before evening snack	-	2/2
Georgopoulos et al. 1992 [83]	NRCs	ND	12	-	-	PIMS	-/-	-	4-6	Mean blood glucose over 4 weeks before end of the period	1/1
Jeandidier et al. 1992 [86]	Retro. Cs	ND	10	-	Hoechst 21 PH U100	Telemetry using a battery-operated programmer.	-/Infusaid 1000[I]	-	-	-	1/1

Pitt et al. 1992 [6]	NRCs	3	34	-	Hoechst U400	PIMS	-[E]/-[I]	Left from umbilicus above or below the waistline	2-4	Mean of all BG values for the 2 mo before and each 2 mo after implantation	2/9
Giacca et al. 1993 [100]	RCs	96 hours	3	HOE21gh U100 (human)	HOE21gh U100 (human)	-	Microjet MC-20/Promedos ID 1[I]	-	-	-	1/1
Renard et al. 1993 [81]	NRCs	2.4	12	Porcine (Velosulin) U100	Hoechst 21 PH U400 (for MiniMed pump) U100 (for Insufaid pump)	-	Portable pump/ MiniMed 2001[I] (n=6) or Insufaid 1000[I] (n=2)	-	-	-	1/4 (3,6,9,12 mo)
Georgopoulos et al. 1994 [102]	NRCs	ND	6	-	-	-	-/-	-	4-6	Mean blood glucose over 4 weeks before end of the period	1/1
Lassmann-Vague et al. 1994 [104]	NRCs	6	3	-	Hoechst 21 PH U100 (for Infusaid) or U400 (for MIP)	-	ND/Infusaid 1000[I] or MiniMed MIP 2001[I]	-	-	Mean of monthly blood glucose	2/2 (-1,0/1,3 mo)
Raccah et al. 1994 [109]	NRCs	3	10	-	-	-	ND/Infusaid 1000[I] (n=6) or MIP 2001[I] (MiniMed) (n=5)	-	4-5	Mean of monthly blood glucose	1/3 (1,3,10 mo)
Schnell et al. 1994 [105]	NRCs	36	12	-	-	Percuseal	-	Left of right above navel	-	-	1/2 (3,12 mo)
Lassmann-Vague et al.1995/1998 [79, 80]	NRCs	1	24	Actrapid U100 (n=3), Velosulin U100 (n=10), Ultratardum U40 (n=2)	Hoechst 21 PH U100 (for Infusaid) or U400 (for MIP)	-	ND/ Infusaid 1000[I] (n=4) or MIP 2001[I] (n=11)	-	4	-	1/3 (3,12,24 mo)
Guerci et al. 1996 [108]	NRCs	14.2	4	-	Hoechst 21 PH U400	Battery-operated telemetry systems	ND[E]/MiniMed 2001[I]	Lower left	-	Mean of monthly blood glucose	1/2 (2,4 mo)
Hanaire-Broutin et al. 1996 [101]	NRCs	3	12	-	-	-	ND[E]/MIP 2001 (MiniMed)[I]	-	>4	-	1/2 (3,12 mo)
Lassmann-Vague et al. 1996 [87]	NRCs	ND	2	Actrapid Novo (n=6) or Velosulin	Hoechst 21 PH U100 (n=4) U400 (n=7)	-	ND/ND[I]	-	-	-	1/1

				Nordisk (n=5)							
Pacifico et al. 1997 [98]	NRCs	3	12	-	Hoechst 21 PH U400	-	ND/MIP 2001[I] (MiniMed)	Lower left	-	-	1/2 (6,12 mo)
Oskarsson et al. 1999 [90]	NRCs	6	11	-	-	-	MiniMed 506/ MiniMed 2001[I]	-	-	-	1/1
Oskarsson et al. 2000 [89]	NRCs	12	11	-	-	-	MiniMed 506/ MiniMed 2001[I]	-	5: morning, before lunch and dinner, 2 h after dinner, before bed	Mean of monthly blood glucose	1/1
Catargi et al. 2002 [88]	Retro. Cs	1.5	3*	Lispro U100	Hoechst 21 PH U400	Telemetry using a battery-operated programmer.	MiniMed 506 or 507/MIP 2001[I] or 2007[I] (MiniMed)	Lower left	>4	Mean of all BG values for the periods (45 days/ last 45 days)	1/1
Jeandidier et al. 2002 [115]	NRFUs	6	6	Regular or Lente or Humalog	Insuman Infusat U100	-	H-Tron/ MIP 2001[I] (MiniMed)	-	-	-	3/3 (0,3,6 mo)
Duvilard et al. 2005/2007 [106, 107]	NRCs	ND	3	-	-	-	MiniMed 506 or 507/Minimed 2007C[I] or 2007A[I]	-	-	-	1/1
Liebl et al. 2009 [5]	RFUs	6	12	Lispro U100	Insuman Infusat U100 or H-Tronin U100	Diaport	H-TRONplus/ H-TRONplus	Lower left or right	4: prior each meal+ before bedtime	-	1/1
Hedman et al. 2009/2014 [111, 112] Arnqvist et al. 2010 [110]	C-Cs	6	6	Aspart U100(Novo rapid) or lispro U100 (Humalog)	Semisynthetic human insulin of porcine origin (Sanofi) U400	-	ND/MIP 2007C[I] (Medtronic/Minimed)	-	-	-	1/1
Liebl et al. 2013/2014 [94-97]	NRCs	-	24	-	-	DiaPort	ND/Accu-Chek[E]	-	-	-	1/4 (3,6,12,24 mo)
van Dijk et al. 2016 [93] van Dijk et al 2020 [117]	NRFUs	48	48	Fast acting	Human U400 (of E. coli origin)	-	ND/MIP 2007D[I]	-	-	-	2/2 (0,6 mo)
Dassau et al. 2017 [78]	NRCs	102	1	Fast acting	Insuman Infusat U100 (regular)	DiaPort	Accu-Check Spirit Combo[Φ,E]/ Accu-Check Spirit Combo[Φ,E]	-	CGM (every 5 min)	-	1/1

Legends: CSII, Continuous subcutaneous insulin infusion; CIPII, Continuous intraperitoneal insulin infusion; RCs, Randomised crossover study; RFUs, Randomised follow-up study; NRCs, Non-randomised crossover study; Retro.Cs, Retrospective crossover study; C-Cs: Case-control study; NRFUs, Non-randomised follow-up study; ND, No data available; Asterix (*), three patients first were treated with CIPII, and then with CSII; ϕ, pump provided only for 24-hour glucose profile; PIMS, The programmable implantable medication system; MIP, MiniMed Implantable Pump; [E], external insulin pump; [I], implantable insulin pump; [P], peristaltic pump; (–), no data available; mo: months. Note: Studies are sorted by year of publication.

Table S2.8. Glycaemic control during the CIPII-period: Hypoglycaemia, normoglycaemia and hyperglycaemia events and/or time spent in

Study ID	Study design	Nr. of partici-pants	Minimal CIPII period (month)	Hypo-glycaemic coma	Severe hypo-glycaemic events/ patient-year (requiring assistance)	Hypo-glycaemic events/ patient year (BG < 3.0 mmol/L)	Time spent in hypo-glycaemia (BG < 2.8 mmol/L), % ± SD	Time spent in hypo-glycaemia (BG < 3.9 mmol/L), % ± SD	Time spent in normo-glycaemia (3.9 – 10.0 mmol/l)[AP], %	Time spent in normo-glycaemia (4.4 – 7.8 mmol/L), %	Time spent in hyper-glycaemia (BG > 10 mmol/L), % ± SD	Time spent in hyper-glycaemia (BG > 14 mmol/L), % ± SD
**Micossi et al. 1986 [84]**	NRCs	6	1 ½	-	-	-	1.65±0.51	4.51±2.42	-	-	31.84±19.66	8.9±8.69
**Pitt et al. 1992 [6]**	NRCs	10	84	0	0.43	>1 /patient	-	8.8-6.0 (MIP)	-	-	M2-16:15±5 M18:20±5 (MIP)	-
**Renard et al. 1993 [81]**	NRCs	8	12	0	0	-	M3: 10.0±7.2 M6: 7.6±7.7 M9: 6.1±5.5 M12: 6.1±6.1	-	-	-	M3: 11.9±6.8 M6: 14.3±8.5 M9: 13.6±6.4 M12: 13.1±4.5	-
**Pacifico et al. 1997 [98]**	NRCs	8	12									
**Oskarsson et al. 1999 [90]**	NRCs	7	11	-	-	8.4	-	-	-	-	-	-
**Oskarsson et al. 2000 [89]**	NRCs	7	11	-	-	8.4	-	-	-	-	-	-
**Liebl et al. 2009 [5]**	RFUs	(CIPII: 30 /CSII: 30)	12	-	Total: 0.35: M1-3: 0.72; M4-12: 0.15	Total:118.2: M1-3: 138.1; M4-12: 108.9	-	-	-	-	-	-
**Liebl et al. 2013/2014 [94-97]**	NRCs	12 (n=10)*	24	-	1.5	-	-	-	M6: 58	-	M6: 38	-
**Dassau et al. 2017 [78]**	NRCs	10	1	-	-	-	-	2.5±2.9	65.7±9.2	39.8±7.6	32.4±8.9	5.9±5.6

Legends: RCs, Randomised crossover study; RFUs, Randomised follow-up study; NRCs, Non-randomised crossover study; Retro.Cs, Retrospective crossover study; C-Cs, Case-control study; NRFUs, Non-randomised follow-up study; ND, No data available; [AP], suggested BG range for artificial pancreas systems; (–), no data available; Asterix (*), dropouts in the study; M, month.

Table S2.9. Data modification for STATA: HbA1c.

Study ID	Data in forest plot, HbA1c (%)						Original data								
	CIPII			CSII			CIPII				CSII				Unit
	Mean	SD	Total	Mean	SD	Total	Mean	SD	SEM	Total	Mean	SD	SEM	Total	
**Georgopoulos et al. 1992 [83]**	7.7	1.2	7	9.8	1.4	7	7.7	1.2	-	7	9.8	1.4	-	7	%, SD
**Liebl et al. 2013/2014 [94-97]**	7.2	0.5	10	8.8	1.2	10	7.2	0.54	-	10	8.8	1.15	-	10	%, SD
**Oskarsson et al. 1999 [90]**	7.1	0.5	7	8.5	0.8	7	7.1	-	0.2	7	8.5	-	0.3	7	%, SEM
**Oskarsson et al. 2000 [89]**	7.2	0.5	7	8.6	1.1	7	7.2	-	0.2	7	8.6	-	0.4	7	%, SEM
**Schnell et al. 1994 [105]**	8.5	0.5	5	9.8	0.7	5	8.5	0.5[κ]	-	5	9.8	0.7[κ]	-	5	%, SD
**Wredling, Adamson et al. 1991 [91]**	7.6	0.4	6	8.7	0.6	6	7.6*	-	-	6	8.7*	-	-	6	%, (min-max)
**Pitt et al. 1992 (data extracted from figure by IDF) [6]**	8	1.8	10	9.1	2.2	10	-	-	-	10	-	-	-	10	%, SEM
**Colette et al. 1989 [114]**	8	1.4	13	8.9	2	11	8	-	0.4	13	8.9	-	0.6	11	%, SEM
**Georgopoulos et al. 1994 [102]**	8.7	1.2	8	9.4	1.5	8	8.7	1.2	-	8	9.4	1.5	-	8	%, SD
**Raccah et al. 1994 [109]**	6.3	1	11	6.9	1	11	6.3	-	0.3	11	6.9	-	0.3	11	%, SEM
**Catargi et al. 2002 [88]**	7.3	0.8	14	7.8	0.9	14	7.3	0.8	-	14	7.8	0.9	-	14	%, SD
**Selam et al. 1989 (SD calculated in SPSS by IDF) [82]**	8.2	1.4	6	8.6	1.3	8	-	-	-	6	-	-	-	8	%
**Lassmann-Vague et al. 1994 [104]**	6.8	0.7	11	6.9	1	11	6.8	-	0.2	11	6.9	-	0.3	11	%, SEM
**Guerci et al. 1996 [108]**	5.9	0.6	14	6	0.6	14	5.9	0.63	-	14	6	0.6	-	14	%, SD
**Hanaire-Boutin et al. 1996 [101]**	7.5	0.8	18	7.6	0.8	18	7.5	-	0.2	18	7.6	-	0.2	18	%, SEM
**Duvillard et al. 2005/2007 [106, 107]**	7.2	1	7	7.3	0.9	7	7.24	1	-	7	7.34	0.94	-	7	%, SD
**Pacifico et al. 1997 [98]**	6.6	1.4	8	6.5	1.1	8	6.6	1.4	-	8	6.5	1.1	-	8	%, SD
**Walter et al. 1989 [99]**	8	0.5	6	7.9	0.5	6	8	0.5	-	6	7.9	0.5	-	6	%, SD
**Hedman et al. 2009/2014, Arnqvist et al. 2010 [110-112]**	8.6	1.4	10	7.9	0.8	20	8.6	1.4	-	10	7.9	0.8	-	20	%, SD

Legends: CSII, Continuous subcutaneous insulin infusion; CIPII, Continuous intraperitoneal insulin infusion; (–), no data; SD, standard deviation; SEM, standard error of means; SPSS, statistical software program; IDF, Ilze Dirnena-Fusini; *, data given as mean (min-max) (CIPII 7.6 (7.0 – 8.6); CSII 8.7 (7.0 – 9.5)); [κ], Authors of the study did not provide statistical term for difference (SD or SEM), decision to use SD or SEM was made by reproducing statistical test by using raw data from article.

Table S2.10. Data modification for STATA: SMBG.

Study ID	Data in forest plot, SMBG (mmol/L)						Original data								
	CIPII			CSII			CIPII				CSII				Unit
	Mean	SD	Total	Mean	SD	Total	Mean	SD	SEM	Total	Mean	SD	SEM	Total	
**Pitt et al. 1992 (data extracted from figure) [6]**	7.8	0.4	10	8.9	0.6	10	-	-	-	10	-	-	-	10	mg/dL, SEM
**Georgopoulos et al. 1992 [83]**	7.7	1.2	7	10.5	2	7	7.7	1.2	-	7	10.5	2	-	7	mM, SD
**Micossi et al. 1986 [84]**	8.8	1.3	6	9.7	1.4	6	8.8	-	0.55	6	9.68	-	0.58	6	mmol/L, SEM
**Beylot et al. 1987 (SD calculated in SPSS by IDF) [103]**	8.2	0.9	4	8.8	1.3	4	-	-	-	4	-	-	-	4	mmol/L
**Catargi et al. 2002 [88]**	8.1	1	14	8.5	0.9	14	145.4	18.3	-	14	153.3	17.3	-	14	mg/dL, SD
**Georgopoulos et al. 1994 [102]**	7.4	1.1	8	7.8	1.1	8	7.4	1.1	-	8	7.8	1.1	-	8	mmol/L, SD
**Guerci et al. 1996 [108]**	7.6	0.5	14	7.8	0.7	14	7.55	0.47	-	14	7.78	0.7	-	14	mmol/L, SD
**Raccah et al. 1994 [109]**	8	1.8	11	8.3	0.8	11	151	-	9.3	11	146	-	5.5	11	mg/dL, SEM
**Lassmann-Vague et al. 1994 [104]**	8.3	1.8	11	8.3	1.2	11	151	-	8	11	151	-	9	11	mg/dL, SEM

Legends: SMBG, self-monitoring of blood glucose; CSII, Continuous subcutaneous insulin infusion; CIPII, Continuous intraperitoneal insulin infusion; (–), no data; SD, standard deviation; SEM, standard error of means; SPSS, statistical software program; IDF, Ilze Dirnena-Fusini.

Table S2.11. Data modification for STATA: Insulin levels.

Study ID	Data in forest plot, insulin levels (pmol/L)						Original data								
	CIPII			CSII			CIPII				CSII				Unit
	Mean	SD	Total	Mean	SD	Total	Mean	SD	SEM	Total	Mean	SD	SEM	Total	
**Oskarsson et al. 1999 [90]**	28	5.8	7	48.1	20.9	7	28	-	2.2	7	48.1	-	7.9	7	pmol/L, SEM
**Oskarsson et al. 2000 [89]**	35.8	7.5	7	53.4	9.9	7	35.8	-	2.9	7	53.4	-	3.8	7	pmol/L, SEM
**Giacca et al. 1993 [100]**	30.8	13.6	5	45	23.3	5	30.8	-	6.1	5	45	-	10.4	5	pmol/L, SEM
**Beylot et al. 1987 [103]**	131.9	27.8	4	152.8	27.8	4	19	-	2	4	22	-	2	4	mU/L, SEM
**Colette et al. 1989 [114]**	115.3	67.6	13	141	103.6	11	16.6	-	2.7	13	20.3	-	4.5	11	µU/mL, SEM
**Lassmann-Vague et al. 1996 [87]**	60.4	23.1	11	66.7	30	11	8.7	-	1	11	9.6	-	1.3	11	mU/L, SEM
**Raccah et al. 1994 [109]**	100	71.4	11	118.1	89.9	11	14.4	-	3.1	11	17	-	3.9	11	mU/L, SEM
**Lassmann-Vague et al. 1994 [104]**	114.6	48.3	11	118.1	89.8	11	16.5	-	2.1	11	17	-	3.9	11	µU/mL, SEM

Legends: CSII, Continuous subcutaneous insulin infusion; CIPII, Continuous intraperitoneal insulin infusion; (–), no data; SD, standard deviation; SEM, standard error of means.

Table S2.12. Data modification for STATA: cholesterol levels.

Study ID	Data in forest plot, cholesterol levels (mmol/L)						Original data								
	CIPII			CSII			CIPII				CSII				Unit
	Mean	SD	Total	Mean	SD	Total	Mean	SD	SE	Total	Mean	SD	SE	Total	
**Duvillard et al. 2005/2007 [106, 107]**	5	0.6	7	5.4	0.7	7	5.04	0.58	-	7	5.36	0.72	-	7	mmol/L, SD
**Georgopoulos et al. 1994 [102]**	4.6	0.8	8	4.8	0.8	8	4.56	0.83	-	8	4.85	0.8	-	8	mmol/L, SD
**Georgopoulos et al. 1992 [83]**	4.6	1.1	7	4.9	1.3	7	4.6	1.1	-	7	4.9	1.3	-	7	mM, SD
**Raccah et al. 1994 [109]**	4.9	2.3	11	5	1.3	11	4.92	-	0.69	11	5.03	-	0.38	11	mM, SEM
**Guerci et al. 1996 [108]**	5	0.6	14	5	0.6	14	5.01	0.59	-	14	4.97	0.65	-	14	mmol/L, SD
**Pacifico et al. 1997 [98]**	4.8	0.8	8	4.7	0.8	8	185.8	31	-	8	182.5	33	-	8	mg/dL, SD
**Micossi et al. 1986 [84]**	5.1	1.2	6	4.4	0.9	6	5.1	-	0.5	6	4.4	-	0.38	6	mmol/L, SEM

Legends: CSII, Continuous subcutaneous insulin infusion; CIPII, Continuous intraperitoneal insulin infusion; (–), no data; SD, standard deviation; SEM, standard error of means.

Table S2.13. Data modification for STATA: triglyceride levels.

Study ID	Data in forest plot, triglyceride levels (mmol/L)						Original data								
	CIPII			CSII			CIPII				CSII				Unit
	Mean	SD	Total	Mean	SD	Total	Mean	SD	SE	Total	Mean	SD	SE	Total	
**Georgopoulos et al. 1992 [83]**	1.2	0.3	7	1.3	0.4	7	1.23	0.27	-	7	1.35	0.27	-	7	mM, SD
**Georgopoulos et al. 1994 [102]**	0.9	0.2	8	0.9	0.3	8	0.93	0.2	-	8	0.93	0.3	-	8	mmol/L, SD
**Raccah et al. 1994 [109]**	0.8	0.3	11	0.8	0.3	11	0.83	-	0.1	11	0.83	-	0.1	11	mM, SEM
**Guerci et al. 1996 [108]**	1.1	0.6	14	1.1	0.4	14	1.13	0.56	-	14	1.1	0.4	-	14	mmol/L, SD
**Pacifico et al. 1997 [98]**	0.9	0.3	8	0.8	0.3	8	77.6	25.6	-	8	71.6	27.6	-	8	mg/dL, SD
**Duvillard et al. 2005/2007 [106, 107]**	1.3	0.3	7	1.1	0.2	7	1.29	0.29	-	7	1.1	0.24	-	7	mmol/L, SD
**Micossi et al. 1986 [84]**	1.5	0.4	6	0.9	0.3	6	1.5	-	0.17	6	0.9	-	0.12	6	mmol/L, SEM

Legends: CSII, Continuous subcutaneous insulin infusion; CIPII, Continuous intraperitoneal insulin infusion; (–), no data; SD, standard deviation; SEM, standard error of means.

Table S2.14. Data modification for STATA: insulin requirement

Study ID	Data in forest plot, insulin requirement (U/24 hours)						Original data								
	CIPII			CSII			CIPII				CSII				Unit
	Mean	SD	Total	Mean	SD	Total	Mean	SD	SE	Total	Mean	SD	SE	Total	
**Micossi et al. 1986 [84]**	46.0	10.7	6	48.6	10.3	6	46.0	-	4.37	6	48.6	-	4.22	6	SEM, U/24h
**Liebl et al. 2009 [5]**	44.2	16.6	30	46	23.6	30	44.2	16.6	-	30	46	44.2	-	30	SD, U/24h
**Duvillard et al. 2005/2007 [106, 107]**	43.6	9.8	7	45	17.8	7	43.6	9.8	-	7	45	17.8	-	7	SD, U/24h
**Hanaire-Broutin et al. 1996 [101]**	39.1	10.6	18	39.6	8.9	18	39.1	-	2.5	18	39.6	-	2.1	18	SEM, U/24h
**Oskarsson et al. 2000 [89]**	37.9	7.1	7	38.2	10.3	7	37.9	-	2.7	7	38.2	-	3.9	7	SEM, U/24h
**Georgopoulos et al. 1994 [102]**	62.4	44.9	8	61.9	45.7	8	62.4	44.9	-	8	61.9	45.7	-	8	SD, U/24h
**Lassmann-Vague et al. 1994 [104]**	41.6	12.9	11	40	13.3	11	41.6	-	3.9	11	40	-	4	11	SEM, U/24h
**Pacifico et al. 1997 [98]**	42.8	6.6	8	40.8	8	8	42.8	6.6	-	8	40.8	8		8	SD, U/24h
**Oskarsson et al. 1999 [90]**	38.4	7.7	7	36.1	7.4	7	38.4	-	2.9	7	36.1	-	2.8	7	SEM, U/24h
**Raccah et al. 1994 [109]**	43.8	15.9	11	40.5	14.6	11	43.8	-	4.8	11	40.5	-	4.4	11	SEM, U/24h
**Jeandidier et al. 1992 [86]**	39	11	8	32	13	8	39	11	-	8	32	13	-	8	SD, U/24h
**Dassau et al. 2017***	43.7	0.1	10	32.3	0.1	10	43.7	0.08	-	10	32.3	0.05	-	10	SD, U/24h
**Hedman et al. 2009/2014, Arnqvist et al. 2010 [110-112]**	51.2	31.5	10	39.3	10.5	20	51.2	31.5	-	10	39.3	10.5	-	20	SD, U/24h

Legends: CSII, Continuous subcutaneous insulin infusion; CIPII, Continuous intraperitoneal insulin infusion; (–), no data; SD, standard deviation; SEM, standard error of means, Asterix (*), 24-hour measurements

Figure S1a. Meta-analysis of HbA1c (%) in patients during CIPII treatment compared to that during control treatment (CSII).

Study	Treatment N	Treatment Mean	Treatment SD	Control N	Control Mean	Control SD	Mean Diff. with 95% CI	Weight (%)
Georgopoulos et al. 1992	7	7.7	1.2	7	9.8	1.4	-2.10 [-3.47, -0.73]	3.44
Liebl et al. 2013/2014	10	7.2	0.5	10	8.8	1.2	-1.60 [-2.41, -0.79]	5.67
Oskarsson et al. 1999	7	7.1	0.5	7	8.5	0.8	-1.40 [-2.10, -0.70]	6.20
Oskarsson et al. 2000	7	7.2	0.5	7	8.6	1.1	-1.40 [-2.30, -0.50]	5.24
Schnell et al. 1994	5	8.5	0.5	5	9.8	0.7	-1.30 [-2.05, -0.55]	5.92
Pitt et al. 1992	10	8.0	1.8	10	9.1	2.2	-1.10 [-2.86, 0.66]	2.46
Wredling, Adamson et al. 1991	6	7.6	0.4	6	8.7	0.6	-1.10 [-1.68, -0.52]	6.81
Colette et al. 1989	13	8.0	1.4	11	8.9	2.0	-0.90 [-2.26, 0.46]	3.45
Georgopoulos et al. 1994	8	8.7	1.2	8	9.4	1.5	-0.70 [-2.03, 0.63]	3.55
Raccah et al. 1994	11	6.3	1.0	11	6.9	1.0	-0.60 [-1.44, 0.24]	5.52
Catargi et al. 2002	14	7.3	0.8	14	7.8	0.9	-0.50 [-1.13, 0.13]	6.54
Selam et al. 1989	6	8.2	1.4	8	8.6	1.3	-0.40 [-1.82, 1.02]	3.28
Lassmann-Vague et al. 1994	11	6.8	0.7	11	6.9	1.0	-0.10 [-0.82, 0.62]	6.08
Duvillard et al. 2005/2007	7	7.2	1.0	7	7.3	0.9	-0.10 [-1.10, 0.90]	4.79
Guerci et al. 1996	14	5.9	0.6	14	6.0	0.6	-0.10 [-0.54, 0.34]	7.45
Hanaire-Broutin et al. 1996	18	7.5	0.8	18	7.6	0.8	-0.10 [-0.62, 0.42]	7.08
Pacifico et al. 1997	8	6.6	1.4	8	6.5	1.1	0.10 [-1.13, 1.33]	3.87
Walter et al. 1989	6	8.0	0.5	6	7.9	0.5	0.10 [-0.47, 0.67]	6.87
Hedman et al. 2009/2014	10	8.6	1.4	20	7.9	0.8	0.70 [-0.08, 1.48]	5.78
**Overall**							-0.61 [-0.94, -0.28]	

Heterogeneity: $\tau^2 = 0.32$, $I^2 = 67.60\%$, $H^2 = 3.09$

Test of $\theta_i = \theta_j$: $Q(18) = 53.48$, $p = 0.00$

Test of $\theta = 0$: $t(18) = -3.67$, $p = 0.00$

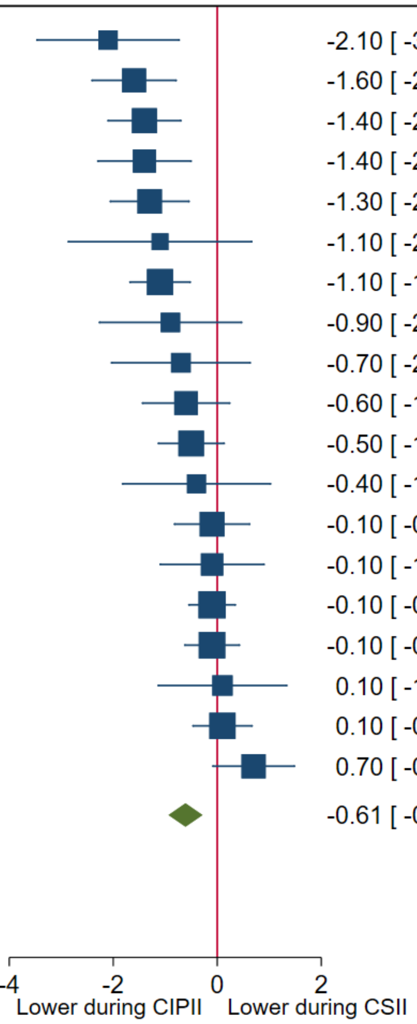

Legends: Treatment, continuous intraperitoneal insulin infusion; Control, continuous subcutaneous insulin infusion.

Figure S1b. Subgroup meta-analysis of HbA1c (%) according to duration in patients during CIPII treatment compared to that during control treatment (CSII).

Length of the CIPII-period (months)	Treatment N	Treatment Mean	Treatment SD	Control N	Control Mean	Control SD	Mean Diff. with 95% CI	Weight (%)
**3**								
Catargi et al. 2002	14	7.3	0.8	14	7.8	0.9	-0.50 [-1.13, 0.13]	6.54
Lassmann-Vague et al. 1994	11	6.8	0.7	11	6.9	1.0	-0.10 [-0.82, 0.62]	6.08
Duvillard et al. 2005/2007	7	7.2	1.0	7	7.3	0.9	-0.10 [-1.10, 0.90]	4.79
Walter et al. 1989	6	8.0	0.5	6	7.9	0.5	0.10 [-0.47, 0.67]	6.87
Heterogeneity: τ^2 = 0.00, I^2 = 0.00%, H^2 = 1.00							-0.14 [-0.49, 0.20]	
Test of $\theta_i = \theta_j$: Q(3) = 1.96, p = 0.58								
**4**								
Guerci et al. 1996	14	5.9	0.6	14	6.0	0.6	-0.10 [-0.54, 0.34]	7.45
Heterogeneity: τ^2 = 0.00, I^2 = .%, H^2 = .							-0.10 [-0.54, 0.34]	
Test of $\theta_i = \theta_j$: Q(0) = 0.00, p = .								
**6**								
Oskarsson et al. 2000	7	7.2	0.5	7	8.6	1.1	-1.40 [-2.30, -0.50]	5.24
Georgopoulos et al. 1994	8	8.7	1.2	8	9.4	1.5	-0.70 [-2.03, 0.63]	3.55
Selam et al. 1989	6	8.2	1.4	8	8.6	1.3	-0.40 [-1.82, 1.02]	3.28
Hedman et al. 2009/2014	10	8.6	1.4	20	7.9	0.8	0.70 [-0.08, 1.48]	5.78
Heterogeneity: τ^2 = 0.70, I^2 = 70.84%, H^2 = 3.43							-0.42 [-1.41, 0.57]	
Test of $\theta_i = \theta_j$: Q(3) = 12.43, p = 0.01								
**10**								
Raccah et al. 1994	11	6.3	1.0	11	6.9	1.0	-0.60 [-1.44, 0.24]	5.52
Heterogeneity: τ^2 = 0.00, I^2 = .%, H^2 = .							-0.60 [-1.44, 0.24]	
Test of $\theta_i = \theta_j$: Q(0) = 0.00, p = .								
**11**								
Oskarsson et al. 1999	7	7.1	0.5	7	8.5	0.8	-1.40 [-2.10, -0.70]	6.20
Heterogeneity: τ^2 = 0.00, I^2 = .%, H^2 = .							-1.40 [-2.10, -0.70]	
Test of $\theta_i = \theta_j$: Q(0) = 0.00, p = .								
**12**								
Georgopoulos et al. 1992	7	7.7	1.2	7	9.8	1.4	-2.10 [-3.47, -0.73]	3.44
Schnell et al. 1994	5	8.5	0.5	5	9.8	0.7	-1.30 [-2.05, -0.55]	5.92
Hanaire-Broutin et al. 1996	18	7.5	0.8	18	7.6	0.8	-0.10 [-0.62, 0.42]	7.08
Pacifico et al. 1997	8	6.6	1.4	8	6.5	1.1	0.10 [-1.13, 1.33]	3.87
Heterogeneity: τ^2 = 0.67, I^2 = 77.40%, H^2 = 4.43							-0.79 [-1.72, 0.15]	
Test of $\theta_i = \theta_j$: Q(3) = 12.66, p = 0.01								
**13**								
Colette et al. 1989	13	8	1.4	11	8.9	2.0	-0.90 [-2.26, 0.46]	3.45
Heterogeneity: τ^2 = 0.00, I^2 = .%, H^2 = .							-0.90 [-2.26, 0.46]	
Test of $\theta_i = \theta_j$: Q(0) = 0.00, p = .								
**18**								
Pitt et al. 1992	10	8	1.8	10	9.1	2.2	-1.10 [-2.86, 0.66]	2.46
Heterogeneity: τ^2 = 0.00, I^2 = .%, H^2 = .							-1.10 [-2.86, 0.66]	
Test of $\theta_i = \theta_j$: Q(0) = -0.00, p = .								
**18.6**								
Wredling, Adamson et al. 1991	6	7.6	0.4	6	8.7	0.6	-1.10 [-1.68, -0.52]	6.81
Heterogeneity: τ^2 = 0.00, I^2 = .%, H^2 = .							-1.10 [-1.68, -0.52]	
Test of $\theta_i = \theta_j$: Q(0) = -0.00, p = .								
**24**								
Liebl et al. 2013/2014	10	7.2	0.5	10	8.8	1.2	-1.60 [-2.41, -0.79]	5.67
Heterogeneity: τ^2 = 0.00, I^2 = .%, H^2 = .							-1.60 [-2.41, -0.79]	
Test of $\theta_i = \theta_j$: Q(0) = 0.00, p = .								
**Overall**							-0.61 [-0.94, -0.28]	
Heterogeneity: τ^2 = 0.32, I^2 = 67.60%, H^2 = 3.09								
Test of $\theta_i = \theta_j$: Q(18) = 53.48, p = 0.00								
Test of group differences: $Q_b(9)$ = 26.00, p = 0.00								

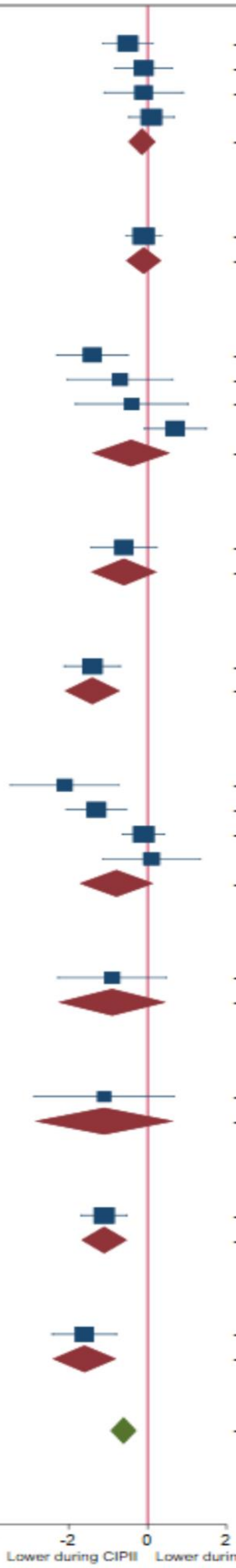

Legends: Treatment, continuous intraperitoneal insulin infusion (CIPII); Control, continuous subcutaneous insulin infusion (CSII).

Figure S1c. Subgroup meta-analysis of HbA1c (%) in patients during CIPII treatment compared to that during control treatment (CSII).

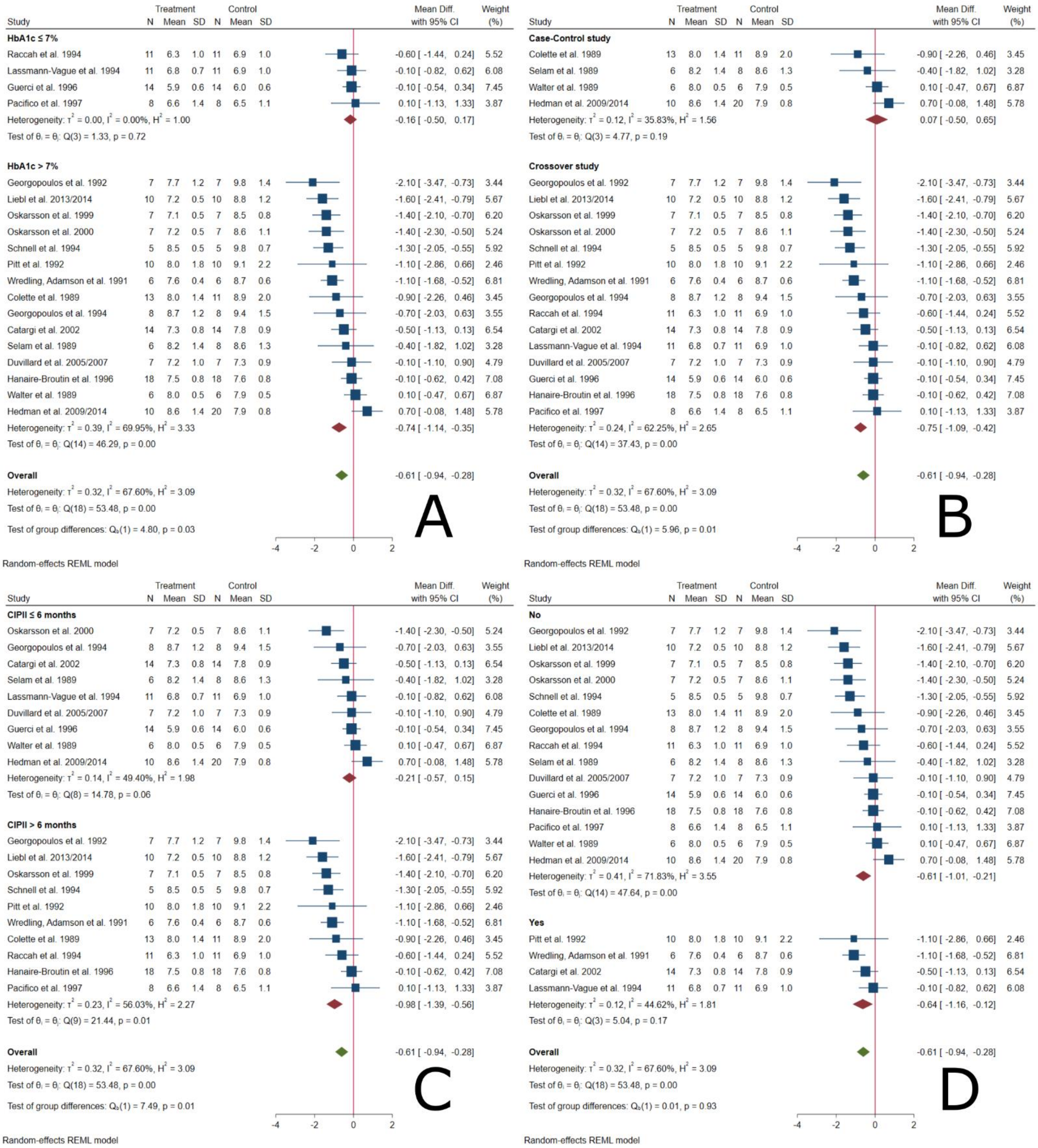

Legends: Treatment, continuous intraperitoneal insulin infusion (CIPII); Control, continuous subcutaneous insulin infusion (CSII). Figure A: Subgroup analysis according to HbA1c levels before starting CIPII treatment (≤ 7 % and > 7 %); Figure B: Subgroup analysis according to study type (Case-Control studies and Crossover studies); Figure C: Subgroup analysis according to length of the CIPII-period (≤ 6 months and > 6 months); Figure D: Subgroup analysis according to whether or not there was an additional controlled CSII follow-up-period with subsequent CIPII-period.

Figure S1d. Overall subgroup meta-analysis of HbA1c (%) in patients during CIPII treatment compared to that during control treatment (CSII).

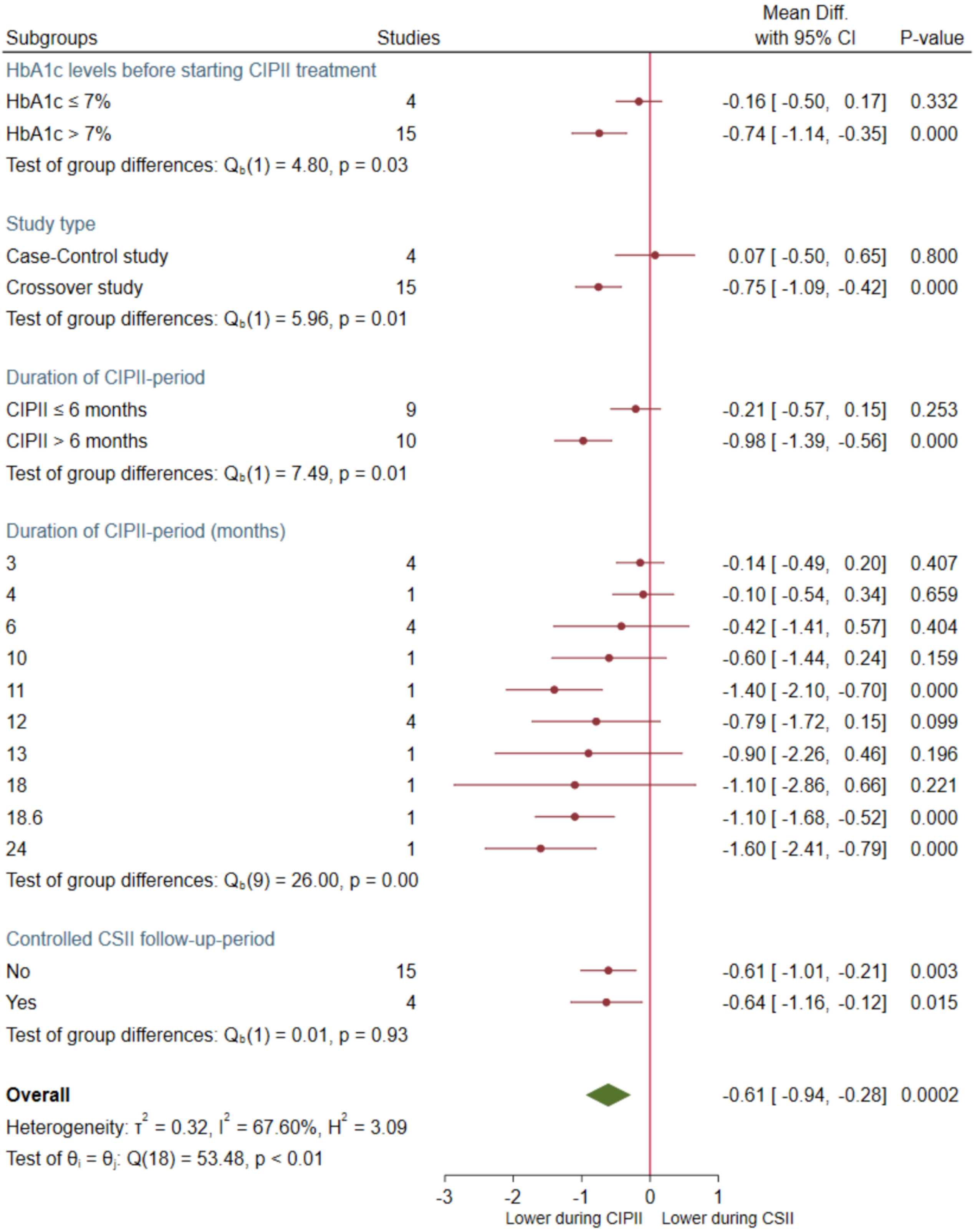

Legends: CIPII, continuous intraperitoneal insulin infusion; CSII, continuous subcutaneous insulin infusion.

Figure S1e. Meta-regression analysis bubble-plot of HbA1c (%) in patients during CIPII treatment compared to that during control treatment (CSII).

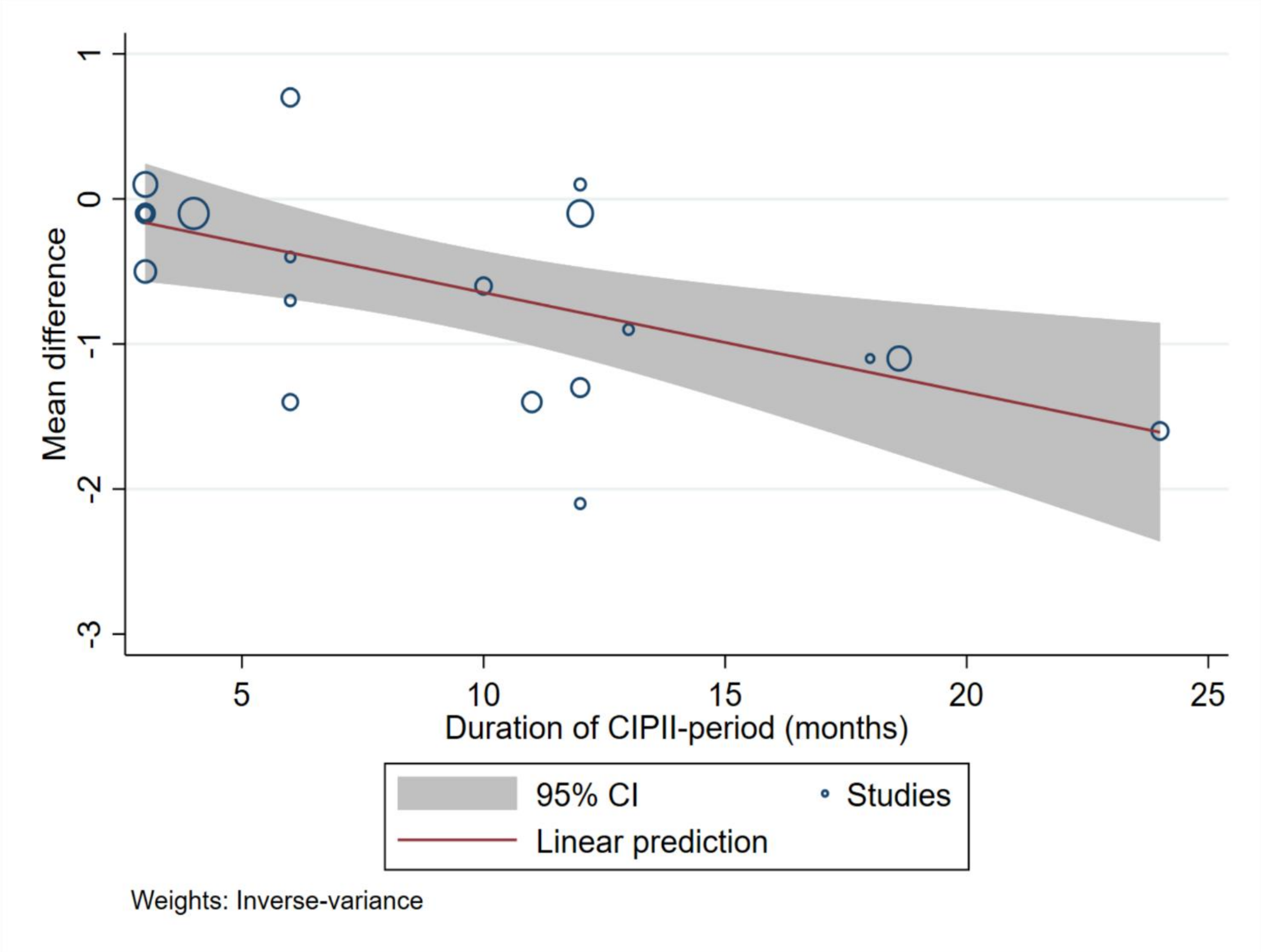

Figure S1f. Cumulative meta-analysis of HbA1c (%) in patients during CIPII treatment compared to that during control treatment (CSII) according to duration of CIPII treatment.

Study	Mean Difference with 95% CI	P-value	Duration of CIPII-period (months)
Catargi et al. 2002	-0.50 [-1.13, 0.13]	0.120	3
Lassmann-Vague et al. 1994	-0.33 [-0.80, 0.15]	0.178	3
Duvillard et al. 2005/2007	-0.28 [-0.71, 0.14]	0.193	3
Walter et al. 1989	-0.14 [-0.49, 0.20]	0.407	3
Guerci et al. 1996	-0.13 [-0.40, 0.14]	0.355	4
Oskarsson et al. 2000	-0.28 [-0.64, 0.08]	0.123	6
Georgopoulos et al. 1994	-0.30 [-0.64, 0.04]	0.081	6
Selam et al. 1989	-0.30 [-0.62, 0.02]	0.064	6
Hedman et al. 2009/2014	-0.21 [-0.57, 0.15]	0.253	6
Raccah et al. 1994	-0.24 [-0.58, 0.09]	0.149	10
Oskarsson et al. 1999	-0.38 [-0.76, 0.01]	0.054	11
Georgopoulos et al. 1992	-0.48 [-0.89, -0.07]	0.023	12
Schnell et al. 1994	-0.55 [-0.95, -0.15]	0.008	12
Hanaire-Broutin et al. 1996	-0.50 [-0.87, -0.13]	0.008	12
Pacifico et al. 1997	-0.47 [-0.83, -0.12]	0.009	12
Colette et al. 1989	-0.49 [-0.83, -0.15]	0.005	13
Pitt et al. 1992	-0.50 [-0.84, -0.17]	0.003	18
Wredling, Adamson et al. 1991	-0.55 [-0.87, -0.23]	0.001	18.6
Liebl et al. 2013/2014	-0.61 [-0.94, -0.28]	0.000	24

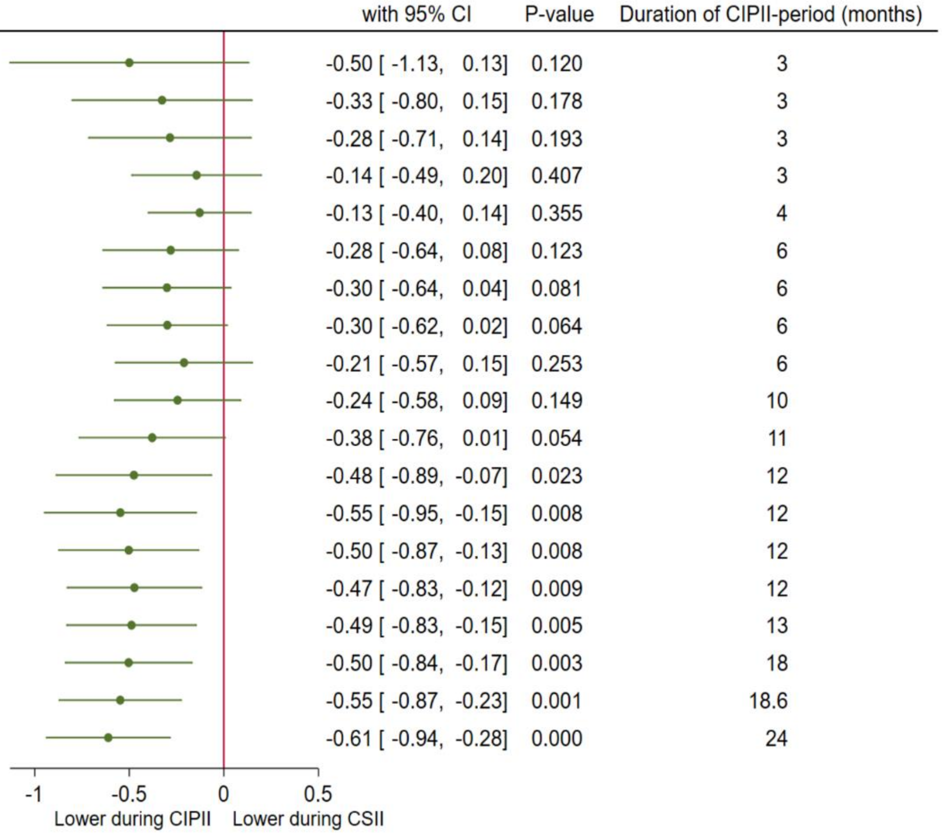

Legends: Treatment, continuous intraperitoneal insulin infusion (CIPII); Control, continuous subcutaneous insulin infusion (CSII).

Figure S2a. Subgroup meta-analysis of fasting blood glucose (mmol/L) in patients during CIPII treatment compared to that during control treatment (CSII).

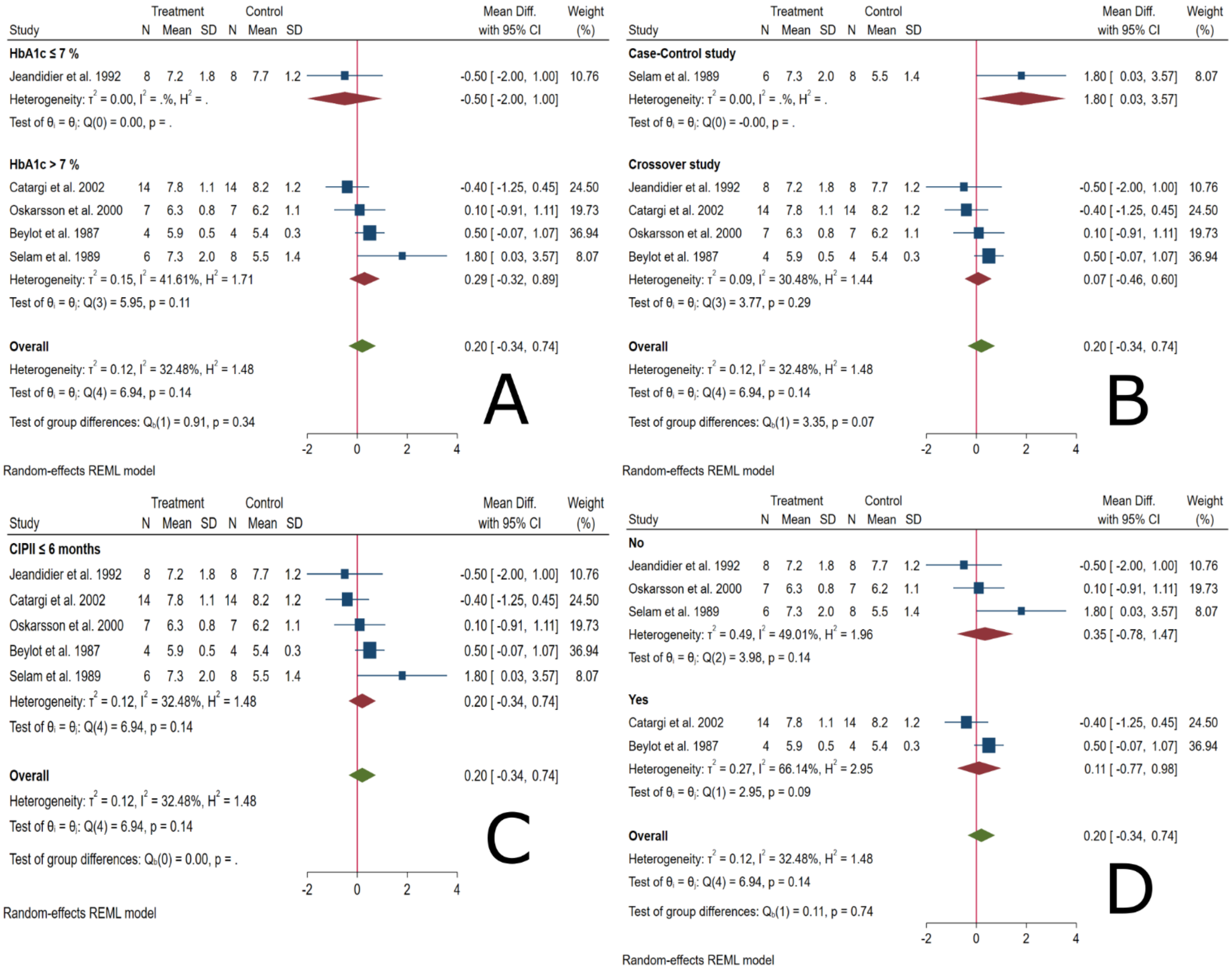

Legends: Treatment, continuous intraperitoneal insulin infusion (CIPII); Control, continuous subcutaneous insulin infusion (CSII). Figure A: Subgroup analysis according to HbA1c levels before starting CIPII treatment (≤ 7 % and > 7 %); Figure B: Subgroup analysis according to study type (Case-Control studies and Crossover studies); Figure C: Subgroup analysis according to length of the CIPII-period (≤ 6 months and > 6 months); Figure D: Subgroup analysis according to whether or not there was an additional controlled CSII follow-up-period with subsequent CIPII-period.

Figure S2b. Summarised subgroup meta-analysis of fasting blood glucose (mmol/L) in patients during CIPII treatment compared to that during control treatment (CSII).

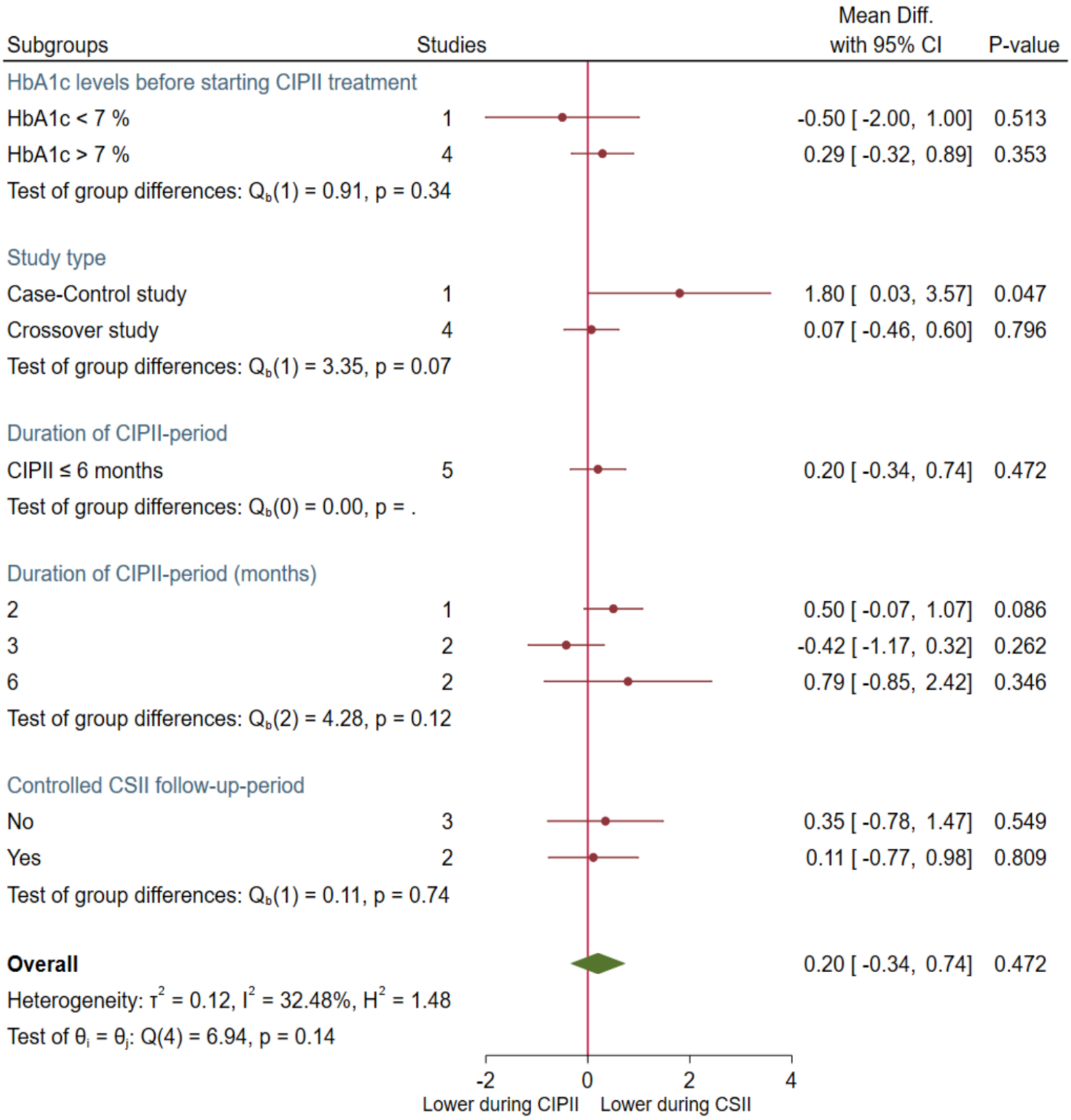

Legends: CIPII, continuous intraperitoneal insulin infusion; CSII, continuous subcutaneous insulin infusion.

Figure S3a. Subgroup meta-analysis of fasting insulin (pmol/L in patients during CIPII treatment compared to that during control treatment (CSII).

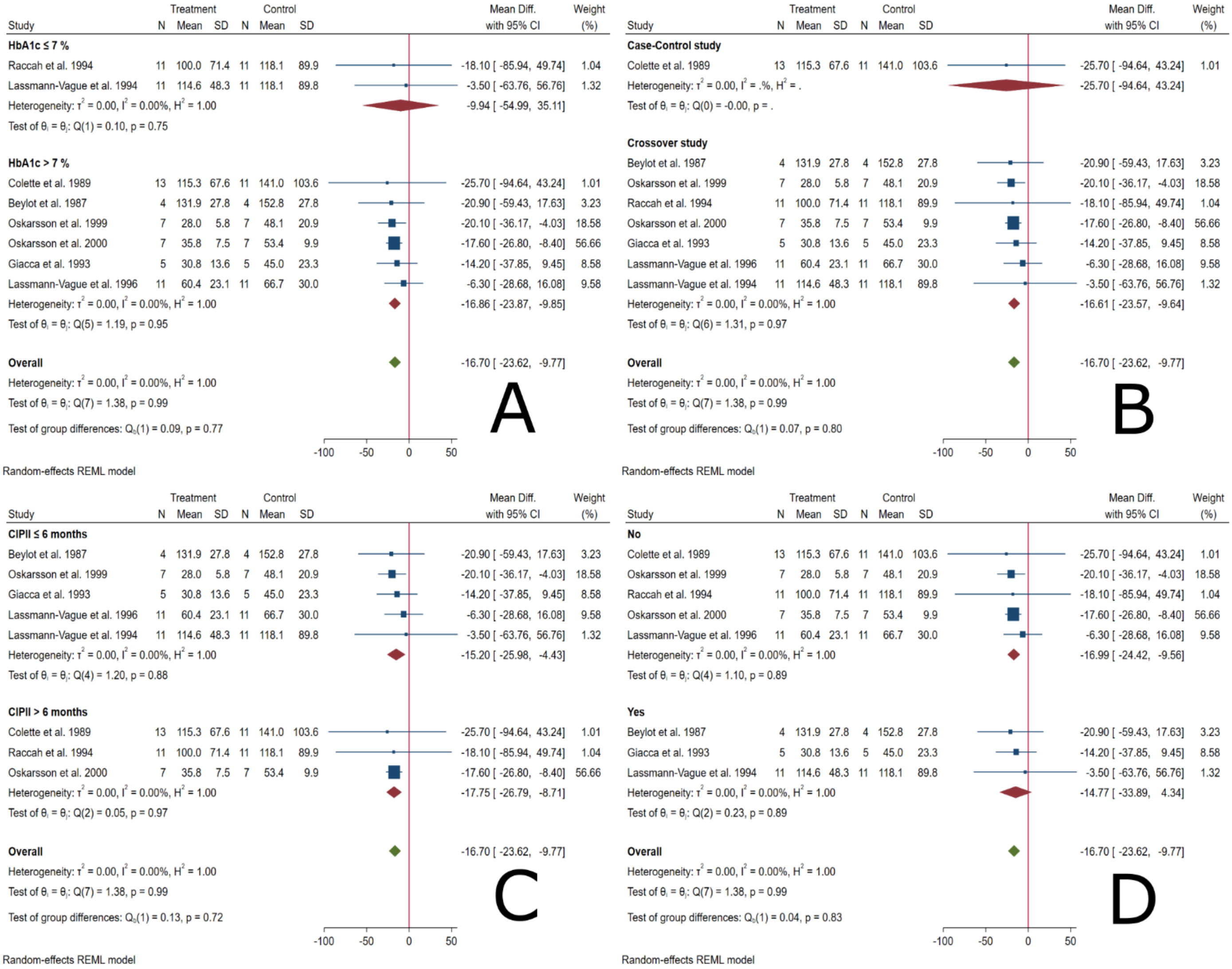

Legends: Treatment, continuous intraperitoneal insulin infusion (CIPII); Control, continuous subcutaneous insulin infusion (CSII). Figure A: Subgroup analysis according to HbA1c levels before starting CIPII treatment (≤ 7 % and > 7 %); Figure B: Subgroup analysis according to study type (Case-Control studies and Crossover studies); Figure C: Subgroup analysis according to length of the CIPII-period (≤ 6 months and > 6 months); Figure D: Subgroup analysis according to whether or not there was an additional controlled CSII follow-up-period with subsequent CIPII-period.

Figure S3b. Summarised subgroup meta-analysis of fasting insulin (pmol/L) in patients during CIPII treatment compared to that during control treatment (CSII).

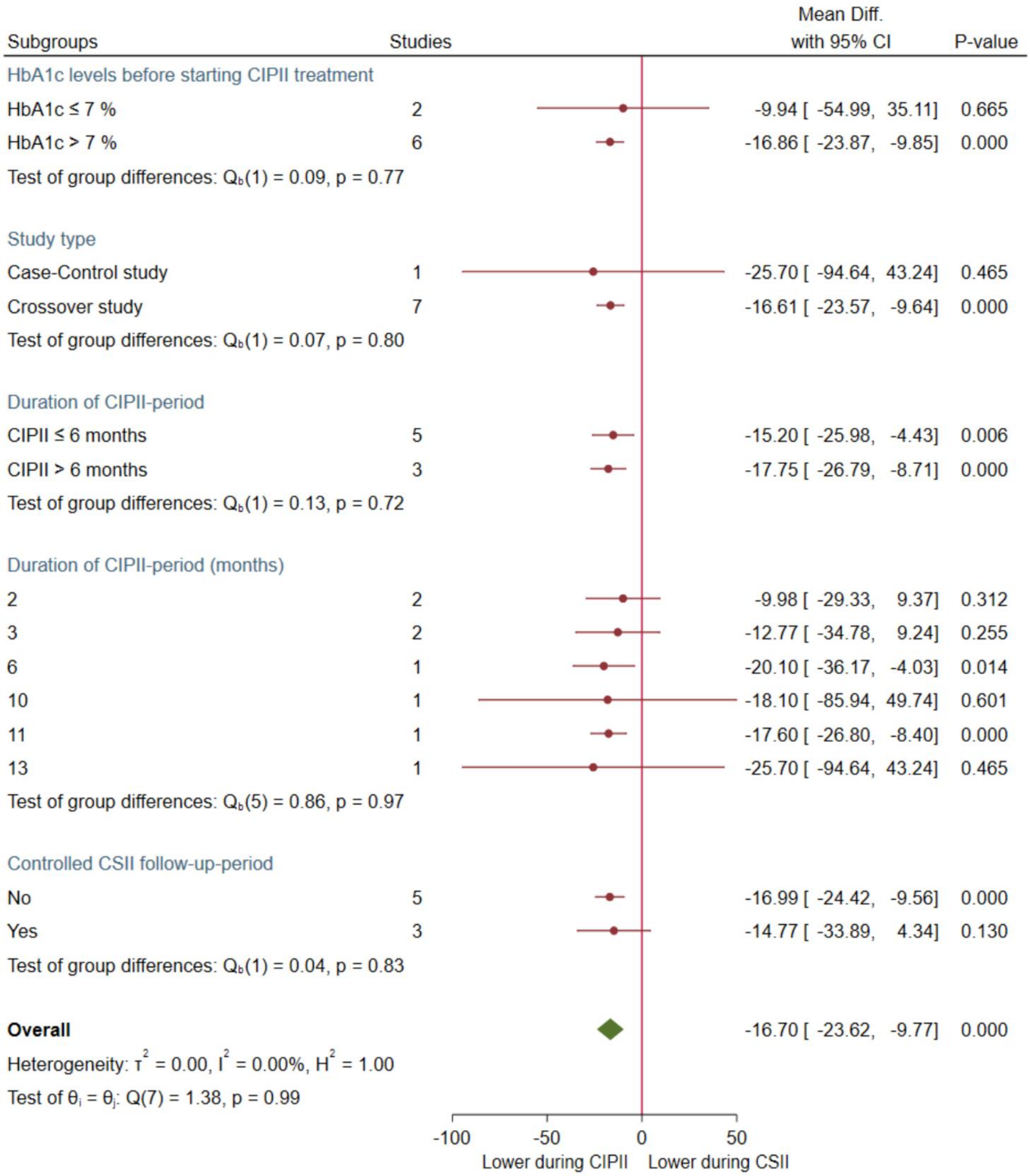

Legends: CIPII, continuous intraperitoneal insulin infusion; CSII, continuous subcutaneous insulin infusion.

Figure S4a. Subgroup meta-analysis of daily insulin dose (U/24 hours) in patients during CIPII treatment compared to that during control treatment (CSII).

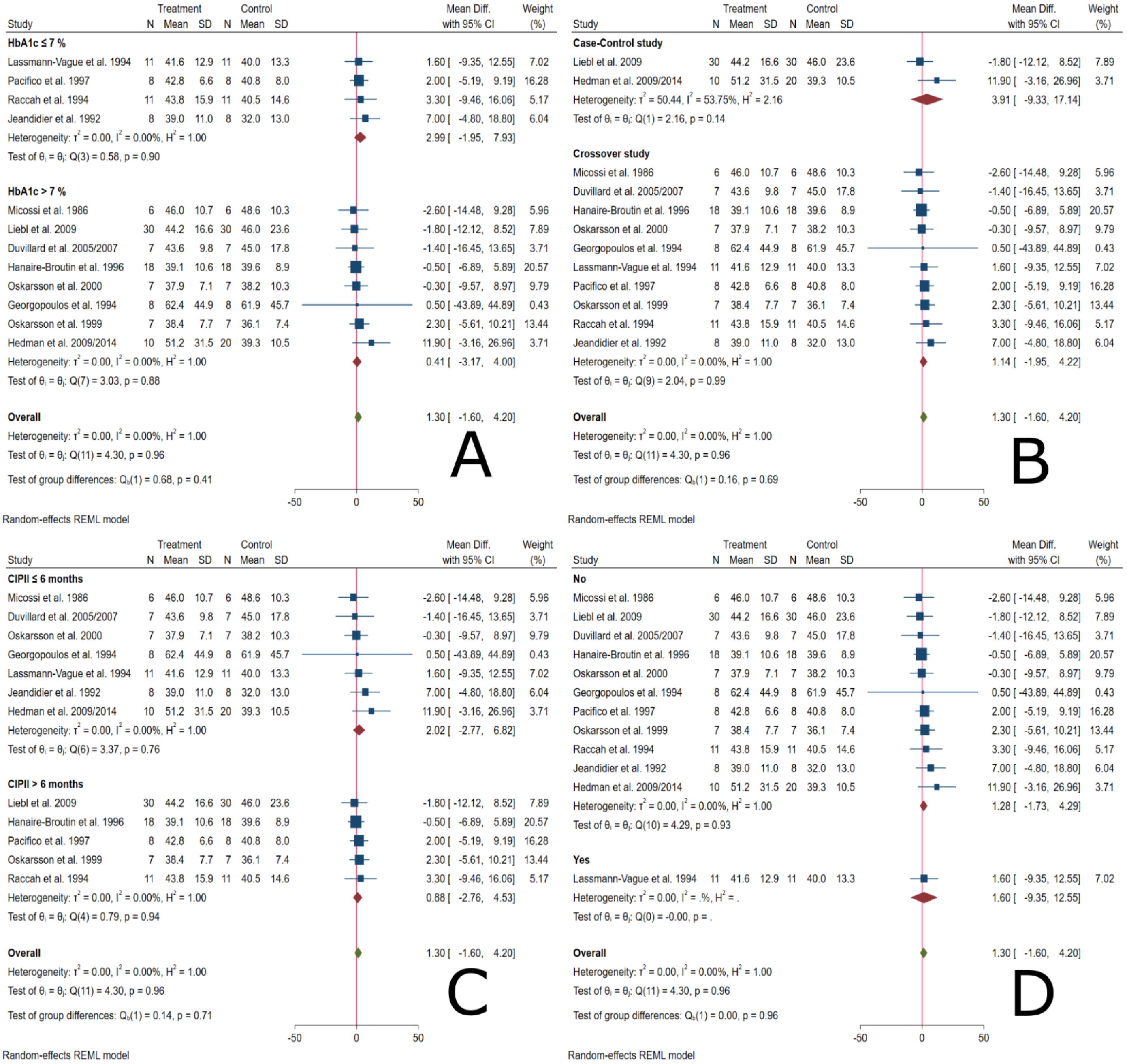

Legends: Treatment, continuous intraperitoneal insulin infusion (CIPII); Control, continuous subcutaneous insulin infusion (CSII). Figure A: Subgroup analysis according to HbA1c levels before starting CIPII treatment (≤ 7 % and > 7 %); Figure B: Subgroup analysis according to study type (Case-Control studies and Crossover studies); Figure C: Subgroup analysis according to length of the CIPII-period (≤ 6 months and > 6 months); Figure D: Subgroup analysis according to whether or not there was an additional controlled CSII follow-up-period with subsequent CIPII-period.

Figure S4b. Summarised subgroup meta-analysis of daily insulin dose (U/24 hours) in patients during CIPII treatment compared to that during control treatment (CSII).

Subgroups	Studies	Mean Diff. with 95% CI	P-value
HbA1c levels before starting CIPII treatment			
HbA1c ≤ 7 %	4	2.99 [-1.95, 7.93]	0.235
HbA1c > 7 %	8	0.41 [-3.17, 4.00]	0.821
Test of group differences: $Q_b(1) = 0.68$, $p = 0.41$			
Study type			
Case-Control study	2	3.91 [-9.33, 17.14]	0.563
Crossover study	10	1.14 [-1.95, 4.22]	0.471
Test of group differences: $Q_b(1) = 0.16$, $p = 0.69$			
Duration of CIPII-period			
CIPII ≤ 6 months	7	2.02 [-2.77, 6.82]	0.407
CIPII > 6 months	5	0.88 [-2.76, 4.53]	0.634
Test of group differences: $Q_b(1) = 0.14$, $p = 0.71$			
Duration of CIPII-period (months)			
1.5	1	-2.60 [-14.48, 9.28]	0.668
3	3	2.88 [-4.20, 9.96]	0.425
6	3	3.83 [-6.38, 14.04]	0.462
10	1	3.30 [-9.46, 16.06]	0.612
11	1	2.30 [-5.61, 10.21]	0.569
12	3	0.18 [-4.15, 4.52]	0.935
Test of group differences: $Q_b(5) = 1.25$, $p = 0.94$			
Controlled CSII follow-up-period			
No	11	1.28 [-1.73, 4.29]	0.404
Yes	1	1.60 [-9.35, 12.55]	0.775
Test of group differences: $Q_b(1) = 0.00$, $p = 0.96$			
**Overall**		1.30 [-1.60, 4.20]	0.379
Heterogeneity: $\tau^2 = 0.00$, $I^2 = 0.00\%$, $H^2 = 1.00$			
Test of $\theta_i = \theta_j$: $Q(11) = 4.30$, $p = 0.96$			

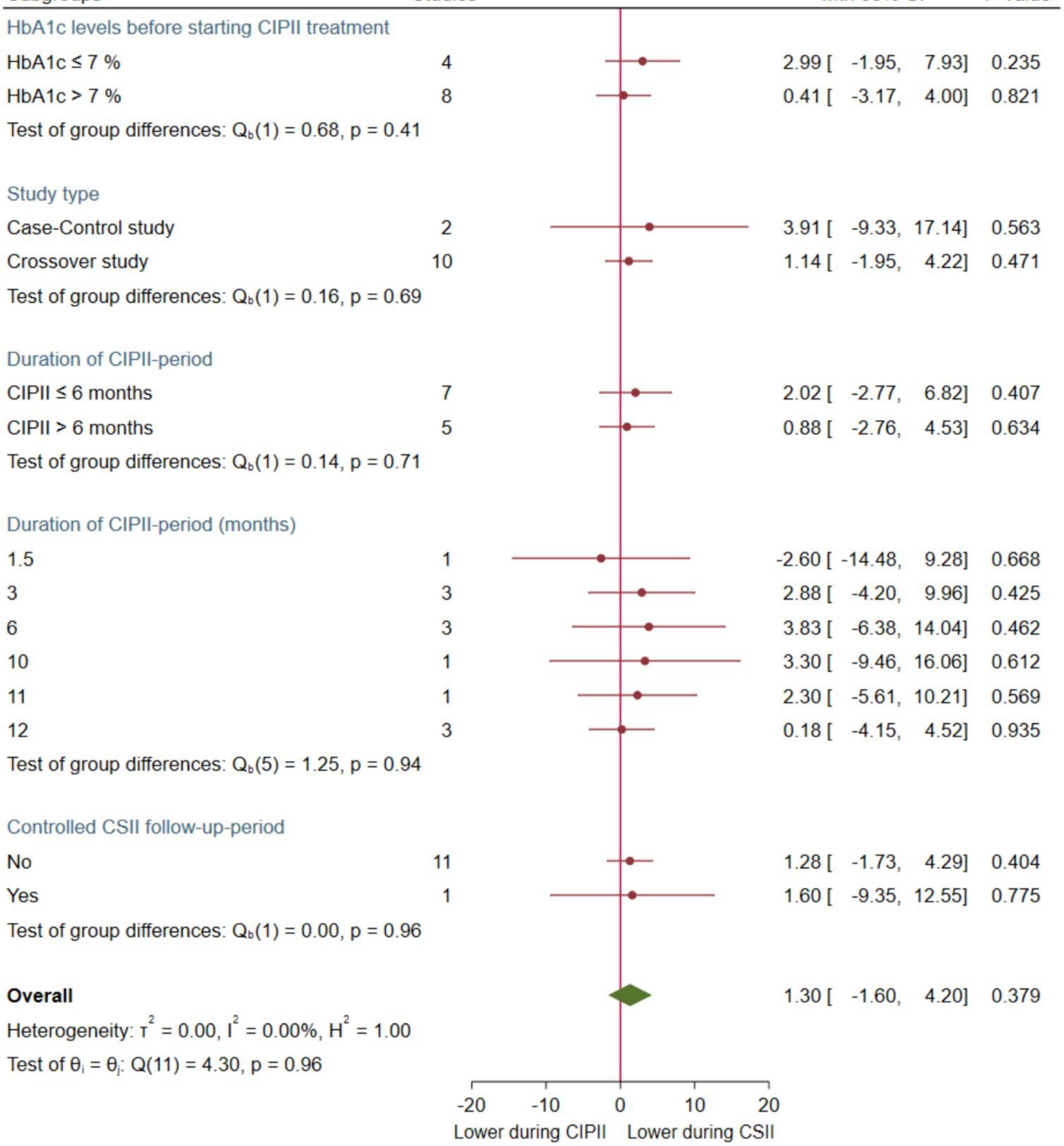

Legends: CIPII, continuous intraperitoneal insulin infusion; CSII, continuous subcutaneous insulin infusion.

Figure S5a. Meta-analysis of SMBG (mmol/L) in patients during CIPII treatment compared to that during control treatment (CSII).

	Treatment			Control			Mean Diff.	Weight
Study	N	Mean	SD	N	Mean	SD	with 95% CI	(%)
Pitt et al. 1992	10	7.8	0.4	10	8.9	0.6	-1.10 [-1.55, -0.65]	22.27
Georgopoulos et al. 1992	7	7.7	1.2	7	10.5	2.0	-2.80 [-4.53, -1.07]	4.42
Micossi et al. 1986	6	8.8	1.3	6	9.7	1.4	-0.90 [-2.43, 0.63]	5.43
Beylot et al. 1987	4	8.2	0.9	4	8.8	1.3	-0.60 [-2.15, 0.95]	5.31
Catargi et al. 2002	14	8.1	1.0	14	8.5	0.9	-0.40 [-1.10, 0.30]	15.57
Georgopoulos et al. 1994	8	7.4	1.1	8	7.8	1.1	-0.40 [-1.48, 0.68]	9.29
Guerci et al. 1996	14	7.6	0.5	14	7.8	0.7	-0.20 [-0.65, 0.25]	22.17
Raccah et al. 1994	11	8.0	1.8	11	8.3	0.8	-0.30 [-1.46, 0.86]	8.32
Lassmann-Vague et al. 1994	11	8.3	1.8	11	8.3	1.2	0.00 [-1.28, 1.28]	7.23
**Overall**							-0.62 [-1.01, -0.23]	

Heterogeneity: $\tau^2 = 0.13$, $I^2 = 41.73\%$, $H^2 = 1.72$

Test of $\theta_i = \theta_j$: Q(8) = 15.74, p = 0.05

Test of $\theta = 0$: t(8) = -3.09, p = 0.002

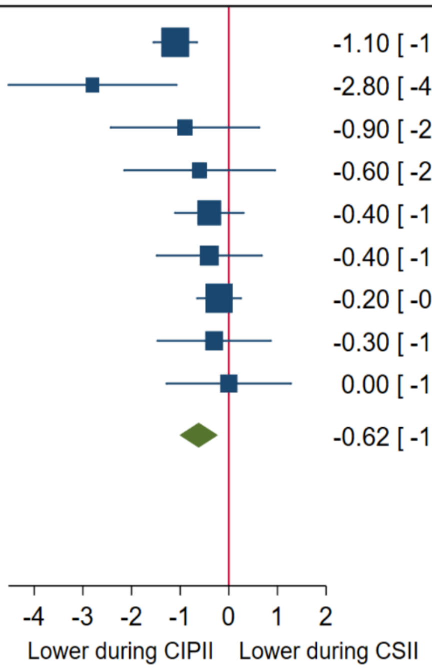

Legends: Treatment, continuous intraperitoneal insulin infusion (CIPII); Control, continuous subcutaneous insulin infusion (CSII); SMBG, self-monitoring of blood glucose.

Figure S5b. Subgroup meta-analysis of SMBG (mmol/L) in patients during CIPII treatment compared to that during control treatment (CSII).

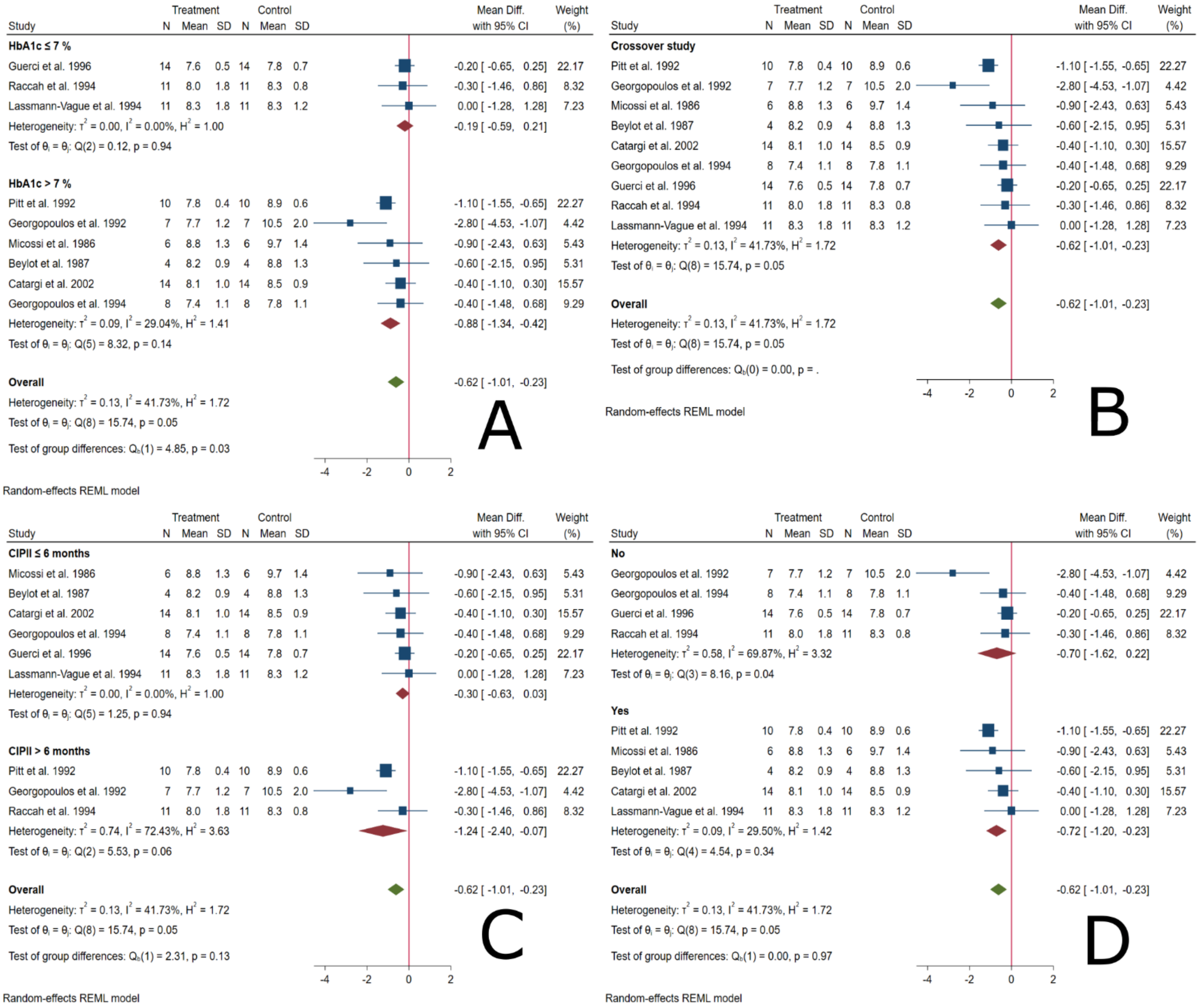

Legends: Treatment, continuous intraperitoneal insulin infusion (CIPII); Control, continuous subcutaneous insulin infusion (CSII). Figure A: Subgroup analysis according to HbA1c levels before starting CIPII treatment (≤ 7 % and > 7 %); Figure B: Subgroup analysis according to study type (Case-Control studies and Crossover studies); Figure C: Subgroup analysis according to length of the CIPII-period (≤ 6 months and > 6 months); Figure D: Subgroup analysis according to whether or not there was an additional controlled CSII follow-up-period with subsequent CIPII-period.

Figure S5c. Summarised subgroup meta-analysis of SMBG (mmol/L) in patients during CIPII treatment compared to that during control treatment (CSII).

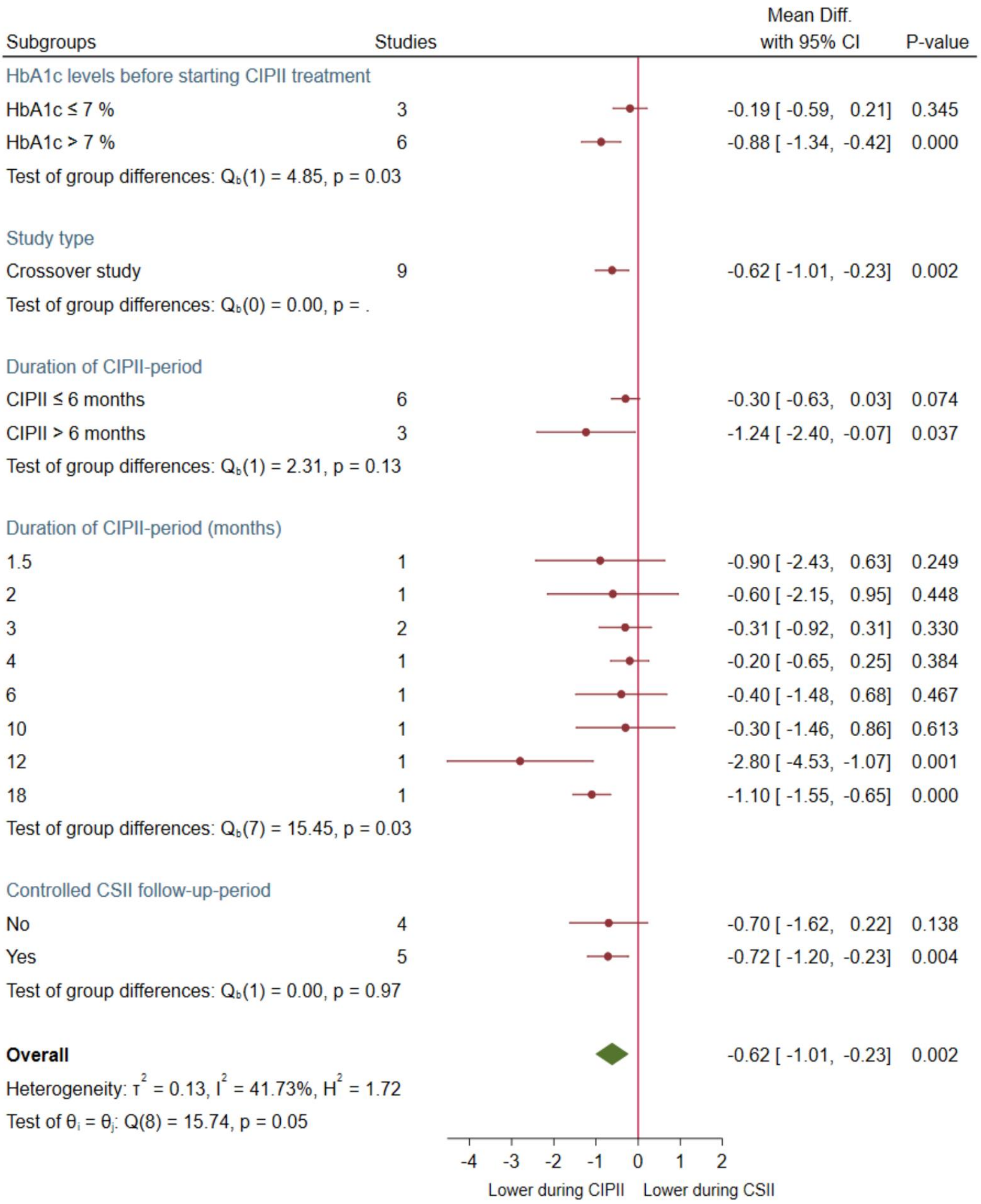

Legends: CIPII, continuous intraperitoneal insulin infusion; CSII, continuous subcutaneous insulin infusion.

Figure S6a. Meta-analysis of cholesterol (mmol/L) in patients during CIPII treatment compared to that during control treatment (CSII).

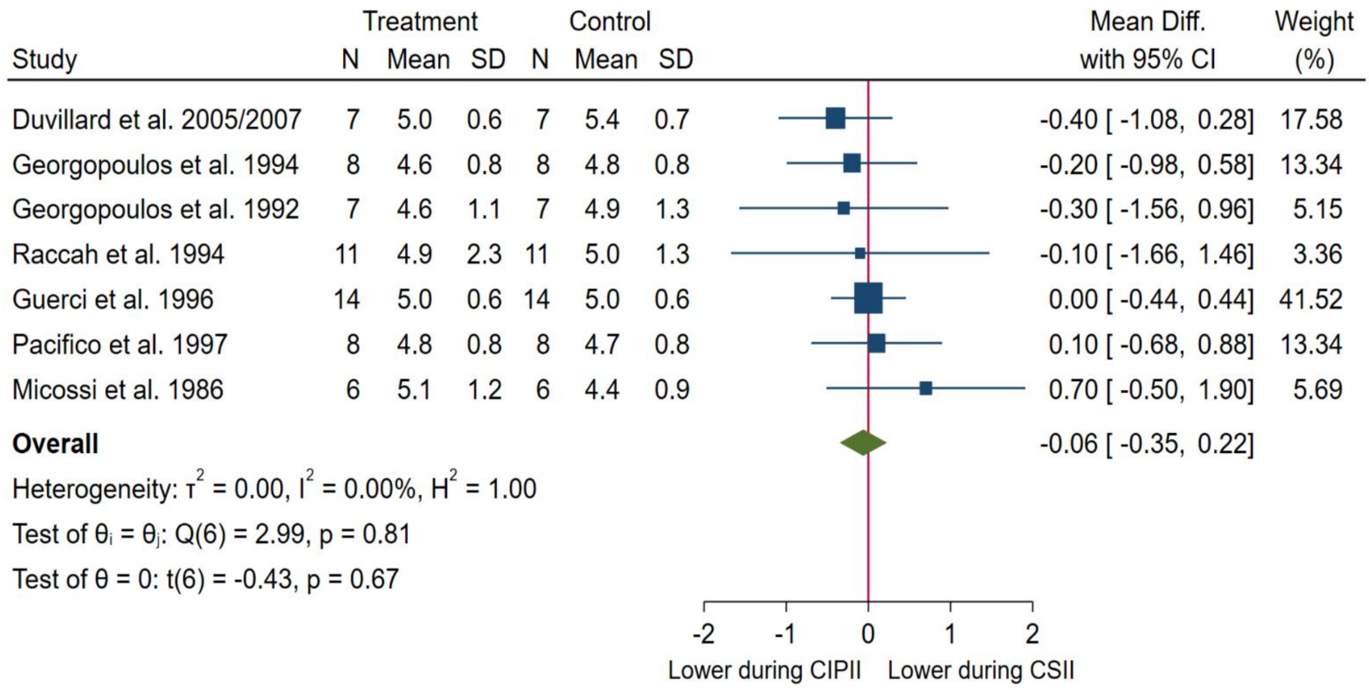

Legends: Treatment, continuous intraperitoneal insulin infusion (CIPII); Control, continuous subcutaneous insulin infusion (CSII).

Figure S6b. Subgroup meta-analysis of cholesterol (mmol/L) in patients during CIPII treatment compared to that during control treatment (CSII).

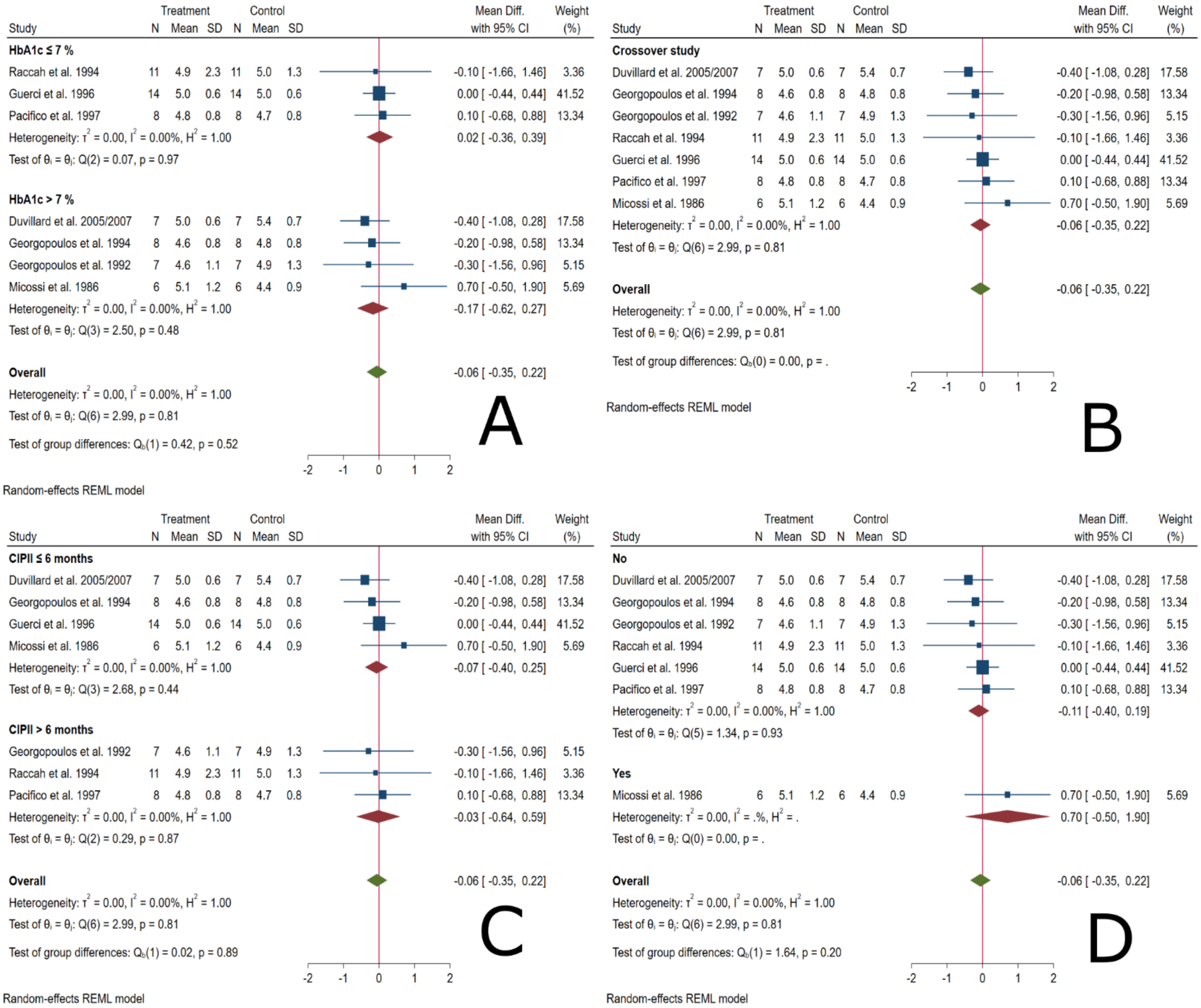

Legends: Treatment, continuous intraperitoneal insulin infusion (CIPII); Control, continuous subcutaneous insulin infusion (CSII). Figure A: Subgroup analysis according to HbA1c levels before starting CIPII treatment (≤ 7 % and > 7 %); Figure B: Subgroup analysis according to study type (Case-Control studies and Crossover studies); Figure C: Subgroup analysis according to length of the CIPII-period (≤ 6 months and > 6 months); Figure D: Subgroup analysis according to whether or not there was an additional controlled CSII follow-up-period with subsequent CIPII-period.

Figure S6c. Summarised subgroup meta-analysis of cholesterol (mmol/L) in patients during CIPII treatment compared to that during control treatment (CSII).

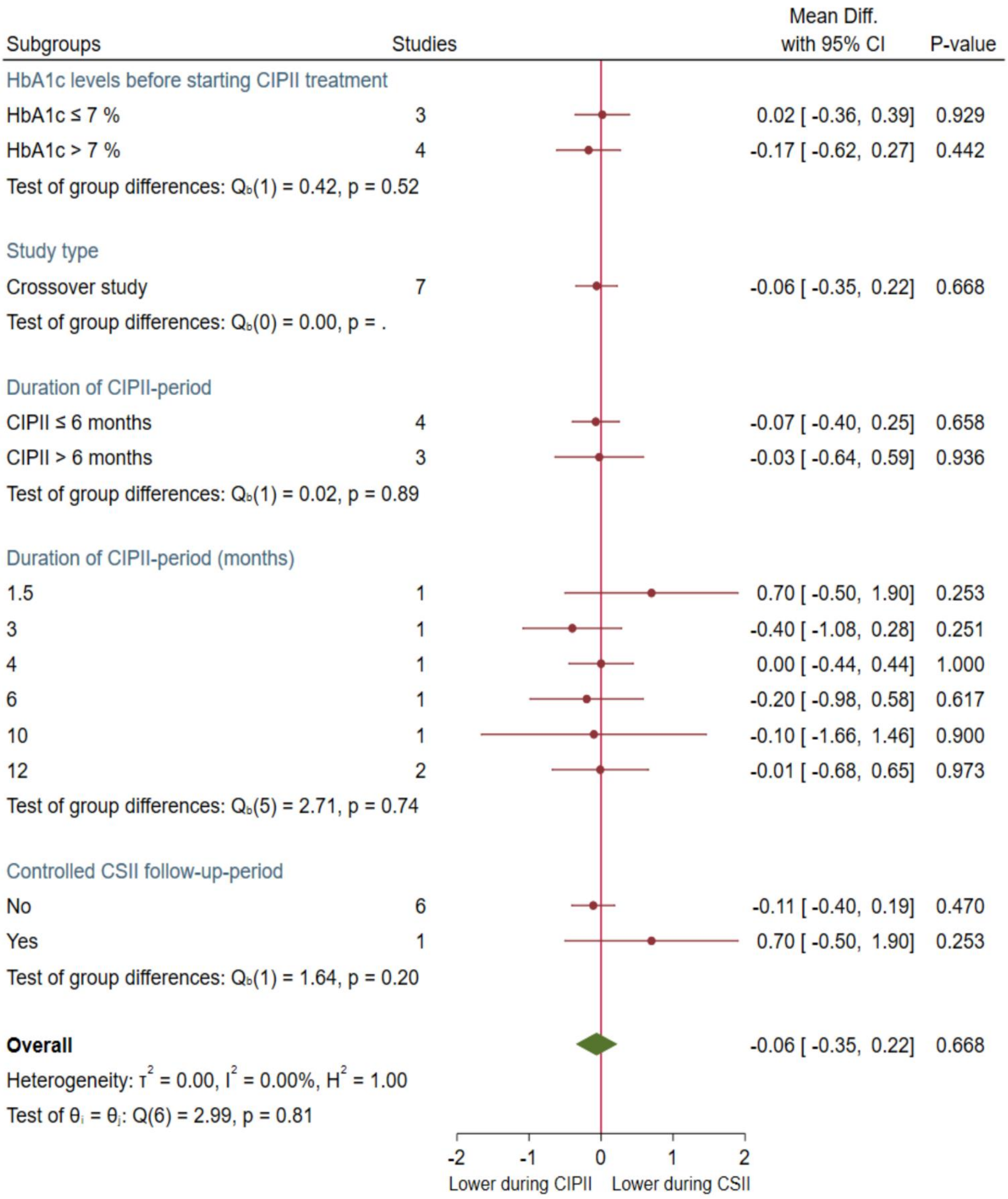

Legends: CIPII, continuous intraperitoneal insulin infusion; CSII, continuous subcutaneous insulin infusion.

Figure S7a. Meta-analysis of triglycerides (mmol/L) in patients during CIPII treatment compared to that during control treatment (CSII).

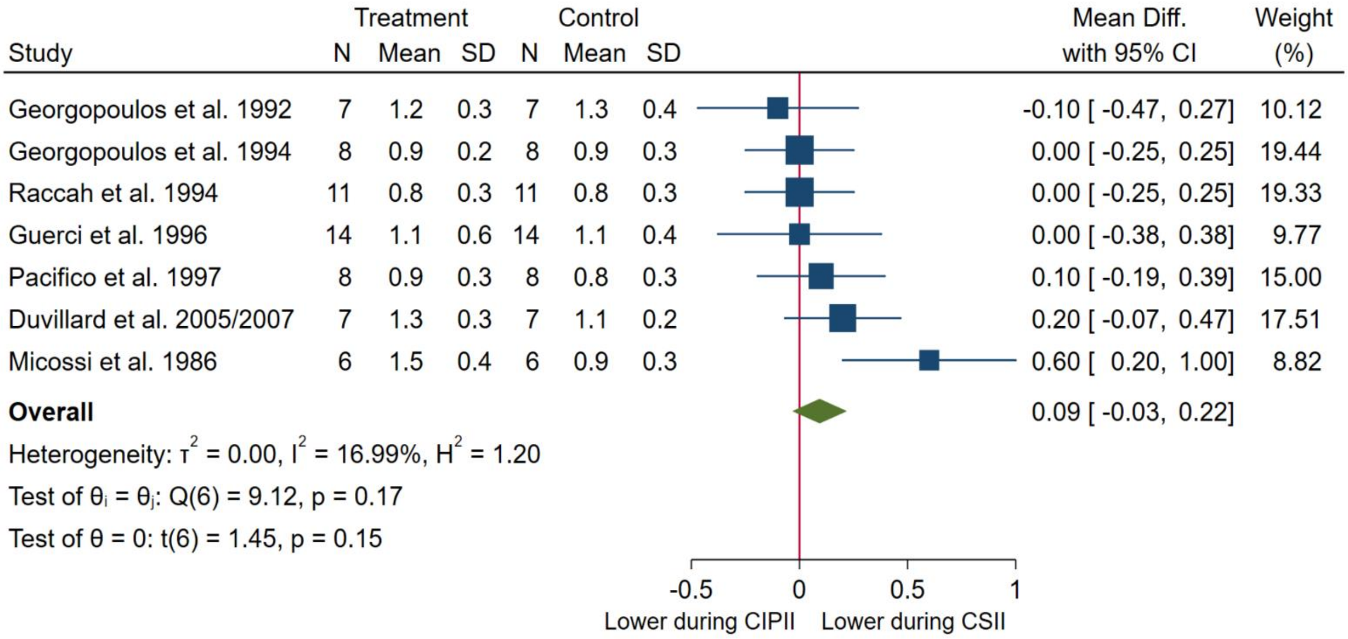

Legends: Treatment, continuous intraperitoneal insulin infusion (CIPII); Control, continuous subcutaneous insulin infusion (CSII).

Figure S7b. Subgroup meta-analysis of triglycerides (mmol/L) in patients during CIPII treatment compared to that during control treatment (CSII).

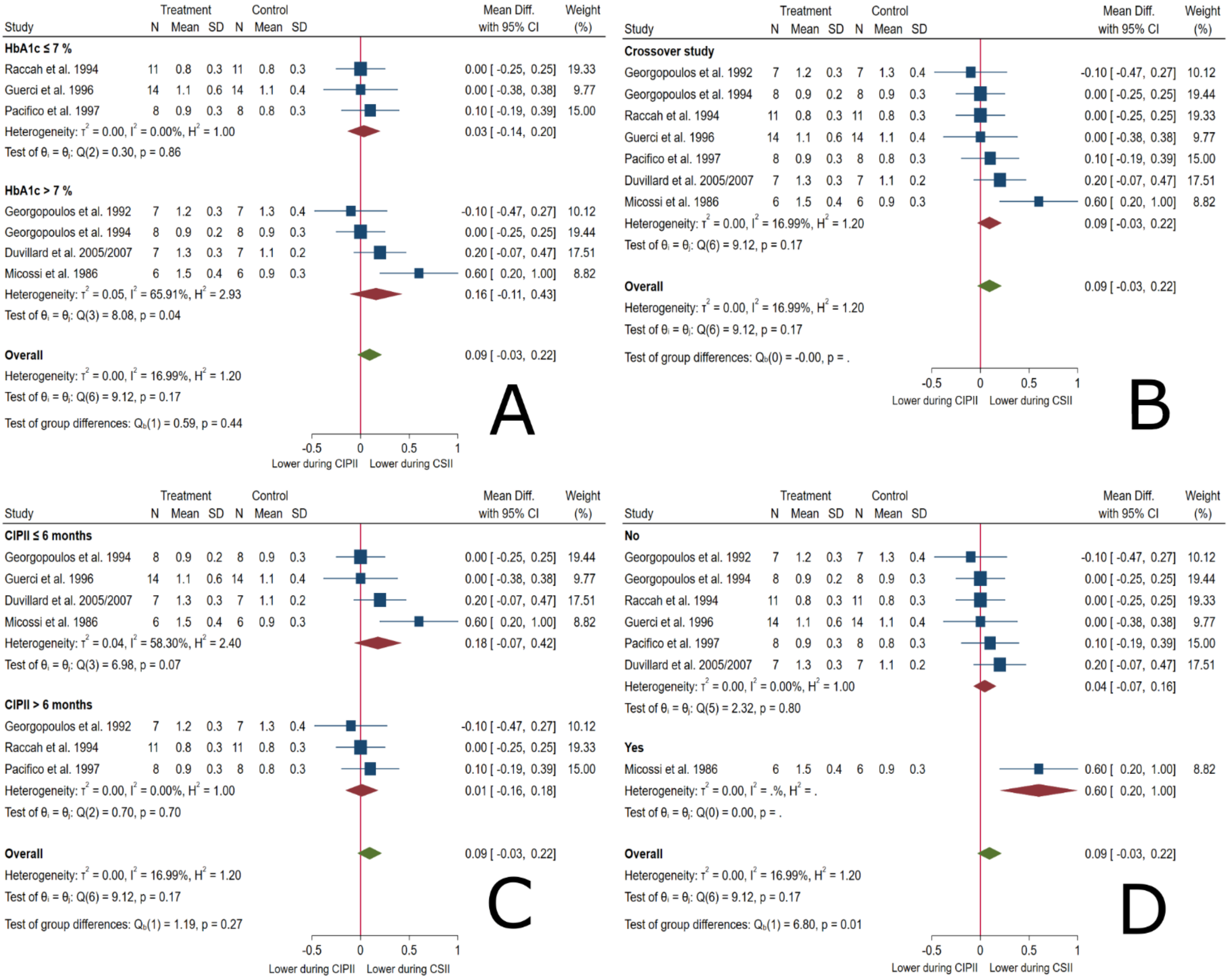

Legends: Treatment, continuous intraperitoneal insulin infusion (CIPII); Control, continuous subcutaneous insulin infusion (CSII). Figure A: Subgroup analysis according to HbA1c levels before starting CIPII treatment (≤ 7 % and > 7 %); Figure B: Subgroup analysis according to study type (Case-Control studies and Crossover studies); Figure C: Subgroup analysis according to length of the CIPII-period (≤ 6 months and > 6 months); Figure D: Subgroup analysis according to whether or not there was an additional controlled CSII follow-up-period with subsequent CIPII-period.

Figure S7c. Summarised subgroup meta-analysis of triglycerides (mmol/L) in patients during CIPII treatment compared to that during control treatment (CSII).

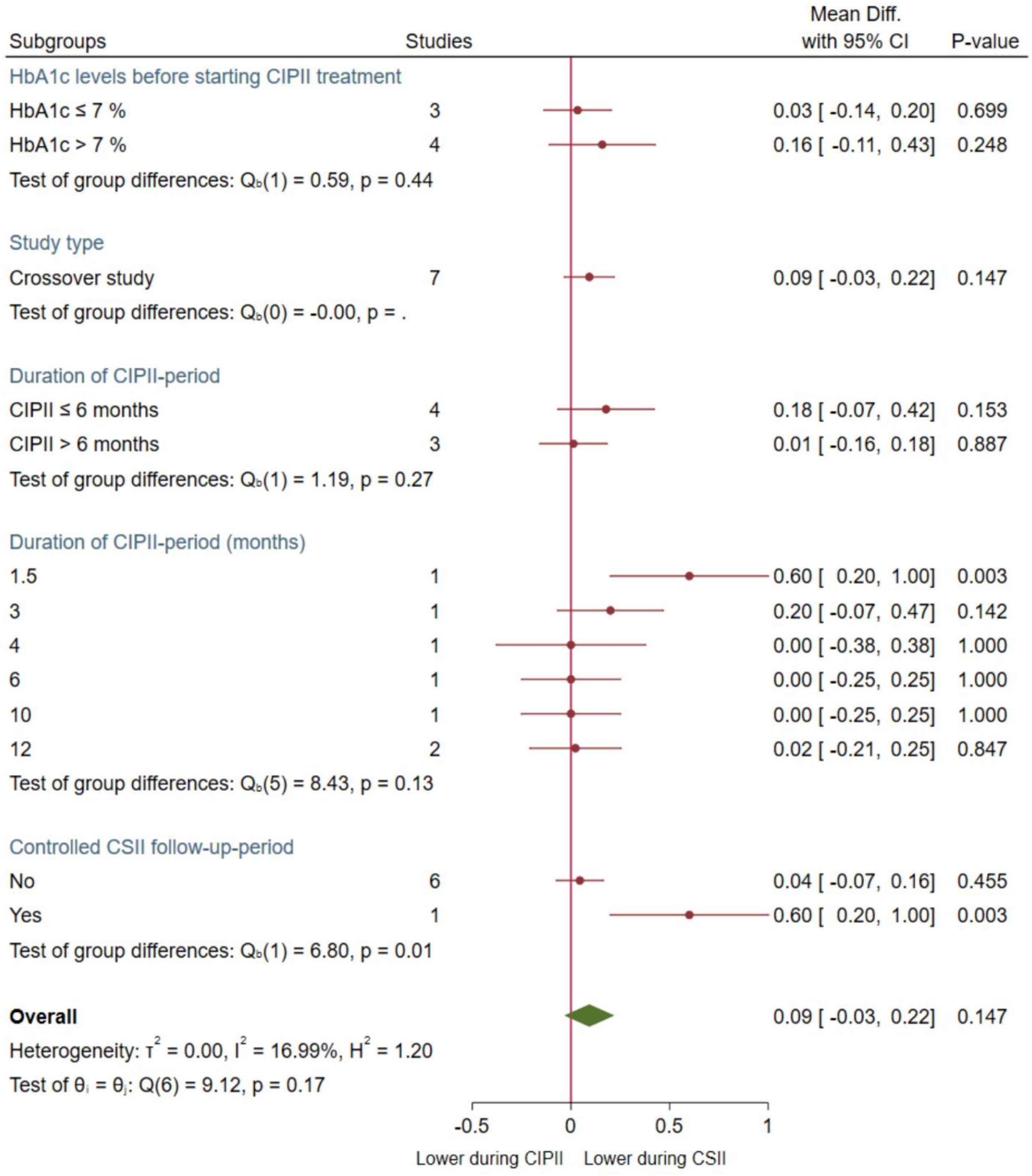

Legends: CIPII, continuous intraperitoneal insulin infusion; CSII, continuous subcutaneous insulin infusion.

Data for Egger`s test from STATA

HbA1c

meta bias, egger random(reml) tdistribution
Effect-size label: Mean Diff.
Effect size: _meta_es
Std. Err.: _meta_se
Regression-based Egger test for small-study effects
Random-effects model
Method: REML
H0: beta1 = 0; no small-study effects
beta1 = -1.10
SE of beta1 = 1.017
t = -1.08
Prob > t = 0.2932

Daily insulin dose

Model and method
Model: Random-effects
Method: REML
. meta bias, egger random(reml) tdistribution
Effect-size label: Mean Diff.
Effect size: _meta_es
Std. Err.: _meta_se
Regression-based Egger test for small-study effects
Random-effects model
Method: REML
H0: beta1 = 0; no small-study effects
beta1 = 0.43
SE of beta1 = 0.834
t = 0.51
Prob > t = 0.6212

References

1. Hofmann, H.M.H., P.A.M. Weiss, and J.G. Haas, *Continuous insulin delivery systems for the pregnant diabetic patient.* Acta Diabetologica Latina, 1986. **23**(3): p. 201-214.
2. Rautiainen, P., H. Tirkkonen, and T. Laatikainen, *Glycemic Control in Adult Type 1 Diabetes Patients with Insulin Glargine, Insulin Detemir, or Continuous Subcutaneous Insulin Infusion in Daily Practice.* Diabetes Technol Ther, 2018. **20**(5): p. 363-369.
3. Ruiz-de-Adana, M.S., et al., *Comparison between a multiple daily insulin injection regimen (basal once-daily glargine plus mealtime lispro) and continuous subcutaneous insulin infusion (lispro) using continuous glucose monitoring in metabolically optimized type 1 diabetes patients: A randomized open-labelled parallel study.* Med Clin (Barc), 2016. **146**(6): p. 239-46.
4. Rys, P.M., et al., *Continuous subcutaneous insulin infusion vs multiple daily injections in pregnant women with type 1 diabetes mellitus: a systematic review and meta-analysis of randomised controlled trials and observational studies.* Eur J Endocrinol, 2018. **178**(5): p. 545-563.
5. Liebl, A., et al., *A reduction in severe hypoglycaemia in type 1 diabetes in a randomized crossover study of continuous intraperitoneal compared with subcutaneous insulin infusion.* Diabetes, Obesity and Metabolism, 2009. **11**(11): p. 1001-1008.
6. Pitt, H.A., C.D. Saudek, and H.A. Zacur, *Long-term intraperitoneal insulin delivery.* Ann Surg, 1992. **216**(4): p. 483-91; discussion 491-2.
7. Schade, D.S., et al., *The peritoneal absorption of insulin in diabetic man: a potential site for a mechanical insulin delivery system.* Metabolism, 1979. **28**(3): p. 195-7.
8. *Advances in peritoneal dialysis : proceedings of the Second International Symposium on Peritoneal Dialysis : Berlin (-West), June 16-19, 1981 / editors, G.M. Gahl, M. Kessel, K.D. Nolph.* International congress series ; no. 567, ed. G.G. Gahl, 1938-, M.M. Kessel, 1926-, and K.D. Nolph. 1981, Amsterdam ; Princeton, NJ : Excerpta Medica ; New York, N.Y. : Sole distributors for the USA and Canada, Elsevier North-Holland, 1981.
9. Schade, D.S., et al., *Prolonged peritoneal insulin infusion in a diabetic man.* Diabetes Care, 1980. **3**(2): p. 314-317.
10. Schade, D.S., et al., *Normalization of plasma insulin profiles with intraperitoneal insulin infusion in diabetic man.* Diabetologia, 1980. **19**(1): p. 35-9.
11. Schade, D.S., et al., *Intraperitoneal delivery of insulin by a portable microinfusion pump.* Metabolism, 1980. **29**(8): p. 699-702.
12. Schade, D.S., R.P. Eaton, and N.M. Friedman, *Five-day programmed intraperitoneal insulin delivery in insulin-dependent diabetic man.* Journal of Clinical Endocrinology and Metabolism, 1981. **52**(6): p. 1165-1170.
13. Irsigler, K., et al., *Long-term continuous intraperitoneal insulin infusion with an implanted remote-controlled insulin infusion device.* Diabetes, 1981. **30**(12): p. 1072-5.
14. Selam, J.L., et al., *Total implantation of a remotely controlled insulin minipump in a human insulin-dependent diabetic.* Artif Organs, 1982. **6**(3): p. 315-9.
15. Selam, J.L., A. Slingeneyer, and B. Hedon, *Long-term ambulatory peritoneal insulin infusion of brittle diabetes with portable pumps: Comparison with intravenous and subcutaneous routes.* Diabetes Care, 1983. **6**(2): p. 105-111.
16. Fonseca, V.A., R.K. Menon, and P.M.S. O'Brien, *Diabetic pregnancy managed with intraperitoneal insulin.* Diabetic Medicine, 1987. **4**(1): p. 74-76.
17. Saudek, C.D., et al., *A preliminary trial of the programmable implantable medication system for insulin delivery.* N Engl J Med, 1989. **321**(9): p. 574-9.
18. Walter, H., et al., *Implantation of programmable infusion pumps for insulin delivery in type I diabetic patients.* Klinische Wochenschrift, 1989. **67**(11): p. 583-587.
19. Mirouze, J., et al., *Experience with external insulin pumps using the intraperitoneal route in 31 type I diabetic patients continuously followed for at least four years.* Diabetes, Nutrition and Metabolism - Clinical and Experimental, 1990. **3**(3): p. 185-189.
20. Selam, J.L., et al., *Clinical trial of programmable implantable insulin pump for type I diabetes.* Diabetes Care, 1992. **15**(7): p. 877-885.
21. Hopkins, K.D., et al., *Intraperitoneal insulin affects insulin-like growth factor binding protein-1 in a well-controlled type I diabetic patient.* Diabetes Care, 1993. **16**(10): p. 1404-5.
22. Bauersachs, R., et al., *Hormone and substrate levels after long-term continuous intraperitoneal insulin infusion in insulin-dependent diabetes mellitus.* Diab.Nurt.Metab, 1993. **6**: p. 25-32.

23. Olsen, C.L., et al., *Long-term safety and efficacy of programmable implantable insulin delivery systems.* International Journal of Artificial Organs, 1993. **16**(12): p. 847-854.
24. Haardt, M.J., et al., *A cost-benefit comparison of intensive diabetes management with implantable pumps versus multiple subcutaneous injections in patients with type I diabetes.* Diabetes Care, 1994. **17**(8): p. 847-857.
25. Ruotolo, G., et al., *Normalization of lipoprotein composition by intraperitoneal insulin in IDDM: Role of increased hepatic lipase activity.* Diabetes Care, 1994. **17**(1): p. 6-12.
26. Selam, J.L., et al., *Alterations in reverse cholesterol transport associated with programmable implantable intraperitoneal insulin delivery.* Metabolism: Clinical and Experimental, 1994. **43**(6): p. 665-669.
27. Bagdade, J.D., et al., *Intraperitoneal insulin therapy corrects abnormalities in cholesteryl ester transfer and lipoprotein lipase activities in insulin-dependent diabetes mellitus.* Arteriosclerosis and Thrombosis, 1994. **14**(12): p. 1933-1939.
28. Pinget, M., et al., *Multicentre trial of a programmable implantable insulin pump in type I diabetes.* International Journal of Artificial Organs, 1995. **18**(6): p. 322-325.
29. Hanaire-Broutin, H., et al., *Feasibility of intraperitoneal insulin therapy with programmable implantable pumps in IDDM. A multicenter study. The EVADIAC Study Group. Evaluation dans le Diabete du Traitement par Implants Actifs.* Diabetes Care, 1995. **18**(3): p. 388-92.
30. Jeandidier, N., et al., *Intraperitoneal insulin pump therapy during pregnancy: Two cases.* Practical Diabetes International, 1995. **12**(6): p. 280-280.
31. Jeandidier, N., et al., *Five cases of hyperthyroidism in type I diabetic patients treated with intraperitoneal insulin infusion.* Diabetes Care, 1995. **18**(6): p. 888-9.
32. Jeandidier, N., et al., *Immunogenicity of intraperitoneal insulin infusion using programmable implantable devices.* Diabetologia, 1995. **38**(5): p. 577-84.
33. Bagdade, J.D. and F.L. Dunn, *Improved lipoprotein surface and core lipid composition following intraperitoneal insulin delivery in insulin-dependent diabetes mellitus.* Diabetes and Metabolism, 1996. **22**(6): p. 420-426.
34. Logtenberg, S.J., et al., *Improved glycemic control with intraperitoneal versus subcutaneous insulin in type 1 diabetes: A randomized controlled trial.* Diabetes Care, 2009. **32**(8): p. 1372-1377.
35. Logtenberg, S.J., et al., *Health-related quality of life, treatment satisfaction, and costs associated with intraperitoneal versus subcutaneous insulin administration in type 1 diabetes: A randomized controlled trial.* Diabetes Care, 2010. **33**(6): p. 1169-1172.
36. Logtenberg, S.J.J., et al., *30 month post trial follow up of HbA1c with continuous intraperitoneal insulin infusion in type 1 diabetes.* Diabetologia, 2010. **53**: p. S8.
37. Schaepelynck, P., et al., *A recent survey confirms the efficacy and the safety of implanted insulin pumps during long-term use in poorly controlled type 1 diabetes patients.* Diabetes Technol Ther, 2011. **13**(6): p. 657-60.
38. Van Dijk, P.R., et al., *Effect of intraperitoneal insulin administration on IGF-1 concentrations in type 1 diabetes.* Diabetologia, 2012. **55**: p. S382.
39. van Dijk, P.R., et al., *Effect of i.p. insulin administration on IGF1 and IGFBP1 in type 1 diabetes.* Endocrine Connections, 2014. **3**(1): p. 17-23.
40. Van Dijk, P., et al., *Continuous intraperitoneal insulin infusion in type 1 diabetes: A 6 year post trial follow-up.* Diabetes Technology and Therapeutics, 2014. **16**: p. A20-A21.
41. Bilo, H.J.G., et al., *Continuous intraperitoneal insulin infusion versus subcutaneous insulin for type 1 diabetes: A prospective, case-control trial proving noninferiority.* Diabetologia, 2014. **1)**: p. S89.
42. van Dijk, P.R., et al., *Report of a 7 year case-control study of continuous intraperitoneal insulin infusion and subcutaneous insulin therapy among patients with poorly controlled type 1 diabetes mellitus: favourable effects on hypoglycaemic episodes.* Diabetes Res Clin Pract, 2014. **106**(2): p. 256-63.
43. van Dijk, P.R., et al., *After 6 years of intraperitoneal insulin administration IGF-I concentrations in T1DM patients are at low-normal level.* Growth Hormone and IGF Research, 2015. **25**(6): p. 316-319.
44. van Dijk, P.R., et al., *Intraperitoneal versus subcutaneous insulin therapy in the treatment of type I diabetes mellitus.* Netherlands Journal of Medicine, 2015. **73**(9): p. 399-409.
45. Van Dijk, P.R., et al., *Continuous intraperitoneal insulin infusion versus subcutaneous insulin therapy in the treatment of type 1 diabetes: Effects on glycemic variability.* Diabetes Technology and Therapeutics, 2015. **17**(6): p. 379-384.

46. van Dijk, P.R., et al., *Different routes of insulin administration do not influence serum free thiols in type 1 diabetes mellitus.* Endocrinol Diabetes Metab, 2019. **2**(4): p. e00088.
47. Boering, M., et al., *Effects of intraperitoneal insulin versus subcutaneous insulin administration on sex hormonebinding globulin concentrations in patients with type 1 diabetes mellitus.* Endocrine Connections, 2016. **5**(3): p. 136-142.
48. Mirouze, J., et al., *One year continuous run with a totally implantable insulin infusion pump in a human diabetic.* Transactions - American Society for Artificial Internal Organs, 1983. **29**: p. 709-713.
49. Rouaud, R., et al., *Long term ambulatory peritoneal insulin infusion operating life of the chronic catheters and the portable pumps* Pacing and Clinical Electrophysiology, 1988. **11**(6): p. 790-976.
50. van Dijk, P.R., et al., *Complications of continuous intraperitoneal insulin infusion with an implantable pump.* World journal of diabetes, 2012. **3**(8): p. 142-148.
51. Gin, H., et al., *Clinical evaluation of a newly designed compliant side port catheter for an insulin implantable pump: the EVADIAC experience. Evaluation dans le Diabete du Traitement par Implants Actifs.* Diabetes Care, 2001. **24**(1): p. 175.
52. Belicar, P. and V. Lassmann-Vague, *Local adverse events associated with long-term treatment by implantable insulin pumps. The French EVADIAC Study Group experience. Evaluation dans le Diabete du Traitement par Implants Actifs.* Diabetes Care, 1998. **21**(2): p. 325-6.
53. Renard, E., et al., *Insulin underdelivery from implanted pumps using peritoneal route. Determinant role of insulin pump compatibility.* Diabetes Care, 1996. **19**(8): p. 812-7.
54. Renard, E., et al., *Catheter Complications Associated With Implantable Systems for Peritoneal Insulin Delivery: An analysis of frequency, predisposing factors, and obstructing materials.* Diabetes Care, 1995. **18**(3): p. 300.
55. Mirouze, J., et al., *Clinical experience in human diabetics with portable and implantable insulin minipumps.* Life Support Syst, 1983. **1**(1): p. 39-49.
56. Irsigler, K. and H. Kritz, *On the clinical application of the insulin infusion in the open-loop-system. [German].* Zeitschrift fur die Gesamte Innere Medizin und Ihre Grenzgebiete, 1981. **36**(1): p. 8-19.
57. Liebl, A., et al., *Successful treatment of type 1 diabetes with intraperitoneal insulin infusion when subcutaneous insulin application is not possible: Two case reports.* Diabetes, 2012. **61**: p. A230-A231.
58. Schade, D.S., R.P. Eaton, and R.M. Warhol, *Subcutaneous peritoneal access device for type I diabetic patients nonresponsive to subcutaneous insulin.* Diabetes, 1982. **31**(5 I): p. 470-473.
59. Irsigler, K., et al., *Preprogrammed insulin infusion with a portable pump system.* Horm Metab Res Suppl, 1979(8): p. 193-7.
60. Selam, J.L., et al., *Randomized comparison of metabolic control achieved by intraperitoneal insulin infusion with implantable pumps versus intensive subcutaneous insulin therapy in type I diabetic patients.* Diabetes Care, 1992. **15**(1): p. 53-8.
61. Jeandidier, N., et al., *Decreased severe hypoglycemia frequency during intraperitoneal insulin infusion using programmable implantable pumps [1].* Diabetes Care, 1996. **19**(7): p. 780.
62. Renard, E., B. Guerci, and N. Jeandidier, *Long-term safety and efficacy of intraperitoneal insulin infusion from implanted pumps in a large series of patients with type 1 diabetes and initial high glucose variability.* Diabetologia, 2018. **61 (Supplement 1)**: p. S31.
63. Stephen, R.L., J.G. Maxwell, and J.J. Harrow, *Intervention in nephropathy due to insulin-dependent diabetes mellitus (IDDM).* Kidney International, 1985. **28**(SUPPL. 17): p. S-60-S-65.
64. Broussolle, C., N. Jeandidier, and H. Hanaire-Broutin, *French multicentre experience of implantable insulin pumps.* Lancet, 1994. **343**(8896): p. 514-515.
65. Olsen, C.L., et al., *Insulin antibody responses after long-term intraperitoneal insulin administration via implantable programmable insulin delivery systems.* Diabetes Care, 1994. **17**(3): p. 169-176.
66. Udelsman, R., et al., *Implanted programmable insulin pumps: one hundred fifty-three patient years of surgical experience.* Surgery, 1997. **122**(6): p. 1005-11.
67. Udelsman, R., et al., *Intraperitoneal delivery of insulin via mechanical pump: surgical implications.* Langenbecks Arch Surg, 2000. **385**(6): p. 367-72.
68. Dufaitre-Patouraux, L., et al., *Continuous intraperitoneal insulin infusion does not increase the risk of organ-specific autoimmune disease in type 1 diabetic patients: results of a multicentric, comparative study.* Diabetes Metab, 2006. **32**(5 Pt 1): p. 427-32.

69. DeVries, J.H., et al., *Continuous intraperitoneal insulin infusion in patients with 'brittle' diabetes: favourable effects on glycaemic control and hospital stay.* Diabet Med, 2002. **19**(6): p. 496-501.
70. Stephen, R., et al., *Long-term intraperitoneal insulin treatment: Preliminary Studies in 12 diabetic patients.".* Diabetic Renal-Retinal Syndrome, 1982. **2**: p. 447.
71. Campbell, I.W., et al., *Treatment of type I diabetic with subcutaneous insulin resistance by a totally implantable insulin infusion device ("Infusaid").* Diabetes Res, 1984. **1**(2): p. 83-8.
72. Othonos, N., et al., *Continuous intra-peritoneal insulin infusion: An alternative option for insulin administration.* Diabetic Medicine, 2017. **34**(Supplement 1): p. 16.
73. Schade, D.S., et al., *The intravenous, intraperitoneal, and subcutaneous routes of insulin delivery in diabetic man.* Diabetes, 1979. **28**(12): p. 1069-72.
74. Gooch, B.R., N.N. Abumrad, and R.P. Robinson, *Exercise in insulin-dependent diabetes mellitus: The effect of continuous insulin infusion using the subcutaneous, intravenous, and intraperitoneal sites.* Diabetes Care, 1983. **6**(2): p. 122-128.
75. Gooch, B.R., et al., *Near normalization of metabolism of IDDM: Comparison of continuous subcutaneous (CSII) versus intraperitoneal (CIPII) insulin delivery.* Hormone and Metabolic Research, 1984. **16**(SUPPL. 1): p. 190-194.
76. Dandona, P., V. Fonseca, and O. Fernando, *Control of diabetes through a subcutaneous peritoneal access device (SPAD) in patients with resistance to subcutaneous injected insulin.* Diabetes Research, 1987. **5**(1): p. 47-50.
77. Hermans, M.P., et al., *Fasting and postprandial plasma glucose and peripheral insulin levels in insulin-dependent diabetes mellitus and non-insulin-dependent diabetes mellitus subjects during continuous intraperitoneal versus subcutaneous insulin delivery.* Transplantation Proceedings, 1995. **27**(6): p. 3329-3330.
78. Dassau, E., et al., *Intraperitoneal insulin delivery provides superior glycaemic regulation to subcutaneous insulin delivery in model predictive control-based fully-automated artificial pancreas in patients with type 1 diabetes: a pilot study.* Diabetes Obes Metab, 2017. **19**(12): p. 1698-1705.
79. Lassmann-Vague, V., et al., *Immunogenicity of long-term intraperitoneal insulin administration with implantable programmable pumps: Metabolic consequences.* Diabetes Care, 1995. **18**(4): p. 498-503.
80. Lassmann-Vague, V., et al., *Autoimmunity and intraperitoneal insulin treatment by programmable pumps [14] (multiple letters).* Diabetes Care, 1998. **21**(11): p. 2041-2044.
81. Renard, E., et al., *Experience with intraperitoneal insulin infusion from implantable programmable systems in Type 1 (insulin-dependent) diabetes mellitus previously treated by external pumps.* Diabete et Metabolisme, 1993. **19**(4): p. 364-371.
82. Selam, J.L., et al., *Comparison of intraperitoneal and subcutaneous insulin administration on lipids, apolipoproteins, fuel metabolites, and hormones in type I diabetes mellitus.* Metabolism, 1989. **38**(9): p. 908-12.
83. Georgopoulos, A. and C.D. Saudek, *Normalization of composition of triglyceride-rich lipoprotein subfractions in diabetic subjects during insulin infusion with programmable implantable medication system.* Diabetes Care, 1992. **15**(1): p. 19-26.
84. Micossi, P., E. Bosi, and M. Cristallo, *Chronic continuous intraperitoneal insulin infusion (CIPII) in type I diabetic patients non-satisfactorily responsive to continuous subcutaneous insulin infusion (CSII).* Acta Diabetologica Latina, 1986. **23**(2): p. 155-164.
85. Service, F.J., et al., *Mean Amplitude of Glycemic Excursions, a Measure of Diabetic Instability.* Diabetes, 1970. **19**(9): p. 644-655.
86. Jeandidier, N., et al., *Comparison of intraperitoneal insulin infusion (using implantable pump) and subcutaneous insulin administration: Preliminary results of a crossover study.* Transplantation Proceedings, 1992. **24**(3): p. 948-949.
87. Lassmann-Vague, V., et al., *Insulin kinetics in type I diabetic patients treated by continuous intraperitoneal insulin infusion: Influence of anti-insulin antibodies.* Diabetic Medicine, 1996. **13**(12): p. 1051-1055.
88. Catargi, B., et al., *Comparison of blood glucose stability and HbA1c between implantable insulin pumps using U400 hoe 21pH insulin and external pumps using lispro in type 1 diabetic patients: A pilot study.* Diabetes and Metabolism, 2002. **28**(2): p. 133-137.
89. Oskarsson, P.R., et al., *Continuous intraperitoneal insulin infusion partly restores the glucagon response to hypoglycaemia in type 1 diabetic patients.* Diabetes Metab, 2000. **26**(2): p. 118-24.

90. Oskarsson, P.R., et al., *Metabolic and hormonal responses to exercise in type 1 diabetic patients during continuous subcutaneous, as compared to continuous intraperitoneal, insulin infusion.* Diabetes and Metabolism, 1999. **25**(6): p. 491-497.
91. Wredling, R., et al., *Experience of long-term intraperitoneal insulin treatment using a new percutaneous access device.* Diabetic Medicine, 1991. **8**(6): p. 597-600.
92. Wredling, R., et al., *Variation of insulin absorption during subcutaneous and peritoneal infusion in insulin-dependent diabetic patients with unsatisfactory long-term glycaemic response to continuous subcutaneous insulin infusion.* Diabete Metab, 1991. **17**(5): p. 456-9.
93. Van Dijk, P.R., et al., *Different effects of intraperitoneal and subcutaneous insulin administration on the GH-IGF-1 axis in type 1 diabetes.* Journal of Clinical Endocrinology and Metabolism, 2016. **101**(6): p. 2493-2501.
94. Liebl, A., et al., *Evaluation of the new Accu-Chek diaport, a port system for continuous intraperitoneal insulin infusion, in patients with type 1 diabetes: First 3-month results.* Diabetes Technology and Therapeutics, 2013. **15**: p. A13.
95. Liebl, A., et al., *Evaluation of the New ACCU-CHEK (R) DIAPORT, a Port System for Continuous Intraperitoneal Insulin Infusion, in Patients With Type 1 Diabetes: First 6-Month Results.* Diabetes 2013. **62**: p. pp.A247-A248.
96. Liebl , A., et al., *Evalution of the new ACCU-CHEK DIAPORT system, a port system for continous intraperitoneal insulin infusion, in patients with type 1 diabetes: final 12-month results*, in *International diabetes federation (IDF) World diabetes congress 2013, 2-6 December*. 2013: Melbourne, Australia.
97. Liebl, A., et al., *Long-term clinical evaluation of the new Accu-Chek diaport, a port system for continuous intraperitoneal insulin infusion: 24-month results.* Diabetes, 2014. **63**: p. A241.
98. Pacifico, A., et al., *Our experience with programmable implantable pump for intraperitoneal insulin infusion. [Italian].* Giornale Italiano di Diabetologia, 1997. **17**(1): p. 21-27.
99. Walter, H., et al., *Peripheral hyperinsulinemia in type I diabetics: Reduction via continuous infusion. [German].* Aktuelle Endokrinologie und Stoffwechsel, 1989. **10**(4): p. 224-228.
100. Giacca, A., et al., *Peritoneal and subcutaneous absorption of insulin in type I diabetic subjects.* J Clin Endocrinol Metab, 1993. **77**(3): p. 738-42.
101. Hanaire-Broutin, H., et al., *Effect of intraperitoneal insulin delivery on growth hormone binding protein, insulin-like growth factor (IGF)-I, and IGF-binding protein-3 in IDDM.* Diabetologia, 1996. **39**(12): p. 1498-1504.
102. Georgopoulos, A. and C.D. Saudek, *Intraperitoneal insulin delivery decreases the levels of chylomicron remnants in patients with IDDM.* Diabetes Care, 1994. **17**(11): p. 1295-1299.
103. Beylot, M., et al., *Insulin-mediated glucose disposal in type 1 (insulin-dependent) diabetic subjects treated by continous subcutaneous or intraperitoneal insulin fusion.* Diabete et Metabolisme, 1987. **13**(4): p. 450-456.
104. Lassmann-Vague, V., et al., *SHBG (sex hormone binding globulin) levels in insulin dependent diabetic patients according to the route of insulin administration.* Hormone and Metabolic Research, 1994. **26**(9): p. 436-437.
105. Schnell , O., et al., *Continuous intraperitoneal insulin therapy via port-system in type 1 diabetes with delayed absorption of subcutaneously applied insulin [Kontinuierliche intraperitoneale insulintherapie mit portsystem bei typ 1 diabetikern mit subkutaner insulinaufnahmestorung].* Diabetes und Stoffwechsel, 1994. **3**: p. 51-55.
106. Duvillard, L., et al., *Comparison of apolipoprotein B100 metabolism between continuous subcutaneous and intraperitoneal insulin therapy in type 1 diabetes.* Journal of Clinical Endocrinology and Metabolism, 2005. **90**(10): p. 5761-5764.
107. Duvillard, L., et al., *No change in apolipoprotein AI metabolism when subcutaneous insulin infusion is replaced by intraperitoneal insulin infusion in type 1 diabetic patients.* Atherosclerosis, 2007. **194**(2): p. 342-347.
108. Guerci, B., et al., *Intraperitoneal insulin infusion improves the depletion in choline- containing phospholipids of lipoprotein B particles in type I diabetic patients.* Metabolism: Clinical and Experimental, 1996. **45**(4): p. 430-434.
109. Raccah, D., et al., *Intraperitoneal insulin administration does not modify plasminogen activator inhibitor 1 levels in IDDM patients.* Diabetes Care, 1994. **17**(8): p. 941-2.
110. Arnqvist, H., et al., *Higher circulating IGF-I bioactivity and total IGF-I with intraperitoneal insulin delivery than with CSII in type 1 diabetes.* Growth Hormone and IGF Research, 2010. **20**: p. S24-S25.
111. Hedman, C.A., et al., *Intraperitoneal insulin delivery gives higher circulating IGF-I activity than CSII in type 1 diabetes.* Diabetologia, 2009. **52 (S1)**: p. S376-S377.

112. Hedman, C.A., et al., *Intraperitoneal insulin delivery to patients with type 1 diabetes results in higher serum IGF-I bioactivity than continuous subcutaneous insulin infusion.* Clin Endocrinol (Oxf), 2014. **81**(1): p. 58-62.
113. Catargi, B., et al., *Glucose profiles in a type 1 diabetic patient successively treated with CSII using regular insulin, lispro and an implantable insulin pump.* Diabetes Metab, 2000. **26**(3): p. 210-4.
114. Colette, C., et al., *Effect of different insulin administration modalities on vitamin D metabolism of insulin-dependent diabetic patients.* Hormone and Metabolic Research, 1989. **21**(1): p. 37-41.
115. Jeandidier, N., et al., *Comparison of antigenicity of Hoechst 21PH insulin using either implantable intraperitoneal pump or subcutaneous external pump infusion in type 1 diabetic patients.* Diabetes Care, 2002. **25**(1): p. 84-8.
116. Arnqvist, H., et al., *OR9,53 Higher circulating IGF-I bioactivity and total IGF-I with intraperitoneal insulin delivery than with CSII in type 1 diabetes.* Growth Hormone & IGF Research, 2010. **20**: p. S24-S25.
117. van Dijk, P.R., et al., *Favourable serum calcification propensity with intraperitoneal as compared with subcutaneous insulin administration in type 1 diabetes.* Ther Adv Endocrinol Metab, 2020. **11**: p. 2042018820908456.
